# Supplementary material for: Bilirubin gates the TRPM2 channel as a direct agonist to exacerbate ischemic brain damage
Source: Neuron. 2023 May 17;111(10):1609–1625.e6. doi: 10.1016/j.neuron.2023.02.022 (PMC10191619; doi:10.1016/j.neuron.2023.02.022)
Supplement: Document S1. Figures S1–S11 and Tables S1–S4 [file mmc1.pdf]

Neuron, Volume *111*

Supplemental information

Bilirubin gates the TRPM2 channel as a direct agonist to exacerbate ischemic brain damage

Han-Wei Liu, Li-Na Gong, Ke Lai, Xia-Fei Yu, Zhen-Qi Liu, Ming-Xian Li, Xin-Lu Yin, Min Liang, Hao-Song Shi, Lin-Hua Jiang, Wei Yang, Hai-Bo Shi, Lu-Yang Wang, and Shan-Kai Yin

Figure S1

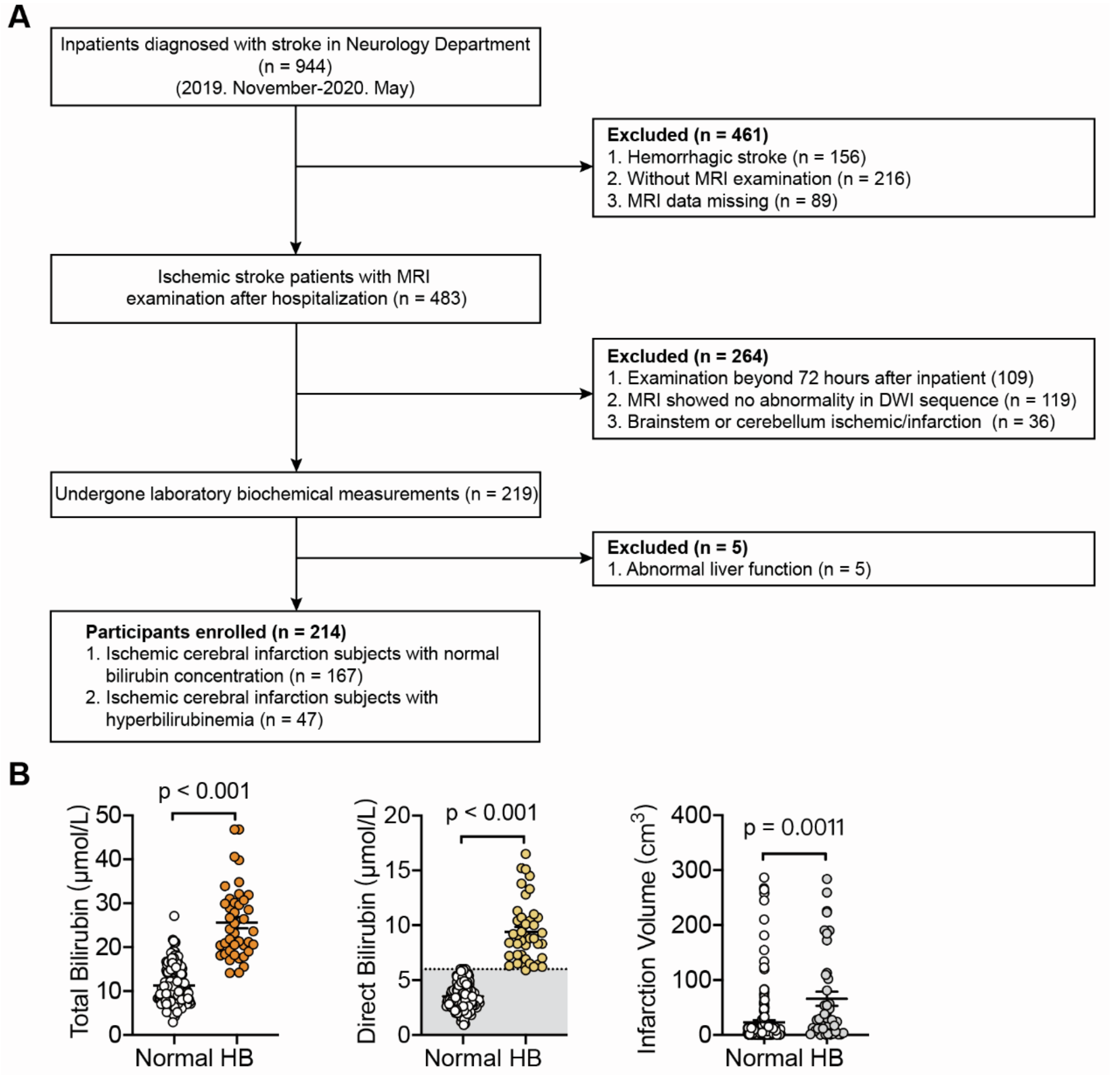

Figure S1. Enrollment flow chart for the study population, Related to Figure 1.

(A) 944 subjects diagnosed with stroke in neurology department from November 2019 to May 2020 were recruited in this study cohort. 214 ischemic stroke inpatients met the inclusion criteria and were enrolled in this study.

(B) Summary plots of TB, DB and infarct volume of enrolled patients which were grouped according to the DB concentration (Normal: DB ≤ 6 μmol/L, n = 173; HB: DB > 6 μmol/L, n = 41).

Figure S2

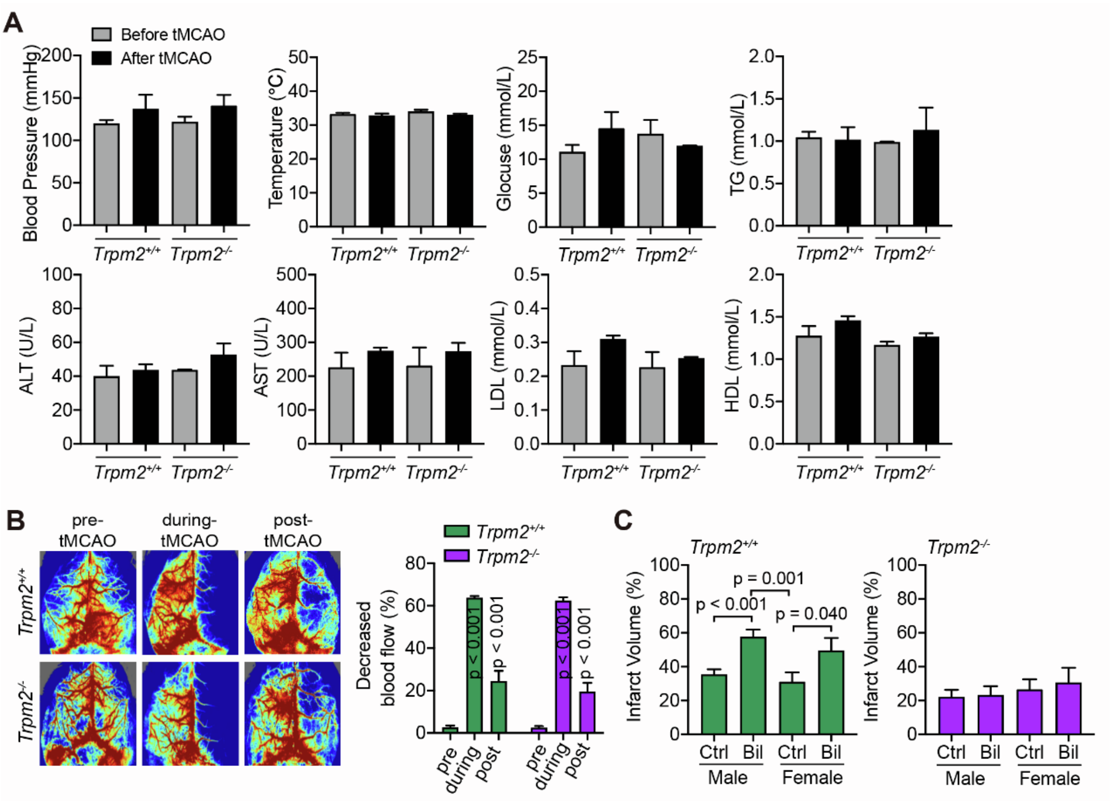

Figure S2. Genotype-dependent differences in the infract volume of *Trpm2*$^{+/+}$ and *Trpm2*$^{-/-}$ mice both sexes by tMCAO surgery without affecting major physiological and biochemical parameters, Related to Figure 2.

(A) Measurement results of blood pressure, body temperature and serum biochemical indexes (triglyceride, TG; alanine aminotransferase, ALT; aspartate aminotransferase, AST; low density lipoprotein, LDL; high density lipoprotein, HDL) before and after tMCAO surgery (n = 3)

(B) Representative blood flow images of the brain in mice before and after tMCAO surgery and flux (%) was quantified with a laser Doppler flowmetry probe (n = 5).

(C) Summary plots of the infarction volume of *Trpm2*$^{+/+}$ and *Trpm2*$^{-/-}$ male and female mice 24h after tMCAO surgery. In *Trpm2*$^{+/+}$ mice, bilirubin significantly increased the infarct volume in both male and female mice (Male: Ctrl: 35.42 ± 3.01%, Bil: 57.61 ± 4.24%, n = 10; Female: Ctrl: 31.08 ± 5.55%, Bil: 49.56 ± 7.40%, n = 4). Female mice showed slightly smaller infract volume which was not statistical different from male mice. Bilirubin showed little effect on aggravating brain injury in *Trpm2*$^{-/-}$ mice of both sexes (Male: Ctrl: 22.11 ± 4.14%, Bil: 23.22 ± 5.25%, n = 10; Female: Ctrl: 26.50 ± 6.02%, Bil: 30.63 ± 8.71%, n = 5).

Error bars represent means ± SEM; one-way ANOVA with post hoc LSD test.

Figure S3

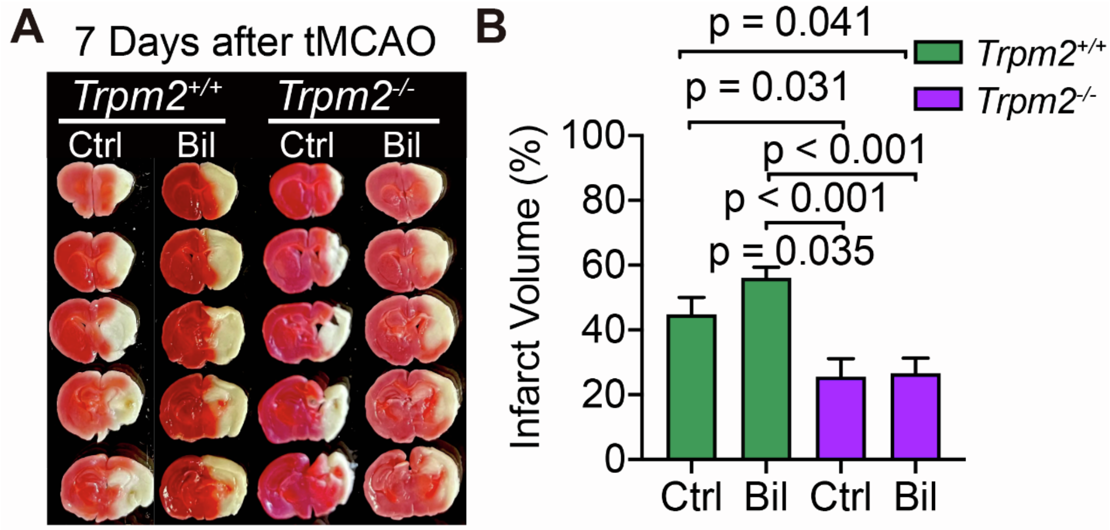

Figure S3. Transient hyperbilirubinemia aggravates long-term ischemic brain injury in genotype-specific manner, Related to Figure 2.

(A) Representative images of brain sections by TTC staining of *Trpm2*$^{+/+}$ and *Trpm2*$^{-/-}$ mice 7 days after the tMCAO surgery.

(B) Summary data showing normalized infarct volumes of Ctrl and Bil group in *Trpm2*$^{+/+}$ and *Trpm2*$^{-/-}$ mice (n=5).

Error bars represent means ± SEM; one-way ANOVA with post hoc LSD test.

Figure S4

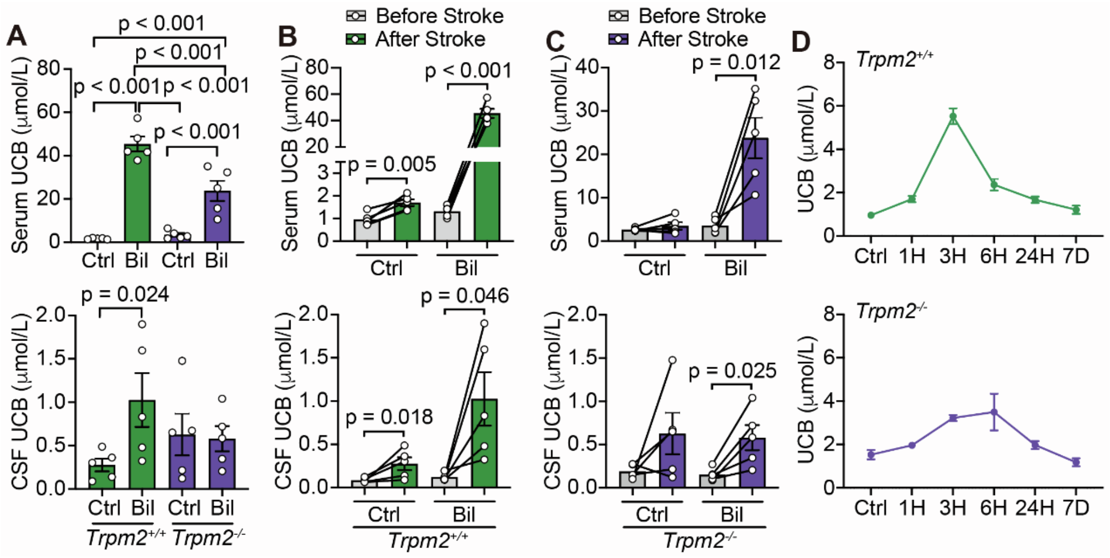

Figure S4. Temporal profiles of UCB concentrations after tMCAO surgery, Related to Figure 2.

(A) Summary data showing serum and CSF UCB concentrations of Ctrl and Bil group in *Trpm2*$^{+/+}$ and *Trpm2*$^{-/-}$ mice 1hour after tMCAO surgery (n= 5).

(B and C) Comparison of UCB concentrations in serum and CSF of in *Trpm2*$^{+/+}$ and *Trpm2*$^{-/-}$ mice in Ctrl and Bil group before and after ischemia-reperfusion injury (n = 5).

(D) Summary data showing the time-course of UCB concentrations in serum before (Ctrl) and after the tMCAO surgery in Trpm2+/+ and Trpm2-/- mice, respectively (n = 3 to 10) (basal bilirubin concentrations were indicated by Ctrl).

Error bars represent means ± SEM; paired Student's t test, one-way ANOVA with post hoc LSD test.

Figure S5

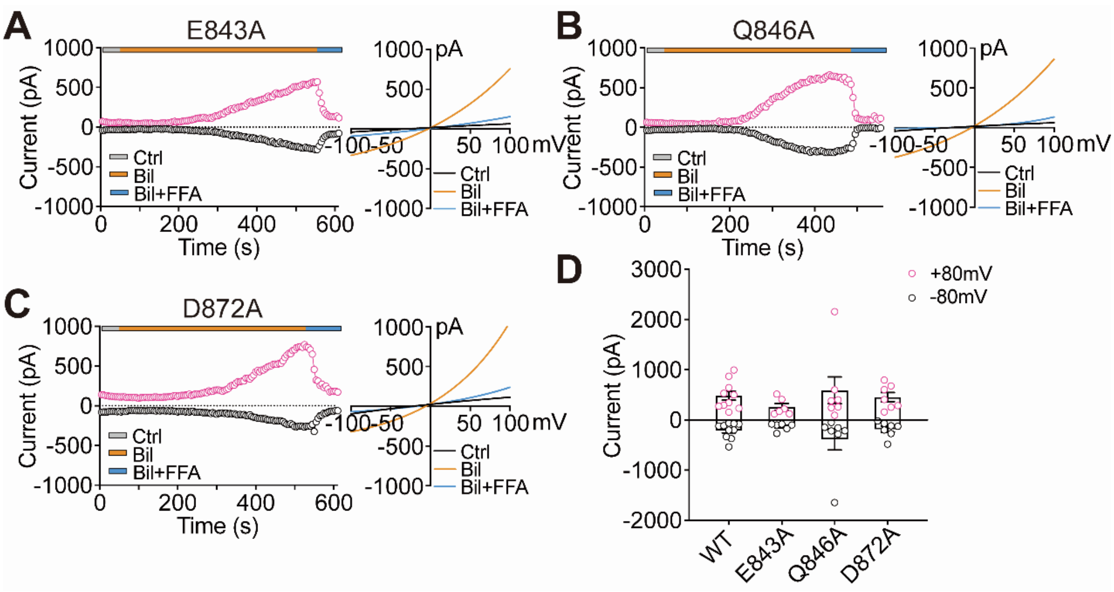

Figure S5. Bilirubin activates TRPM2 currents in Ca^{2+} binding site mutants, Related to Figure 4.
(A to C) Representative time-course of currents at +80 mV (strawberry circle) and -80 mV (black circle) and ramp current traces transformed into superimposed I-V curves currents from Ca^{2+} binding site mutants before and after bilirubin application and FFA co-application.
(D) Summary data showing the maximum amplitude of currents (at +80 mV and -80 mV) in mutant TRPM2 channels activated by bilirubin in A to C relative to the that in WT channels (n = 6 to 13). Error bars represent means ± SEM; unpaired Student's t test.

Figure S6

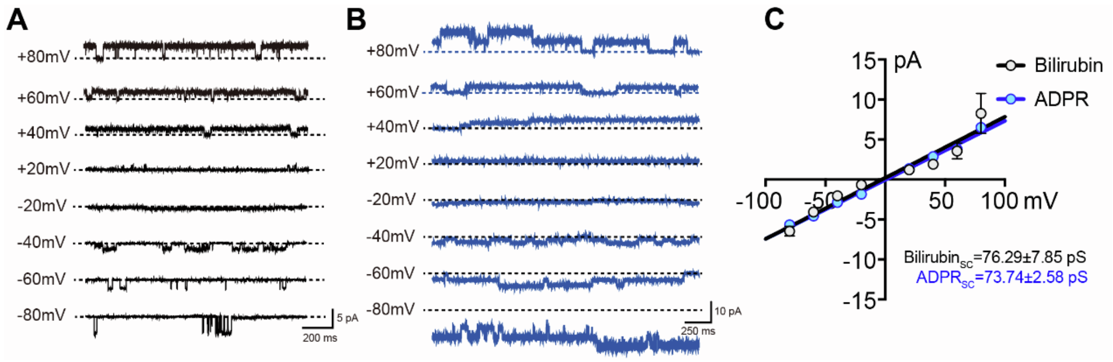

Figure S6. Comparable single channel conductance of the TRPM2 current activated by bilirubin and ADPR, Related to Figure 4.

(A and B) Representative TRPM2 single-channel recordings activated by bilirubin and ADPR from -80 mV to +80 mV.

(C) Mean single-channel current amplitudes activated by bilirubin and ADPR were plotted against membrane holding potentials and fitted with linear regression to calculate single channel conductance as given in the inset (n = 4 to 7).

Figure S7

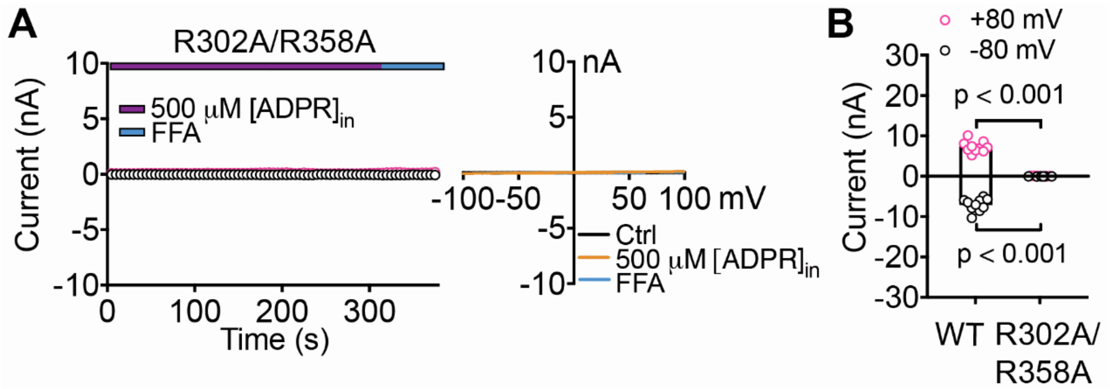

Figure S7. The R302A/R358A mutation abolishes ADPR activation of the TRPM2 channel, Related to Figure 6.

(A) Representative time-course of currents at +80 mV (strawberry circle) and -80 mV (black circle) and ramp current traces transformed into superimposed I-V curves currents R302A/R358A double mutant.

(B) Summary data showing the maximum amplitude of currents (at +80 mV and -80 mV) activated by ADPR in WT and R302A/R358A mutant TRPM2 channels (n = 5 and 10).

Error bars represent means ± SEM; unpaired Student's t test.

Figure S8

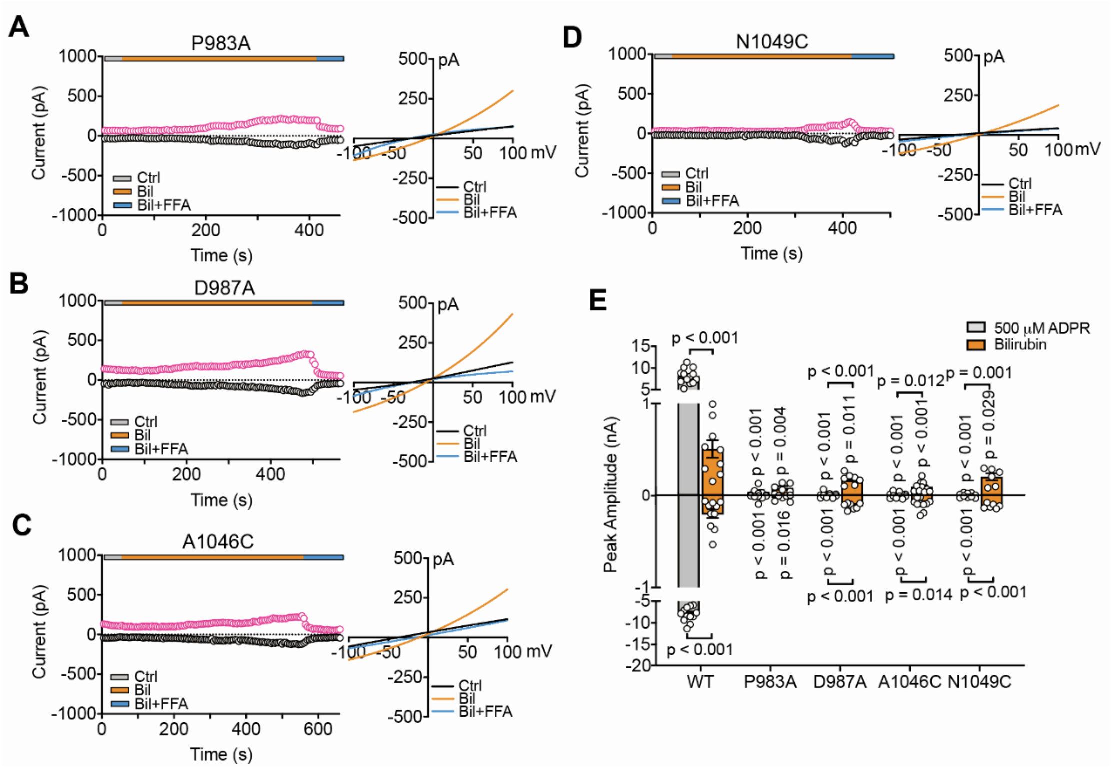

Figure S8. bilirubin activates TRPM2 pore domain mutants, Related to Figure 6.

(A to D) Representative time-course of currents at +80 mV (strawberry circle) and -80 mV (black circle) and superimposed I-V curves from ion-conducting pore mutants (top of the pore: P983A and D987A, bottom of the pore: A1046C and N1049C) before and after bilirubin application and FFA co-application.

(E) Summary data showing the maximum amplitude of currents (at +80 mV and -80 mV) activated by ADPR and bilirubin in WT TRPM2 channel and pore domain mutants (n = 6 to 13).

Error bars represent means ± SEM; unpaired Student's t test.

Figure S9

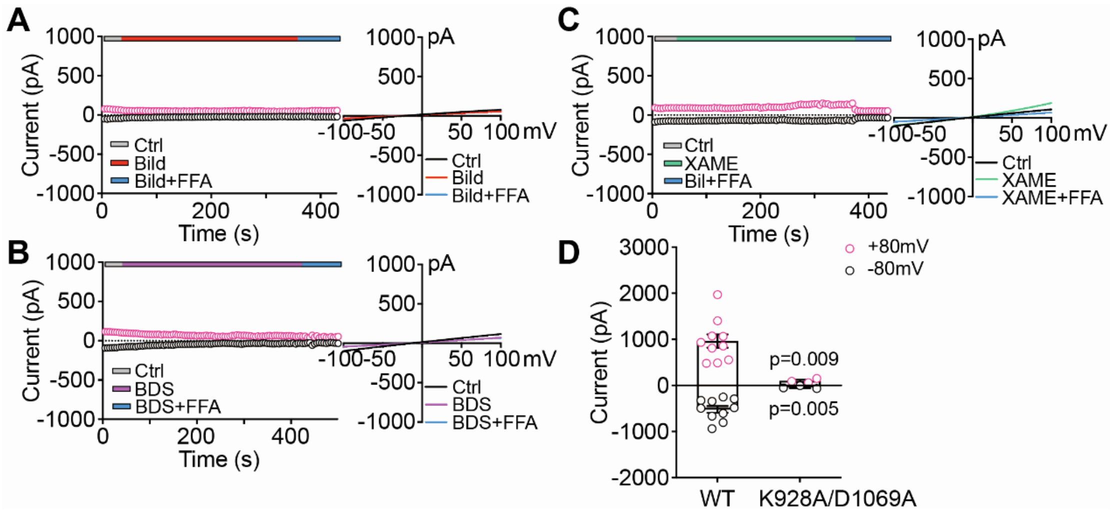

Figure S9. Derivatives of bilirubin fails to open TRPM2 channel in K928A/D1069A double mutant, Related to Figure 6.

(A to C) Representative time-course of currents at +80 mV (strawberry circle) and -80 mV (black circle) before and after bilirubin (9 μM), BDS (9 μM) and XAME (9 μM), followed by FFA co-application. I-V relationships before and after drug applications are overlayed and presented on the right panels.

(D) Summary data showing the maximum amplitude of currents activated by XAME in WT TRPM2 channel and K928A/D1069A mutant (n = 3 and 10).

Error bars represent means ± SEM; unpaired Student's t test.

Figure S10

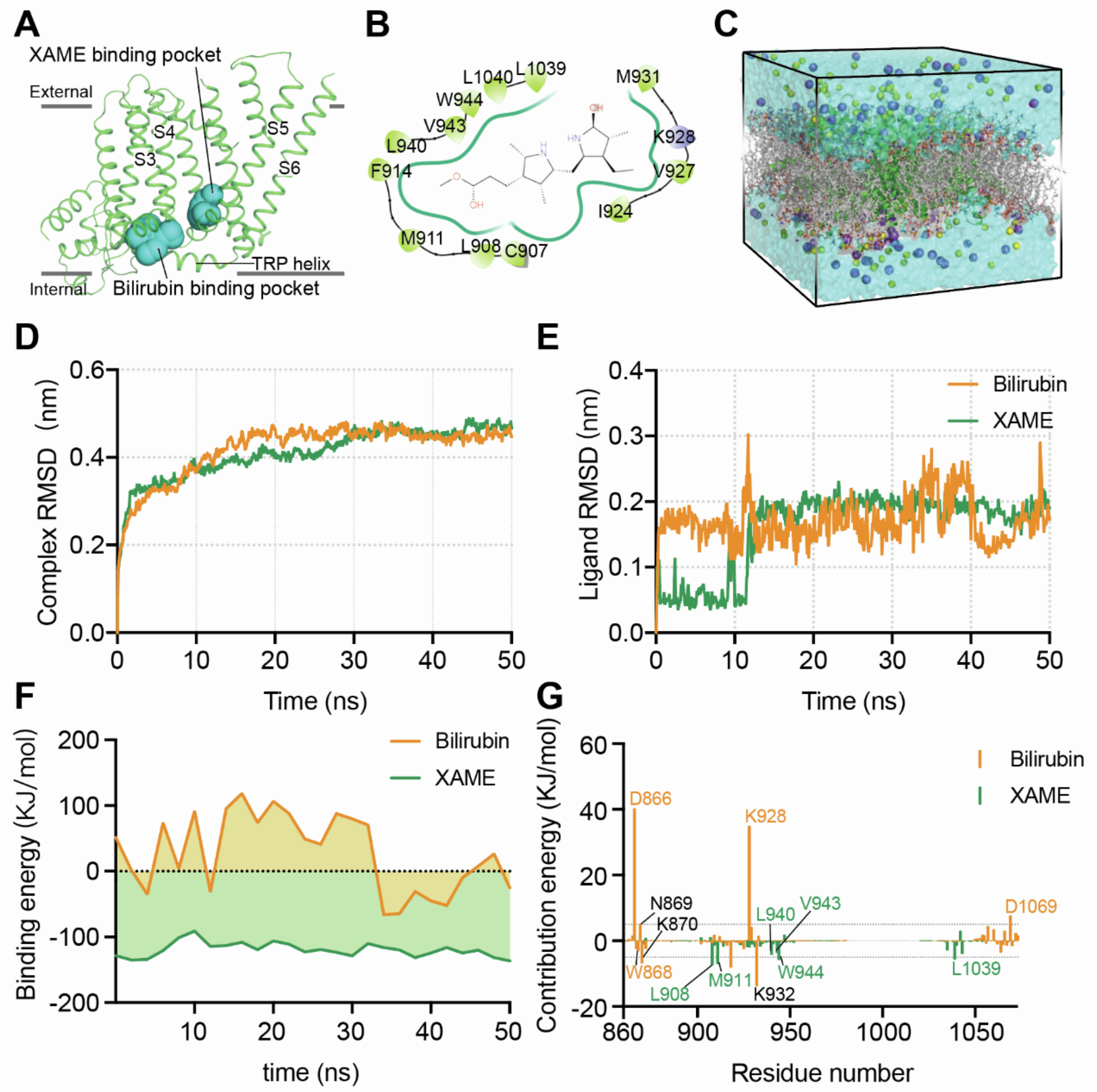

Figure S10. Molecular dynamics (MD) simulation analyses of the binding pockets of bilirubin and XAME and their interactions with the TRPM2 channel, Related to Figure 6.

(A) Relative positions of the binding pockets of bilirubin and XAME to the TRPM2 channel.

(B) 2D Interaction map between XAME and amino acid residues in the binding pocket.

(C) The simulated state diagram of the TRPM2-ligand complexes embedded in the cell membrane phospholipid bilayer.

(D and E) Summary results of structural stability analyses (root mean square deviation, RMSD) of channel-ligand complexes and ligands following bilirubin and XAME binding to TRPM2 channels in 50 ns bouts.

(F) Binding energy calculations showing a higher affinity for XAME than bilirubin to the TRPM2 channel.

(G) Summary analysis of binding energies contributed by the key residues within the binding pocket of TRPM2-bilirubin and TRPM2-XAME complexes (residues interact with bilirubin were shown in orange, and those of XAME were shown in green).

Figure S11

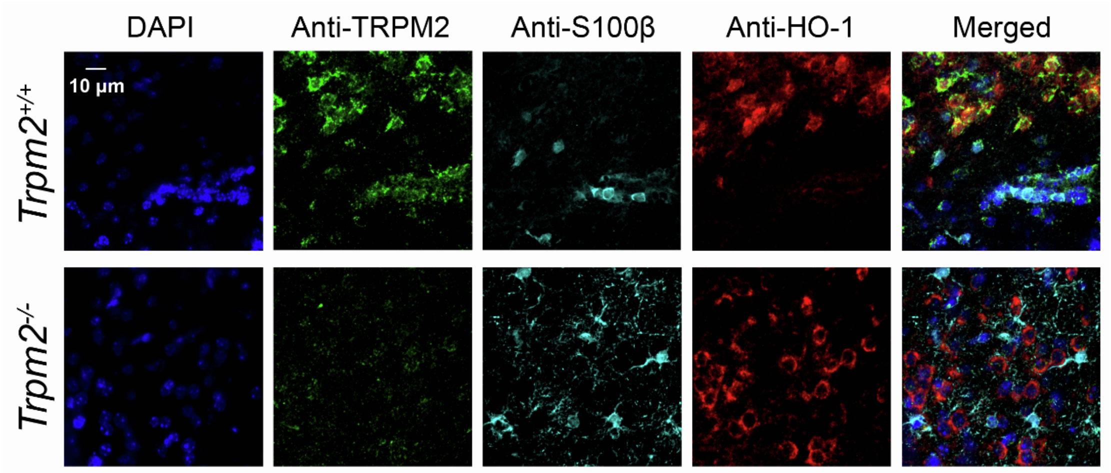

Figure S11. Astrocytes lack the expression of a key enzyme in bilirubin metabolism, Related to Figure 7.

Immunofluorescence co-localization imaging of astrocyte (anti-S100β), TRPM2 channels (anti-TRPM2), and heme oxygenase 1 (anti-HO-1) in cortical slices from Trpm2$^{+/+}$ and Trpm2$^{-/-}$ mice. Despite the presence the TRPM2 channel in neurons and astrocytes, HO-1, the key enzyme for bilirubin metabolism, was not colocalized with astrocytes.

Table S1

	n	Age (year)	Gender (male/n%)	TB (μmol/L)	DB (μmol/L)	Infarct volume (cm^3)
Normal group	167	71 (25-95)	106 (63.47%)	10.74 (2.90-17.90)	3.52 (0.90-9.00)	18.45 (0.06-267.04)
HB group	47	68 (32-88)	36 (76.60%)	25.43 (18.10-46.80)	8.65 (4.30-16.50)	76.42 (0.13-286.65)
RR	/	/	/	0-18.0	0-6.0	/

Table S1. Detail information for enrolled stroke patients, Related to Figure 1.
Normal group was defined as serum TB=0-18.0 μmol/L. HB group was defined as serum TB beyond the reference range (RR).

Table S2

Residue	E_{Vdw} (kcal/mol)	E_{coul} (kcal/mol)	E_{Hbond} (kcal/mol)	Dis (Å)	E_{total} (kcal/mol)
K928	1.166 ± 1.113	93.804 ± 10.215	0	1.182 ± 0.154	94.032 ± 9.067
D1069	7.164 ± 0.543	28.723 ± 2.942	0.505 ± 0.190	2.063 ± 0.160	36.392 ± 2.817
D866	3.219 ± 1.674	32.650 ± 3.531	0.246 ± 0.118	2.164 ± 0.194	36.116 ± 3.687
W868	3.984 ± 1.003	4.688 ± 2.322	0.174 ± 0.188	1.989 ± 0.335	8.845 ± 1.970

Table S2. Interaction scores between bilirubin and key residues of TRPM2 channel in molecular docking, Related to Figure 6.

In silico simulations between bilirubin with its binding pocket of TRPM2 channel. Bilirubin interacts with four residues among S3, S5 and TRP helix. Internal space constrains (Distance or Dis in Angstrom or Å) and molecular energetics of chemical bonds include Van Der Walls (Ev_{dw}) force, Coulombic (E_{coul}) force and hydrogen bond (E_{Hbond}) are showed in the table (distance and energy of the top 8 bilirubin poses interacting with TRPM2 residues are shown as mean ± s.d.). The spatial distance and cumulative interaction energy (E_{total}) between bilirubin and the binding sites were also calculated.

Table S3

	Bilirubin binding pocket	XAME binding pocket
Pocket volume (Å^3)	2039.860	1452.90
Ligand volume (Å^3)	529.150	352.440
Ligand-Pocket overlap percent (%)	100	100
Total score	22.701	25.885
Polarity score	7	3
Flexibility	0.391	0.566

Table S3. Information on the binding pockets of bilirubin and XAME with the TRPM2 channel, Related to Figures 6.

In MD simulations of interactions between bilirubin or XAME and the TRPM2 channel, only the transmembrane domain of TRPM2 was selected. The relevant parameters of the bilirubin and XAME binding pockets were analyzed separately, including pocket volume, ligand volume and ligand-pocket overlap percentage. The total score, pockets polarity and flexibility were also analyzed and given.

Table S4

Species	Gene ID	Peptide ID	Peptide length
Human (*Homo sapiens*)	ENSG00000142185	ENSP00000381023	1503aa
Mouse (*Mus musculus*)	ENSMUSG00000009292	ENSMUSP00000101040	1506aa
Partial Sequence Information (*Homo sapiens*)	3101 TCTTCACCAACATCCTGCTGCTCAACCTCCTCATCGCCATGTTCAACTACACCTTCCAGC 3160<br>1034 L - - F - - T - - N - - I - - L - - L - - L - - N - - L - - I - - A - - M - - F - - N - - Y - - T - - F - - Q - - 1053<br>3161 AGGTGCAGGAGCACACGGACCAGATTTGGAAGTTCCAGCGCCATGACCTGATCGAGGAGT 3220<br>1054 Q - - V - - Q - - E - - H - - T - - D - - Q - - I - - W - - K - - F - - Q - - R - - H - - D - - L - - I - - E - - E - - 1073<br>3221 ACCACGGCCGCCCCGCCGCGCCGCCCCCCTTCATCCTCCTCAGCCACCTGCAGCTCTTCA 3280<br>1075 Y - - H - - G - - R - - P - - A - - A - - P - - P - - P - - F - - I - - L - - L - - S - - H - - L - - Q - - L - - F - - 1093		
Partial Sequence Information (*Mus musculus*)	3122 TCATCGCCATGTTCAACTACACCTTCCAGGAGGTGCAGGAACACACAGACCAGATCTGGA 3181<br>1041 L - - I - - A - - M - - F - - N - - Y - - T - - F - - Q - - E - - V - - Q - - E - - H - - T - - D - - Q - - I - - W - - 1060<br>3182 AATTCCAGCGCCACGACCTGATCGAGGAGTACCATGGCCGTCCCCCGGCACCTCCCCCAC 3241<br>1061 K - - F - - Q - - R - - H - - D - - L - - I - - E - - E - - Y - - H - - G - - R - - P - - P - - A - - P - - P - - P - - 1080<br>3242 TCATCCTCCTCAGCCACCTGCAGCTCCTGATCAAGAGGATTGTCCTGAAGATCCCTGCCA 3301<br>1081 L - - I - - L - - L - - L - - S - - H - - L - - Q - - L - - L - - L - - K - - R - - I - - V - - L - - K - - I - - P - - A - - 1100		
Exon 23	CTACACCTTCCAGGAGGTGCAGGAACACACAGACCAGATCTGGAAATTCCAGCGCCACGACCTGATC<br>GAGGAGTACCATGGCCGTCCCCCGGCACCTCCCCCACTCATCCTCCTCAGCCACCTGCAGCTCCTGAT<br>CAAGAGGATTGTCCTGAAGATCCC TGCCAAGAGGCATAAGCAGCTCA		
Guide RNA	GGTACTCCTCGATCAGGTCG TGG		
Donor DNA	CTACACCTTCCAGGAGGTGCAGGAACACACAGACCAGATCTGGAAATTCCAGCGCCACGCCCTGATC<br>GAGGAGTACCATGGCCGTCCCCCGGCACCTCCCCCACTCATCCTCCTCAGCCACCTGCAGCTCCTGAT<br>CAAGAGGATTGTCCTGAAGATCCCTGCCAAGAG GCATAAGCAGCTCA		

Table S4. Related sequence information of transgenic mice, Related to Figure 8 and STAR Methods.

The human and mouse gene ID, peptide ID, peptide length and partial gene/peptide sequence information are shown, in which the gene sequence is shown in black, the peptide sequence is shown in blue, and the gene sequence and peptide sequence corresponding to D1069/D1066 are shown in green background and yellow background respectively. The sequence information of Guide RNA and Donor DNA designed for mouse Exon 23 are also listed, among which the genetically modified codons are marked with red underlines, and the mutated gene are marked with red background.
