# Supplementary material for: Bilirubin gates the TRPM2 channel as a direct agonist to exacerbate ischemic brain damage
Source: Neuron. 2023 May 17;111(10):1609–1625.e6. doi: 10.1016/j.neuron.2023.02.022 (PMC10191619; doi:10.1016/j.neuron.2023.02.022)
Supplement: Document S2. Article plus supplemental information [file mmc2.pdf]

Article

Neuron

Bilirubin gates the TRPM2 channel as a direct agonist to exacerbate ischemic brain damage

Graphical abstract

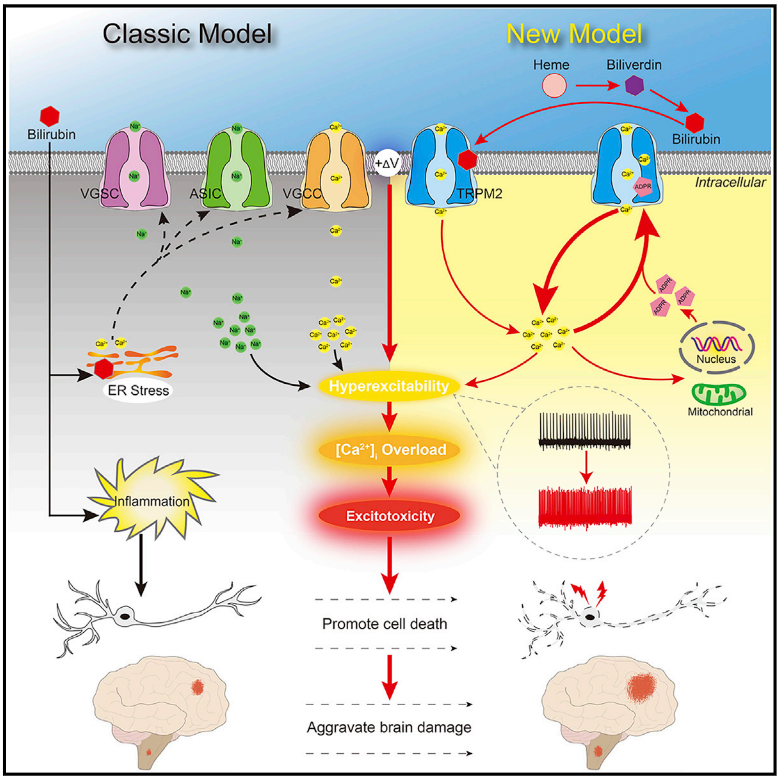

Highlights

- Infarct size scales with bilirubin level in stroke patients and mouse models
- Bilirubin activates the TRPM2 channel as an agonist to exacerbate neurotoxicity
- The TRPM2 channel harbors a specific binding cavity for bilirubin
- The bilirubin-binding cavity is a potential site for developing stroke therapeutics

Authors

Han-Wei Liu, Li-Na Gong, Ke Lai, ..., Hai-Bo Shi, Lu-Yang Wang, Shan-Kai Yin

Correspondence

hbshi@sjtu.edu.cn (H.-B.S.), luyang.wang@utoronto.ca (L.-Y.W.), skyin@sjtu.edu.cn (S.-K.Y.)

In brief

Liu et al. discover that bilirubin directly gates the opening of TRPM2 channels as an endogenous agonist to aggravate brain damage in ischemic stroke. Molecular perturbation to or blockade of the bilirubin-binding cavity on the TRPM2 channel provides effective neuroprotection in mouse models.

Liu et al., 2023, Neuron *111*, 1609–1625
May 17, 2023 © 2023 The Author(s). Published by Elsevier Inc.
https://doi.org/10.1016/j.neuron.2023.02.022

CellPress

Neuron

CellPress
OPEN ACCESS

Article

Bilirubin gates the TRPM2 channel as a direct agonist to exacerbate ischemic brain damage

Han-Wei Liu,[1,9] Li-Na Gong,[1,9] Ke Lai,[1,2,3,9] Xia-Fei Yu,[4] Zhen-Qi Liu,[1,2,3] Ming-Xian Li,[1] Xin-Lu Yin,[1,5] Min Liang,[1,6] Hao-Song Shi,[1] Lin-Hua Jiang,[7,8] Wei Yang,[4] Hai-Bo Shi,[1,*] Lu-Yang Wang,[2,3,10,*] and Shan-Kai Yin[1,*]
[1]Department of Otorhinolaryngology Head & Neck Surgery, Shanghai Sixth People's Hospital and Shanghai Jiao Tong University School of Medicine, Shanghai 200233, China
[2]Program in Neuroscience and Mental Health, SickKids Research Institute, Toronto, ON M5G 1X8, Canada
[3]Department of Physiology, University of Toronto, Toronto, ON M5S 1A8, Canada
[4]Department of Biophysics, Institute of Neuroscience, NHC and CAMS Key Laboratory of Medical Neurobiology, Zhejiang University School of Medicine, Hangzhou 310058, China
[5]Department of Head & Neck Surgery, Renji Hospital and Shanghai Jiao Tong University School of Medicine, Shanghai 200127, China
[6]Department of Otorhinolaryngology Head & Neck Surgery, Xinhua Hospital and Shanghai Jiao Tong University School of Medicine, Shanghai 200092, China
[7]Department of Physiology and Pathophysiology, School of Basic Sciences, Xinxiang Medical University, Xinxiang, Henan Province 453003, China
[8]School of Biomedical Sciences, Faculty of Biological Sciences, University of Leeds, Leeds LS2 9JT, UK
[9]These authors contributed equally
[10]Lead contact
*Correspondence: hbshi@sjtu.edu.cn (H.-B.S.), luyang.wang@utoronto.ca (L.-Y.W.), skyin@sjtu.edu.cn (S.-K.Y.)
https://doi.org/10.1016/j.neuron.2023.02.022

SUMMARY

Stroke prognosis is negatively associated with an elevation of serum bilirubin, but how bilirubin worsens outcomes remains mysterious. We report that post-, but not pre-, stroke bilirubin levels among inpatients scale with infarct volume. In mouse models, bilirubin increases neuronal excitability and ischemic infarct, whereas ischemic insults induce the release of endogenous bilirubin, all of which are attenuated by knockout of the TRPM2 channel or its antagonist A23. Independent of canonical TRPM2 intracellular agonists, bilirubin and its metabolic derivatives gate the channel opening, whereas A23 antagonizes it by binding to the same cavity. Knocking in a loss of binding point mutation for bilirubin, TRPM2-D1066A, effectively antagonizes ischemic neurotoxicity in mice. These findings suggest a vicious cycle of stroke injury in which initial ischemic insults trigger the release of endogenous bilirubin from injured cells, which potentially acts as a volume neurotransmitter to activate TRPM2 channels, aggravating Ca^{2+}-dependent brain injury.

INTRODUCTION

Stroke is one of the leading causes of death and the adult disability worldwide.[1] The most typical pathological cause of stroke is the interruption of cerebral blood perfusion due to atherosclerosis,[2] atrial fibrillation,[3] hypertension, diabetes,[4] etc. Stroke can be divided into ischemic stroke and hemorrhagic stroke, with the former being the most common, constituting around 80% of cerebral infarctions.[5] In ischemic stroke, circulating clots (or atherothrombosis) block the blood vessels in the brain (e.g., middle cerebral artery [MCA]), interrupting blood and oxygen supply and resulting in the necrosis of neurons and eventual damage of brain structure and function. During stroke, cerebral ischemia leads to a massive release of glutamate that activates NMDARs and induces Ca^{2+} influx through these ionotropic channels to overload neurons and destroy their Ca^{2+} homeostasis.[6] The Ca^{2+}-dependent activation of cell-death signaling immediately downstream of NMDARs triggers a plethora of cascades that work synergistically to induce neuronal death. These may activate other non-selective cation channels such as acid sensing ion channels (ASICs) and transient receptor potential melastatin (TRPM) channels.[7] Despite extensive work on ischemic damage, few drugs offering significant benefits in preclinical studies have successfully advanced to the stage of approved therapeutics for stroke patients, rationalizing the pressing need to identify the mechanisms of stroke and potential new targets for effective treatments of ischemic stroke.

Stroke appears to be associated with an elevation of bilirubin, which is the end product of heme catabolism in mammals. Bilirubin levels are typically low (<1 mg/dL) but when the

© 2023 The Author(s). Published by Elsevier Inc.
This is an open access article under the CC BY license (http://creativecommons.org/licenses/by/4.0/).

concentration exceeds certain levels (i.e., 3 mg/dL), it can be visually observed as hyperbilirubinemia (HB) or jaundice, at which point it can be considered a disease state.[8] Unconjugated bilirubin (UCB) is lipophilic and readily exchanges between the brain and periphery, particularly when the integrity of blood-brain barrier (BBB) is compromised in ischemic stroke. UCB can disrupt intracellular Ca^{2+} homeostasis and cause neuronal damage by oxidative stress of endoplasmic reticulum (ER), triggering inflammatory responses and apoptosis.[9–13] Conversely, anti-inflammatory treatments can improve survival rate and reduce neurodegeneration in HB animal models.[14,15] Interestingly, increased levels of bilirubin have been found in patients with acute ischemic stroke and is associated with the severity of stroke.[16] However, it is unknown whether this elevated level of bilirubin is a cause or consequence of stroke.

Despite all the pathological changes associated with HB, neither specific targets nor the mechanisms of bilirubin-induced neurotoxicity are known. Extensive work from our group and others in a variety of neurons all converge to the idea that bilirubin-induced hyperexcitation and Ca^{2+} overload are the leading causes of neuronal injury.[11,13,17,18] Although many ion channels are modulated by bilirubin to affect neuronal excitability and Ca^{2+} levels, it has barely been explored whether bilirubin can directly gate the opening of Ca^{2+} permeable ion channels from extracellular space and initiate the Ca^{2+}-dependent signaling cascade that precedes ultimate neuronal injury and death.

The TRPM2 channel is one of the several highly Ca^{2+} permeable non-selective cation channels and mediates a variety of physiological and pathological processes by regulating multiple intracellular signaling pathways, such as insulin secretion, inflammatory cell migration, and apoptosis.[19–21] These channels are expressed in immune cells, such as neutrophils,[22,23] macrophages,[24] monocytes,[25,26] and lymphocytes[27,28] and are deeply involved in their functions, including the release, chemotaxis, and recruitment of cytokines during the inflammatory response. TRPM2 channels are also abundantly expressed in the brain and serve as the receptor for non-painful warm stimulation in the peripheral nervous system.[29,30] TRPM2 is an oxidative stress sensor *in vivo* and can be activated by ADP-ribose (ADPR) and/or the oxidative stress generated by ADPR.[19,31,32] Canonically, NMDAR-dependent Ca^{2+} influx and ADPR rise lead to subsequent activation of multiple TRP channels including TRPM2. These pathways have been viewed as the primary routes for ischemia and reperfusion-induced cell death in stroke.[33,34] However, it remains unknown whether TRPM2 channels can independently serve as the receptors for endogenous agonists from the extracellular environment to activate downstream pathological processes in ischemic brain injury.

In this study, we explored the mechanisms by which bilirubin causes neuronal damage in mouse models and human subjects with stroke. We consistently found a strong correlation between post-stroke serum bilirubin levels and infarct volume in both patients diagnosed with stroke and rodent models. We found that genetic deletion of TRPM2 channels can mitigate bilirubin-induced hyperexcitability in mouse cortical neurons *in vitro* and reduce ischemia infarction in adult mice with transient middle cerebral occlusion (tMCAO) *in vivo*. By applying whole-cell and single-channel patch-clamp recordings from primary neurons and cell lines expressing wild type (WT)[35] and mutant TRPM2 channels lacking the canonical binding sites for intracellular agonists, ADPR and Ca^{2+}, we demonstrated that bilirubin and its structural derivatives can directly bind to the TRPM2 channel in a deep pocket near the Ca^{2+} binding sites and gate the opening of the channel. Supported by experiments and computational modeling of direct interactions between an agonist or antagonist with this cavity as well as the results from a transgenic mouse harboring a mutation in the cavity to prevent bilirubin binding, we propose a noncanonical paradigm, in which bilirubin is released from endogenous sources during ischemia and potentially serves as a volume neurotransmitter or a specific agonist ligand for TRPM2 to aggravate brain injury in stroke.

RESULTS

Abnormally elevated bilirubin aggravates the severity of stroke in patients

The exact effects of bilirubin are controversial. HB is well established as being harmful to the sensory, motor, and cognitive systems, particularly in the early developing brain. Conversely, bilirubin has also been implicated as an endogenous antioxidant that might be protective against diseases associated with oxidative stress, such as cardiovascular disease (CVD), and even cancer.[36,37] In the case of stroke, clinical data indicate that patients with high serum bilirubin levels seemed to manifest more severe neurological symptoms.[38,39] The NIH stroke scale (NIHSS) widely used in clinical studies is a valid indicator to measure the severity of stroke but is limited by the subjective nature of neurobehavioral indices and may not reflect the timing or extent of brain injury accurately. Magnetic resonance imaging (MRI) can identify the location and size of ischemia accurately and directly; so, we opted to use the MRI results of clinical stroke patients as an objective readout to analyze the relationship between bilirubin and infarct volume (Figure 1A). We collected and analyzed clinical records of adult stroke inpatients, which were divided into two groups based on their serum total bilirubin (TB) concentrations: normal and HB group (Figure S1A; Table S1). The positive diagnosis of HB was made in accordance with the criteria by a clinical biochemistry lab (TB > 18 μmol/L), independent of this study. Post hoc analyses of data from these two groups showed that stroke patients with HB had 2- to 3-fold higher levels of TB and direct bilirubin (DB) levels than the normal group (Figure 1B, left and middle panel). By quantitatively measuring infarct volume from MRI scan images among the same cohort of stroke patients, we found that the HB group showed a larger infarct volume than the normal (Normal: 18.45 ± 3.25 cm^3, HB: 76.42 ± 13.38 cm^3; $p < 0.001$; $n = 167$ vs. 47; Figure 1B, right panel). Similarly, the statistical difference remained robust even if the DB concentration was used to divide the two groups (Figure S1B). Both TB and DB levels were found to be strongly correlated with the infarct volumes by Spearman correlation analyses (Figure 1C). By comparing the bilirubin levels from serum tests from patients' medical history before and after stroke in the HB group, we also found that stroke significantly increased TB and DB concentration, suggesting that elevated bilirubin was likely a consequence of stroke (Figure 1D).

CellPress
OPEN ACCESS

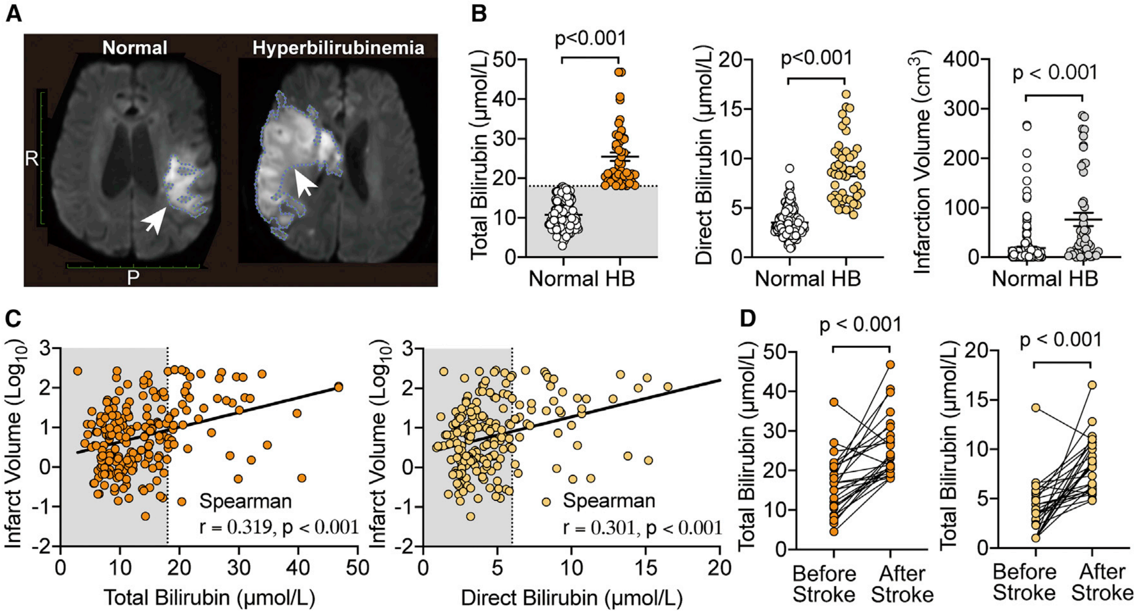

Figure 1. Serum bilirubin is elevated in stroke patients and correlated with infarct volume

(A) Representative MRI images (diffusion weighted imaging [DWI]) of stroke patients with normal (Normal group) and abnormal (HB group) serum bilirubin levels. The areas indicated by the white arrow are the infarcted brain tissue (2D schematic diagram).

(B) Summary data showing TB, DB concentration, and infarct volumes of stroke patients (Normal = 167, HB = 47).

(C) Scatter plots by Spearman correlation analysis showing relationships of the TB, DB levels, and infarct volume in stroke patients.

(D) Summary plots of serum TB and DB levels of serum tests from previous medical history and after stroke in enrolled patients with HB (n = 26).

Gray area represents the reference values for normal bilirubin by clinical biochemistry (TB $\leq$ 18 μmol/L, DB $\leq$ 6 μmol/L).

Error bars represent means ± SEM; unpaired Student's t test, paired Student's t test.

These results indicated that elevated bilirubin levels in human stroke subjects positively scale with the infarct volume.

TRPM2 channel mediates bilirubin-induced brain damage in ischemic mouse models

Previous studies have shown that the rise in intracellular Ca^{2+} concentration following ischemia leads to the overproduction of reactive oxygen species (ROS) that activates the downstream ion channels and/or cell-death signal pathways.[40] In tMCAO animal models, ROS overproduction predominates during the reperfusion process and can activate TRPM2 channels to exacerbate ischemia-reperfusion injury.[41] Given the known roles of TRPM2 in stroke, we first explored whether this channel was the substrate underlying the heightened effects of HB on infarct volumes. To this end, we examined brain injury 24 h after 30 min tMCAO in adult *Trpm2*$^{+/+}$ and *Trpm2*$^{-/-}$ mice from which the relationships between bilirubin and infarct volume were then compared. This relatively short period of tMCAO was deliberately chosen to test if bilirubin can aggravate ischemic infarction while avoiding the ceiling effect of longer tMCAO. Before and after tMCAO surgery, we monitored the blood pressure, body temperature, and serum biochemical indexes of the mice. Although occlusion of the MCA significantly reduced the blood flow of the brain by ~60%, these indexes were comparable (Figures S2A and 2B). Because mice exhibit relatively lower basal level and post-surgery elevation of bilirubin compared with human patients (likely due to species difference), we injected mice intraperitoneally with bilirubin (Bil group, 50 μg/g) or saline control (Ctrl group) 30 min prior to surgery. In *Trpm2*$^{+/+}$ mice with tMCAO, bilirubin increased the infarct volume (Ctrl: 34.17% ± 2.61%, Bil: 55.31% ± 3.68%; $p < 0.001$; n = 14). In contrast, the infarct volume in *Trpm2*$^{-/-}$ mice did not change by bilirubin injection (Ctrl: 23.58% ± 3.33%, Bil: 25.69% ± 4.46%; p = 0.35; n = 15, Figures 2A and 2B). Under our mild tMCAO paradigm, this difference appeared to be genotype specific but sex-independent (Figure S2C). The severity of brain damage in the Bil group remained significantly higher than that in the Ctrl group at day 7 after tMCAO (Figure S3).

Although the bilirubin levels in the cerebrospinal fluid (CSF) of the human stroke subjects were unknown and assumed to be positively correlated with its levels in the blood, we were able to measure the concentrations of bilirubin in both the blood and CSF from the same mice before their sacrifice and found that mice injected with bilirubin had markedly higher TB levels in both serum and CSF than those injected with saline. Both serum and CSF TB showed significant correlations with the infarct volume in *Trpm2*$^{+/+}$ animals (Figure 2C). Interestingly, despite similar increases of TB levels in the blood, neither the serum nor CSF bilirubin levels correlated with infarct volume in *Trpm2*$^{-/-}$ mice (Figure 2C). By using a highly sensitive fluorescent protein-based (i.e., UnaG) assay of bilirubin, we directly measured concentrations of UCB, which is the most relevant index for free bilirubin.[42–44] In *Trpm2*$^{+/+}$ mice, the UCB concentration of the Bil group was also much higher than that of the Ctrl group (both in

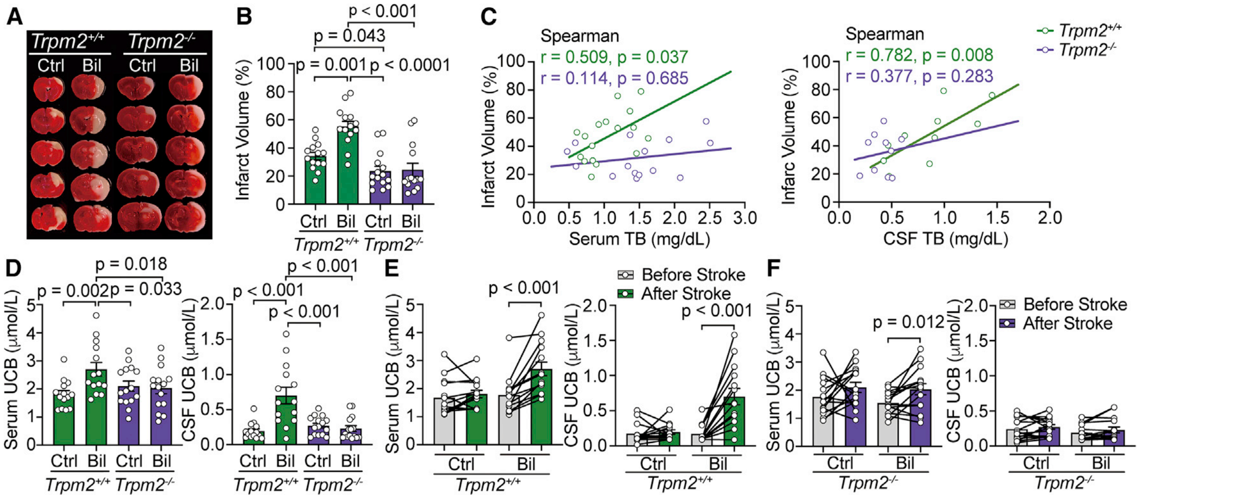

Figure 2. Hyperbilirubinemia exacerbates brain damage in adult ischemia models *in vivo* (24 h after tMCAO)

(A) Representative images of brain sections by 2,3,5-triphenyl-tetrazolium chloride (TTC) staining of tMCAO *Trpm2*$^{+/+}$ and *Trpm2*$^{-/-}$ adult mice with intraperitoneal injection of saline (Ctrl) and bilirubin (Bil).

(B) Summary data showing normalized infarct volumes of Ctrl and Bil group in *Trpm2*$^{+/+}$ and *Trpm2*$^{-/-}$ mice (n = 14 vs. 15).

(C) Spearman correlation analyses of serum/CSF TB concentration and the infarct volume in tMCAO mice (n = 10–17). In *Trpm2*$^{+/+}$ mice, both serum and CSF TB were significantly correlated with the infarct volume, but these correlation were absent in *Trpm2*$^{-/-}$ mice.

(D) Summary data showing serum and CSF UCB concentration of Ctrl and Bil group in *Trpm2*$^{+/+}$ and *Trpm2*$^{-/-}$ animals after tMCAO surgery (n = 14 vs. 15).

(E) Comparison of UCB concentration in serum and CSF of *Trpm2*$^{+/+}$ mice in the Ctrl and Bil group before and after ischemia-reperfusion injury (n = 14).

(F) Summery data showing UCB concentration in serum and CSF of *Trpm2*$^{-/-}$ mice in Ctrl and Bil group before and after ischemia-reperfusion injury (n = 15).

Error bars represent means ± SEM; paired Student's t test, one-way ANOVA with the post hoc LSD test.

serum and CSF) 24 h after tMCAO (Figure 2D), in line with the trend of TB. In contrast, the level of UCB in *Trpm2*$^{-/-}$ mice did not change, raising the possibility that ischemic injury elevated the release of endogenous bilirubin as observed in *Trpm2*$^{+/+}$ mice (Figure 2D). Indeed, when we compared the UCB concentrations in mice before tMCAO surgery, we found no differences in basal UCB concentration in both genotypes. However, a robust elevation of UCB in *Trpm2*$^{+/+}$, but not in *Trpm2*$^{-/-}$ mice, was observed 1 h after surgery, suggesting that ischemic injury alone can increase the level of bilirubin in CSF during the acute phase of reperfusion (Figures S4A–S4C). UCB concentrations in both *Trpm2*$^{+/+}$ and *Trpm2*$^{-/-}$ mice increased significantly during ischemic-reperfusion brain injury, reaching the peak at 3–6 h after surgery. The more drastic elevation of serum UCB levels in *Trpm2*$^{+/+}$ was particularly notable at 3 h (Figure S4D). In contrast, the serum UCB level in *Trpm2*$^{-/-}$ subsided 6 h after surgery. Pre-injection of bilirubin may have selectively boosted the level of UCB 24 h after tMCAO surgery at a higher level in *Trpm2*$^{+/+}$ than in *Trpm2*$^{-/-}$ mice, likely as a result of elevated local release of endogenous bilirubin due to greater infarct volume and/or red blood cell (RBC) extravasation (Figures 2E and 2F). However, we cannot exclude the possibility that the metabolism of bilirubin in the periphery and/or its transportation through the BBB may be regulated by TRPM2, indirectly contributing to genotype-specific differences in CSF UCB levels after tMCAO. These differences in ischemic infarct volume and bilirubin levels between *Trpm2*$^{+/+}$ and *Trpm2*$^{-/-}$ mice before and after tMCAO led us to conclude that ischemia-reperfusion during stroke aggravates brain injury and increases the concentration of bilirubin in some way that might involve TRPM2.

Bilirubin elevates the intrinsic excitability of cortical neurons in adult mice through the TRPM2 channel

Previous studies have shown that bilirubin can cause neuronal overexcitation and injury in a Ca^{2+}-dependent manner.[10,11,13,18] Combined with the extensive expression of TRPM2 in cortical regions and reduced ischemic injuries in *Trpm2*$^{-/-}$ mice, these channels can be rationalized as potential targets of bilirubin. To this end, we made whole-cell current-clamp recordings from slices to investigate the effects of bilirubin on the intrinsic excitability of layer 5 cortical pyramidal neurons from adult mice. Because bilirubin is known to affect neurotransmitter release,[45] we first eliminated excitatory and inhibitory inputs by perfusing a cocktail of synaptic blockers (50 μM D-(-)-2-Amino-5-phosphonopentanoic acid [APV], 10 μM 2,3-dihydroxy-6-nitro-7-sulfamoyl-benzo(f)quinoxaline [NBQX], 10 μM bicuculline, and 1 μM strychnine) and measured the intrinsic excitability by injecting a series of current steps (−200 to +400 pA, 50 pA increments). The number of evoked spikes by each current step was counted as a measure of the membrane excitability and plotted against the magnitude of current steps. As reflected by the input-output curves before and after administration of bilirubin in the same cells for 5 and 20 min, bilirubin significantly increased the excitability of cortical neurons in a time-dependent manner without affecting their resting membrane potential (V_{rest}) in slices from *Trpm2*$^{+/+}$ mice, but these effects were absent in slices from *Trpm2*$^{-/-}$ mice (Figures 3A–3C). Comparison of the spike waveform in cortical neurons evoked by a brief current pulse (2.5 nA current in 0.3 ms from the set membrane potential of −64 mV) showed the half-width did not change but the spike amplitude was significantly attenuated in both groups 20 min after bilirubin

CellPress
OPEN ACCESS

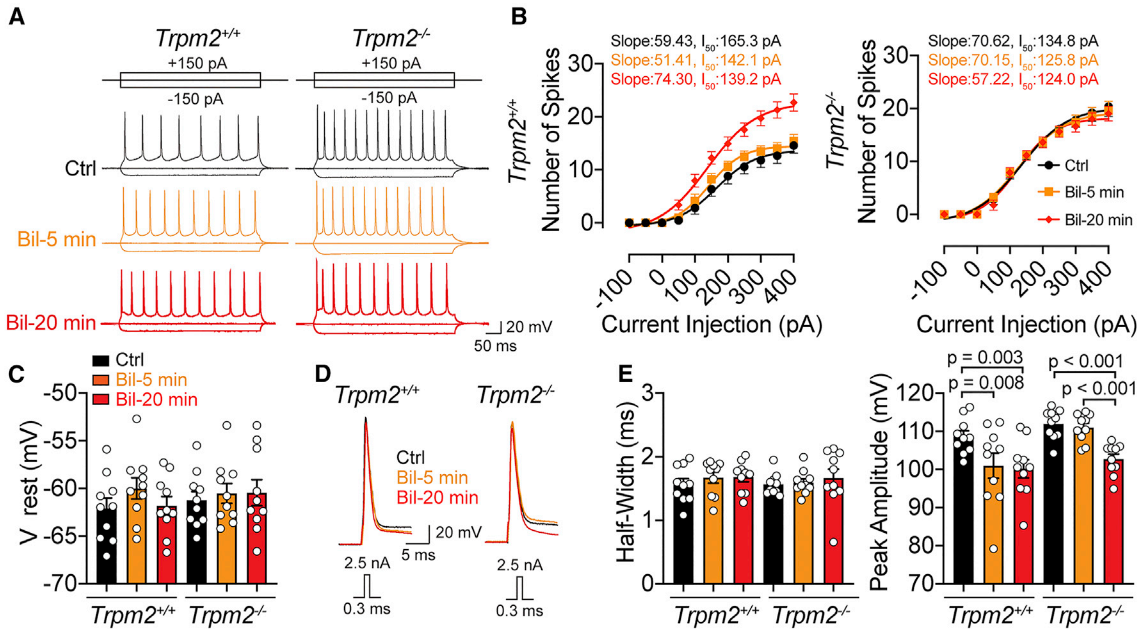

Figure 3. Prolonged application of bilirubin induces neuronal hyperexcitation *in vitro*

(A) Example traces of action potentials (APs) evoked by current injections before and after bilirubin perfusion for 5 and 20 min of cortical neurons from adult *Trpm2*$^{+/+}$ and *Trpm2*$^{-/-}$ mice.

(B) The mean number of spikes induced by depolarization steps in 50 pA increments to cortical neurons of *Trpm2*$^{+/+}$ and *Trpm2*$^{-/-}$ mice (n = 9). Data were fitted with the Boltzmann function, showing an increase in the slope factor and a reduction in the current magnitude to evoke 50% of the maximal spikes in *Trpm2*$^{+/+}$ but not *Trpm2*$^{-/-}$ neurons after application of bilirubin for 20 min.

(C) Summary plots of the resting membrane potential (Vrest), showing no significant differences between each group (n = 10).

(D) Representative recordings of action potential evoked by 2.5 nA current injection in 0.3 ms were compared before and after bilirubin perfusion in *Trpm2*$^{+/+}$ and *Trpm2*$^{-/-}$ cortical neurons.

(E and F) Summary plots of the peak-amplitude and half-width from the cell in (D) (n = 10).

Error bars represent means ± SEM; one-way ANOVA with the post hoc LSD test.

application, likely due to other effects of bilirubin that were not exclusively mediated by TRPM2 (Figures 3D and 3E). Notably, the basal intrinsic excitability of cortical neurons in *Trpm2*$^{-/-}$ mice was slightly elevated, possibly due to compensation after *Trpm2* knockout (Figure 3B). In short, deletion of TRPM2 channels prevented bilirubin from exerting its enhancing effects on the intrinsic excitability, implicating TRPM2 channels as the primary target (or receptor) for bilirubin.

Bilirubin activates TRPM2 currents by directly binding to the channel

The TRPM2 channel is classically known for being activated by ROS and requires the binding of both intracellular Ca^{2+} and ADPR to reach the open state. Despite being an antioxidant itself with the capacity to neutralize ROS, bilirubin evidently elevated the neuronal excitability and aggravated neuronal death as shown in aforementioned experiments *in vitro* and *in vivo*. These intriguing observations led us to hypothesize that bilirubin might act in some unknown way (directly or indirectly) to gate the opening of the TRPM2 channel. To explore this, we used HEK293 cells expressing the human TRPM2 (hTRPM2) channel in a tetracycline-inducible manner (HEK-hTRPM2). We found that bilirubin (9 μM) increased the magnitude of the TRPM2 currents evoked by voltage ramps (−100 to +100 mV), as shown by time-dependent increases in current amplitude at both negative and positive potentials without affecting the reversal potential (~0 mV) of the current-voltage (I-V) curves (Figure 4A, upper). To distinguish whether bilirubin activated the TRPM2 channel directly or indirectly via elevating ADPR production, we used PJ-34 to block poly (ADPR) polymerase (PARP), the key enzyme for ROS-induced ADPR generation,[46] and found that bilirubin remained effective in activating TRPM2 currents (Figure 4A, bottom). Bilirubin increased TRPM2 currents when the intracellular solution contained a subthreshold concentration of ADPR (5 μM) but failed to do so when the intracellular concentration of ADPR was increased to 500 μM, a saturated concentration for TRPM2 channel activation (Figure 4B, upper and middle). These results suggested that bilirubin and ADPR activate the same population of TRPM2 channels.

The activation of TRPM2 channels by ADPR depends on the presence of intracellular Ca^{2+}.[47] However, we found that bilirubin remained capable of activating the TRPM2 channel even when fast-acting Ca^{2+} buffer 1,2-bis-(2-aminophenoxyethane) ethane-N,N, N′, N′-tetraacetic acid **(**BAPTA) (30 mM) was used to replace EGTA to chelate intracellular Ca^{2+}, though the current amplitude was noticeably reduced (Figure 4B, bottom). Figure 4C summarizes the pooled results on bilirubin-induced changes in

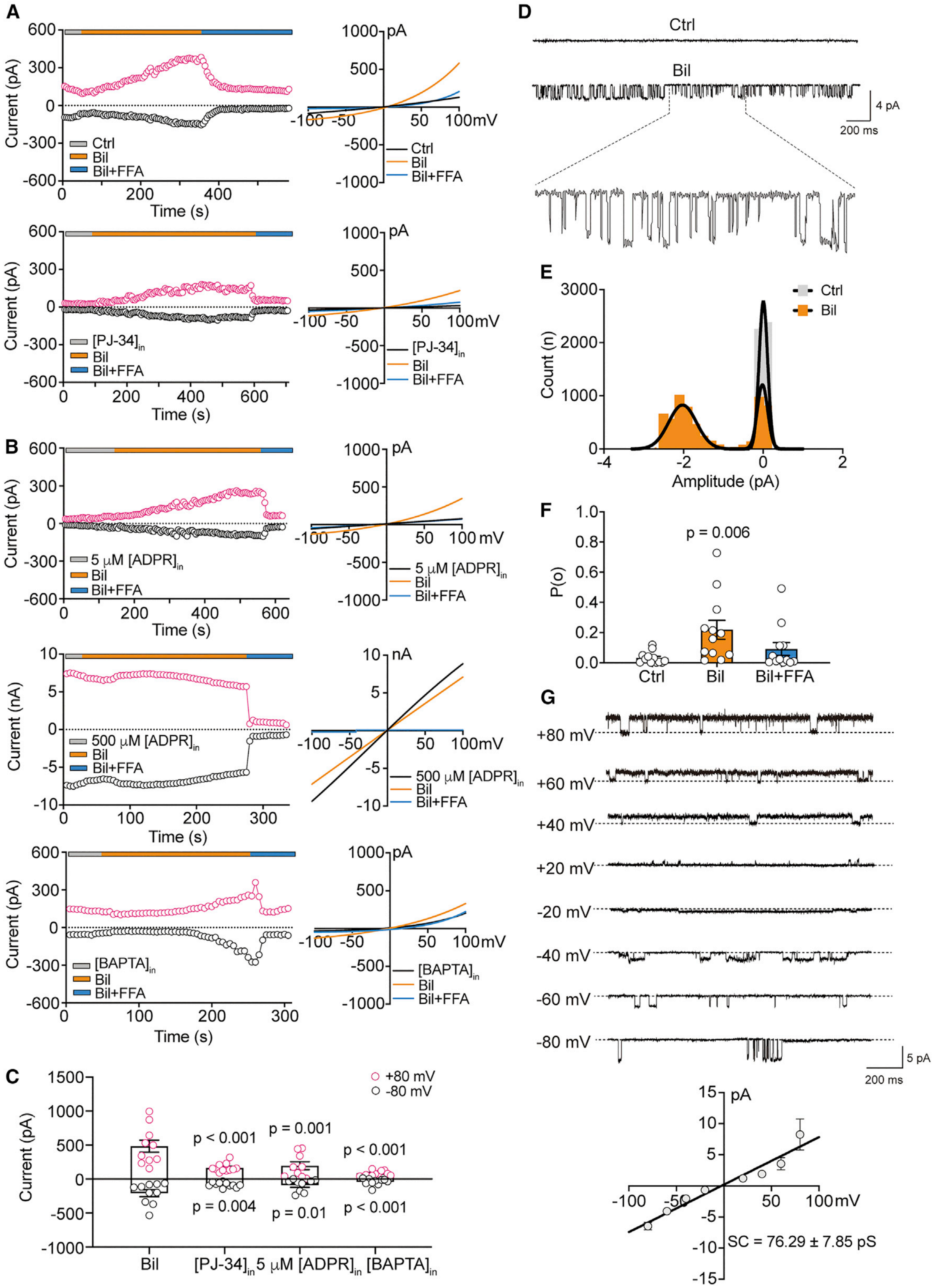

Figure 4. Bilirubin activates TRPM2 currents independent of intracellular ADPR

(A and B) Representative time course of the currents activated by voltage ramps from −100 to +100 mV (500 ms) and the amplitudes measured at +80 mV (strawberry circle) and −80 mV (black circle) are plotted against time after membrane breakthrough. Example ramp current traces before and after bilirubin

(legend continued on next page)

the amplitude of TRPM2 currents under these conditions. Furthermore, we examined that the effect of bilirubin in HEK293 cells, transiently expressing hTRPM2 channels with mutations, is known to disable binding sites for Ca^{2+} and found that it did not prevent the activation of TRPM2 currents by bilirubin (Figure S5), despite some differences in the magnitude of TRPM2 currents under these experimental conditions. Overall, these results supported the notion that bilirubin can activate TRPM2 channels independently of the canonical channel activators, namely ADPR and Ca^{2+}.

To provide direct evidence for TRPM2 channel activation by bilirubin, we performed single-channel current recordings in the outside-out configuration, under which pipette solution dialysis was expected to remove any residual ADPR that remained present in whole-cell recordings. We found that bilirubin (300 nM) elicited single-channel openings in membrane patches from induced HEK-hTRPM2 cells: these channels exhibited biophysical properties and pharmacological sensitivity to the blocker flufenamic acid (FFA) characteristic of the TRPM2 channel (Figures 4D–4G). As shown in Figure 4E, the all-point histograms of the current amplitudes before and after perfusing bilirubin revealed that it generated single-channel currents of −2.02 ± 0.05 pA at −40 mV. Further analyses of the dwell times in open and closed states showed that the opening probability (Po) was significantly increased by bilirubin, which was reversed by FFA (Figure 4F). By plotting the amplitude of single-channel currents at different holding potentials (Figure 4G), we derived a slope conductance of 76.29 ± 7.85 pS from the linear fit to their bilirubin-driven current-voltage relationship, which is identical to that of its intracellular agonist ADPR (Figure S6) and consistent with previously reported values (i.e., 60–74 pS).[48–51] Taken together, these results strongly support the notion that bilirubin directly gates the TRPM2 channel.

Derivatives of bilirubin activate the TRPM2 channel

Bilirubin is an endogenous substance that normally exists *in vivo*. When its concentration is abnormally high, the levels of related metabolic derivatives also increase.[52–54] We explored whether these derivatives with a similar structure can activate the TRPM2 channel. The chemical structures of bilirubin and three selected derivatives are shown in Figure 5A. Biliverdin, a linear tetrapyrrole intermediate, is degraded from heme and rapidly reduced by biliverdin reductase (BVR) to bilirubin. As showed in Figure 5B, biliverdin hydrochloride (Bild, 9 μM) induced TRPM2 currents in HEK-hTRPM2 cells, with comparable amplitudes to those induced by bilirubin. Bilirubin conjugate, ditaurate, disodium salt (BDS), is a water-soluble analog but is not membrane permeable. Similar effects to bilirubin application were observed in extracellular perfusion of BDS (9 μM) (Figure 5C), implying that the TRPM2 binding site is accessible from the outside. Finally, we found that xanthobilirubic acid methyl ester (XAME, 9 μM), a much smaller molecule that retains only two pyrrole backbones of bilirubin, was the most powerful activator of TRPM2 channels, not only showing shorter activation latency, but also generating a much greater current amplitude (Figure 5D). The effects of bilirubin and these three derivatives on the amplitude of the activated TRPM2 currents are summarized in Figure 5E. In fact, when the dose-dependent curve was constructed for XAME, we found that it had a much higher potency than bilirubin (Figure 5F), implying that XAME preserved the minimum structure required to be a direct agonist for TRPM2 channels and likely fit more readily than bilirubin into the same binding pocket to activate TRPM2 channels due to the smaller size of the molecule. These results raised the possibility that bilirubin and its metabolic derivatives associated with HB can all act as agonists to activate TRPM2 channels and exacerbate brain damage under ischemia conditions.

Bilirubin activates the TRPM2 channel through a new gating mechanism

Because both bilirubin and its structural derivatives directly activated TRPM2 channels, we postulated that there must be a common binding site such that these substances can access the TRPM2 channel from the extracellular side. Recent structural studies have established that the endogenous agonist ADPR binds to two intracellular sites, mediated by the N-terminal MHR1/2 and the C-terminal NUDT9-H domains, respectively, and cooperates with Ca^{2+} binding to their sites in the transmembrane domain to control the opening of the TRPM2 channel.[55] Because bilirubin is highly hydrophobic and membrane permeable, it was necessary to rule in or out the possibility that bilirubin binds to the ADPR-binding pockets to exert its agonist effects. We introduced point mutations at the N-terminal ADPR-binding site (R302A/R358A) and the C-terminal NUDT9-H domain (R1433A), which are known to block the binding of ADPR. We found that these mutant TRPM2 channels remained responsive to bilirubin, albeit with a reduced magnitude of activation (Figures 6A and 6B). In contrast, ADPR failed to evoke currents from R302A/R358A mutants (Figure S7). ADPR binding is coupled to the conformational change of the pore region, where specific mutations in the pore (i.e., top: P983A and D987A; bottom: A1046C and N1049C) (Figure S8) can prevent the pore opening and ion conduction of TRPM2 channels. Strikingly, bilirubin also effectively activated these pore mutants. To reinforce these

application and co-application with FFA were transformed into I-V relationships and overlaid on the right panels (in this and subsequent figures), showing TRPM2 currents activated by bilirubin in control solution and in the presence of PJ-34 (10 μM), ADPR (5 μM), ADPR (500 μM), and BAPTA (30 mM) in hTRPM2 transfected HEK-293T cells.

(C) Summary data showing the maximum amplitude of currents activated by bilirubin under each of the above conditions (n = 9–11).

(D) Representative single-channel recording showing bilirubin (300 nM) directly activated TRPM2 currents (holding potential −40 mV).

(E) The representative all-points amplitude histogram of single-channel current amplitude (0.2 pA/bin) before and after perfusing bilirubin. Their distributions were it by single or double-component Gaussian functions with means of −0.04 pA (gray, control) and −0.02 and −2.02 pA (yellow, before and after bilirubin).

(F) Statistical results of opening probability (Po) when perfusion of bilirubin and FFA (n = 12).

(G) Mean single-channel current amplitudes activated by bilirubin from −80 to +80 mV are plotted against each voltage and fit with linear regression to yield single-channel conductance of 76.29 ± 7.85 pS (n = 4–7).

Error bars represent means ± SEM; unpaired Student's t test, paired Student's t test.

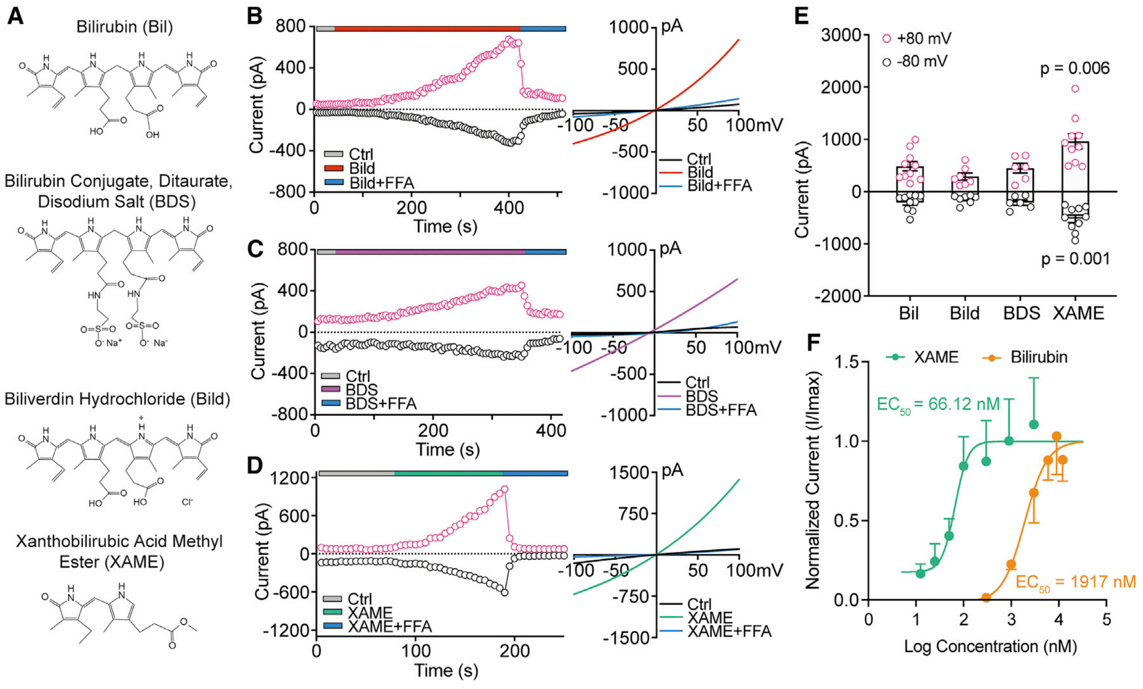

Figure 5. TRPM2 currents activated by bilirubin derivatives

(A) Chemical structure of bilirubin and the derivatives.

(B–D) Representative time course and I-V curves of currents at +80 mV (strawberry circle) and −80 mV (black circle) before and after Bild (9 μM), BDS (9 μM), or XAME (9 μM), respectively.

(E) Summary data showing the amplitude of maximum currents activated by bilirubin and its derivatives at +80 and −80 mV (n = 6–10).

(F) Concentration-response relationships for currents evoked by bilirubin and XAME (n = 4) and fit with the Hill equation (bilirubin: EC_{50} = 1,917 nM, Hillslope = 1.0; XAME: EC_{50} = 66.12 nM, Hillslope = 0.77).

Error bars represent means ± SEM; unpaired Student's t test.

observations, we made recordings from cells expressing another TRPM2 mutant, in which both N- and C-terminal ADPR-binding domains were truncated (TRPM2-ΔN/ΔC, with N terminus residues 535–555 and C terminus residues 1,291–1,329).[56] Such truncations eliminated the activation of the TRPM2 channel by ADPR but not by bilirubin (Figures 6C and 6D). These findings demonstrate that bilirubin gates the opening of the TRPM2 channel differently from intracellular activators.

The observations that bilirubin and its derivatives, particularly with BDS being membrane impermeable, all had relatively long time courses for activation of TRPM2 channels, and that channel activation by a smaller molecule, such as XAME, was stronger and faster, implied that the binding pocket could be accessed from the extracellular space. However, such a binding pocket must be located rather deep into the TRPM2 channel complex with physical hindrances present that would slow the access and binding of bilirubin. In search of the binding pocket, we performed molecular docking *in silico* of bilirubin onto the extracellular and transmembrane parts of the TRPM2 channels in the inactive (apo) and closed states versus ADPR and/or Ca^{2+} bound open states. The possible conformations of the TRPM2-bilirubin complex were rank-ordered by final docking scores from calculations of internal space constraints and molecular energetics of chemical bonds between different chemical moieties of bilirubin and the amino acid side chains of the TRPM2 protein (Figures 6E and 6F; Table S2). We found an optimal cavity near the Ca^{2+} binding sites, where bilirubin was surrounded by the S3, S5, and TRP helix (Figure 6F, left panel). Amino- and carboxyl moieties of bilirubin make four hydrogen bonds (H-bonds) with D866, W868 in S3, and D1069 in the TRP helix and two additional salt bridges with K928 in S5, whereas its pyrrole moiety forms a strong π-π interaction with W868 (Figure 6F, right panel).

Among these hypothetical interaction sites, K928 and D1069 represent the strongest binding sites based on bond energy analysis. To gain further insights into the ligand-channel interactions as predicted by *in silico* modeling, we used site-directed mutagenesis to replace the positively charged lysine at 928 and negatively charged aspartate at 1069 with nonpolar alanine. We found that both intracellular ADPR and Ca^{2+} perfectly retained their agonist capacity to activate this K928A/D1069A double mutant TRPM2 channel with the current amplitude being indistinguishable from that of the WT hTRPM2 channel (Figures 6G–6I). These indicated that the double mutations did not perturb the gating and permeation properties of TRPM2 channels via canonical intracellular agonists. In contrast, bilirubin and its derivatives completely lost its ability to activate this double mutant (Figures 6J and 6K), except for XAME, which induced very small currents (Figure S9). Molecular docking simulations showed that the binding pocket of XAME is not identical to that of bilirubin, engaging partially overlapping amino acid residues

CellPress
OPEN ACCESS

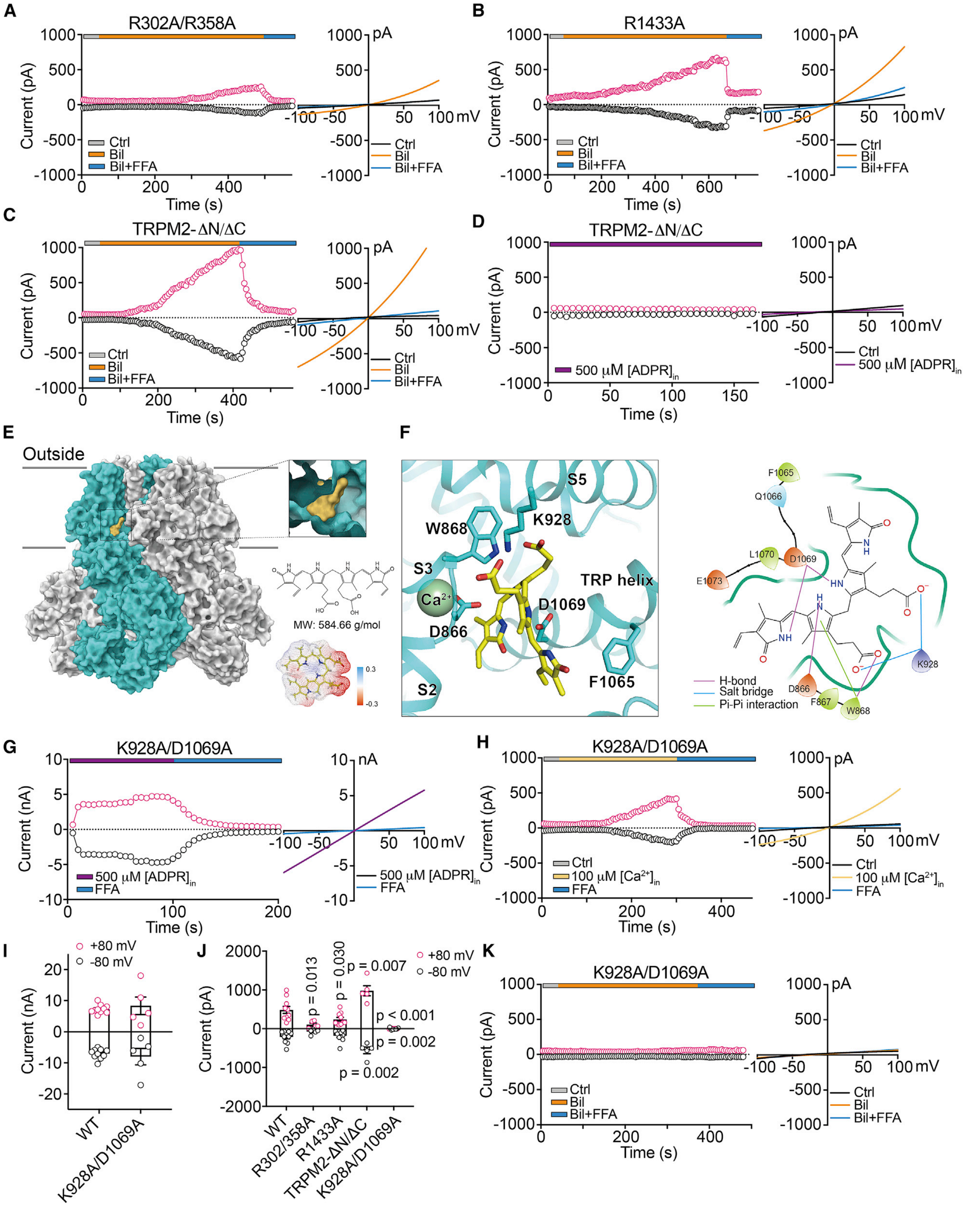

(legend on next page)

in the pocket (Figures S10A and 10B). Further analysis of their binding pockets showed that either ligand can be perfectly accommodated at equilibrium (Table S3).

Given the difference in the binding affinity and activation time course between XAME and bilirubin, we subsequently performed molecular dynamics (MD) simulations to model the state transition of the ligand-TRPM2 complex embedded in the phospholipid bilayer (Figure S10C). Statistical analysis of root mean square deviation (RMSD) for the first 50 ns upon the formation of the channel-ligand complex showed that both ligands can associate with TRPM2 into stable complexes. XAME appeared to approach a stable association within the first 10 ns, faster than bilirubin, which continued to display large fluctuations in binding and unbinding throughout the 50 ns bouts of simulation, likely because of the lower polarity and greater flexibility of XAME compared with bilirubin (Figures S10D and 10E). Molecular mechanics-Poisson Bolzmann surface area (MM-PBSA) calculations further validated that XAME has a higher affinity for the TRPM2 channel than bilirubin (Figure S10F). The binding energies contributed by different residues in the binding cavity indicated that the main residues that stabilized bilirubin binding to the TRPM2 channel are D866, K928, and D1069, which partially overlap with or are in the vicinity of the residues that interact strongly with XAME (Figure S10G). These compelling results demonstrated that bilirubin activates the TRPM2 channel via a different gating mechanism from canonical intracellular ligands.

The bilirubin-binding cavity in the TRPM2 channel is an ideal druggable site for antagonizing neurotoxicity

TRPM2 has been well established as a target for stroke therapy with several structurally distinct blockers, such as clotrimazole, tatM2NX, and A23, considered neuroprotective in preclinical studies with animal models.[57–59] Using the identified binding pocket for bilirubin as a 3D docking template, we performed extensive *in silico* screening of these known compounds. We found A23, a newly published TRPM2 blocker, formed a highly stable association with the cavity, thus providing a structural explanation for its nanomolar half-maximal inhibitory concentration *in vitro* and effectiveness against ischemic injury *in vivo*.[59] As illustrated in Figures 7A and 7B, unlike BDS, an agonist that required multidimensional interactions with the K725, L862, N869, K870, K928, and K932 residues through H-bonds and salt bridges, A23 formed H-bonds and salt bridges with K928 to optimally fit into the cavity. It exhibited the lowest docking score for the Apo State (6PUO) of TRPM2 (bilirubin −5.04, BDS −4.47, and A23 −8.57) and, therefore, the strongest binding. Patch-clamp experiments reinforced these simulation results. To avoid any off-target effects of bilirubin on intracellular gating pathways, we used its water-soluble and membrane-impermeable derivative BDS as an agonist to activate the TRPM2 channel (Figure 7C). A23 not only completely suppressed TRPM2 currents preactivated by BDS but also prevented its activation when A23 was first applied (Figure 7D), demonstrating its high affinity. Further experiments showed that A23 blocked currents activated by BDS, bilirubin, and ADPR with their IC_{50} being 0.72, 0.92, and 0.83 μM for each ligand, respectively (Figure 7E). Given its high affinity for the bilirubin-binding cavity in TRPM2, we next tested if A23 can attenuate bilirubin-driven neurotoxicity with an *in vitro* cell-death assay. We used calcein-am and propidium-iodide (PI) to label surviving and dead cortical neurons, respectively (Figure 7F). In adult *Trpm2*$^{+/+}$ brain slices, bilirubin significantly increased the mortality of cortical neurons after 1 h of incubation, and A23 fully antagonized this effect of bilirubin (Figure 7G Ctrl: 35.01% ± 1.49%, n = 6; Bil: 60.84% ± 2.13%, n = 6; Bil+A23: 35.27% ± 1.85%, n = 11).

Although we demonstrated that tMCAO paired with bilirubin injection leads to an increase in infract volume and elevated levels of both serum and CSF bilirubin (Figures 2A, 2B, and S4D), it remained unknown whether bilirubin can originate from the brain itself. To address this question, we first performed immunofluorescence staining to investigate the co-localization of the TRPM2 channel and key metabolic enzymes BVR and heme oxygenase-1 (HO-1) upstream of bilirubin. We found that both BVR and HO-1 were exclusively expressed in mouse neurons (i.e., not astrocytes) as shown by their strong co-staining with the neuronal marker (NeuN) in the brain of *Trpm2*$^{+/+}$and *Trpm2*$^{-/-}$ mice (Figures 7H and S11). These observations suggested that bilirubin must be primarily produced in and released from neurons during stroke. To simulate the pathological environment of ischemia and hypoxia, we performed oxygen-glucose-deprivation (OGD) experiments on acutely isolated brain slices and found that OGD increased the UCB concentration much more drastically in the supernatant of brain tissue from

Figure 6. Bilirubin activates the TRPM2 channel by binding to a cavity in the transmembrane region near the Ca^{2+} binding site

(A–C) Representative time course of currents at +80 mV (strawberry circle) and −80 mV (black circle) and superimposed I-V curves before and after bilirubin application and FFA co-application from N-terminal ADPR-binding site mutants (R302A/R358A), the C-terminal NUDT9-H domain mutants (R1433A), and truncation mutant (TRPM2-ΔN/ΔC).

(D) Representative examples of time course and I-V curves of currents activated by 500 μM ADPR in TRPM2-ΔN/ΔC.

(E) Molecular docking showing an overview of bilirubin-bound hTRPM2. A close-up view of the bilirubin-binding site is shown in the inset. Individual TRPM2 subunits are colored cyan and gray whereas bilirubin is highlighted with yellow, respectively. Chemical structure and the EM density of bilirubin are shown.

(F) Specific recognition of bilirubin-bound hTRPM2. 3D diagram showing that bilirubin binds a deep cavity of TRPM2 between S3, S5 and TRP helix, Ca^{2+} is colored green (left panel). 2D diagram showing that bilirubin interacts with TRPM2 through a mixture of hydrogen bonds (H-bonds), salt bridges, and π-π interaction (right panel).

(G and H) Representative time course of currents at +80 mV (strawberry circle) and −80 mV (black circle) activated by 500 μM ADPR and 100 μM Ca^{2+} in K928A/D1069A double mutant. I-V plots are shown in the right panels.

(I) Summary plot showing the amplitude of currents activated by ADPR in the WT TRPM2 channel and K928A/D1069A double mutant (n = 5–10).

(J) Summary data showing the amplitude of currents activated by bilirubin in WT TRPM2 channel and all mutants in (A)–(C) and (H) (n = 5–10).

(K) Representative time course of currents at +80 mV (strawberry circle) and −80 mV (black circle) activated by 9 μM bilirubin in K928A/D1069A double mutant. I-V plots are shown in the right panels. Error bars represent means ± SEM; unpaired Student's t test.

CellPress
OPEN ACCESS

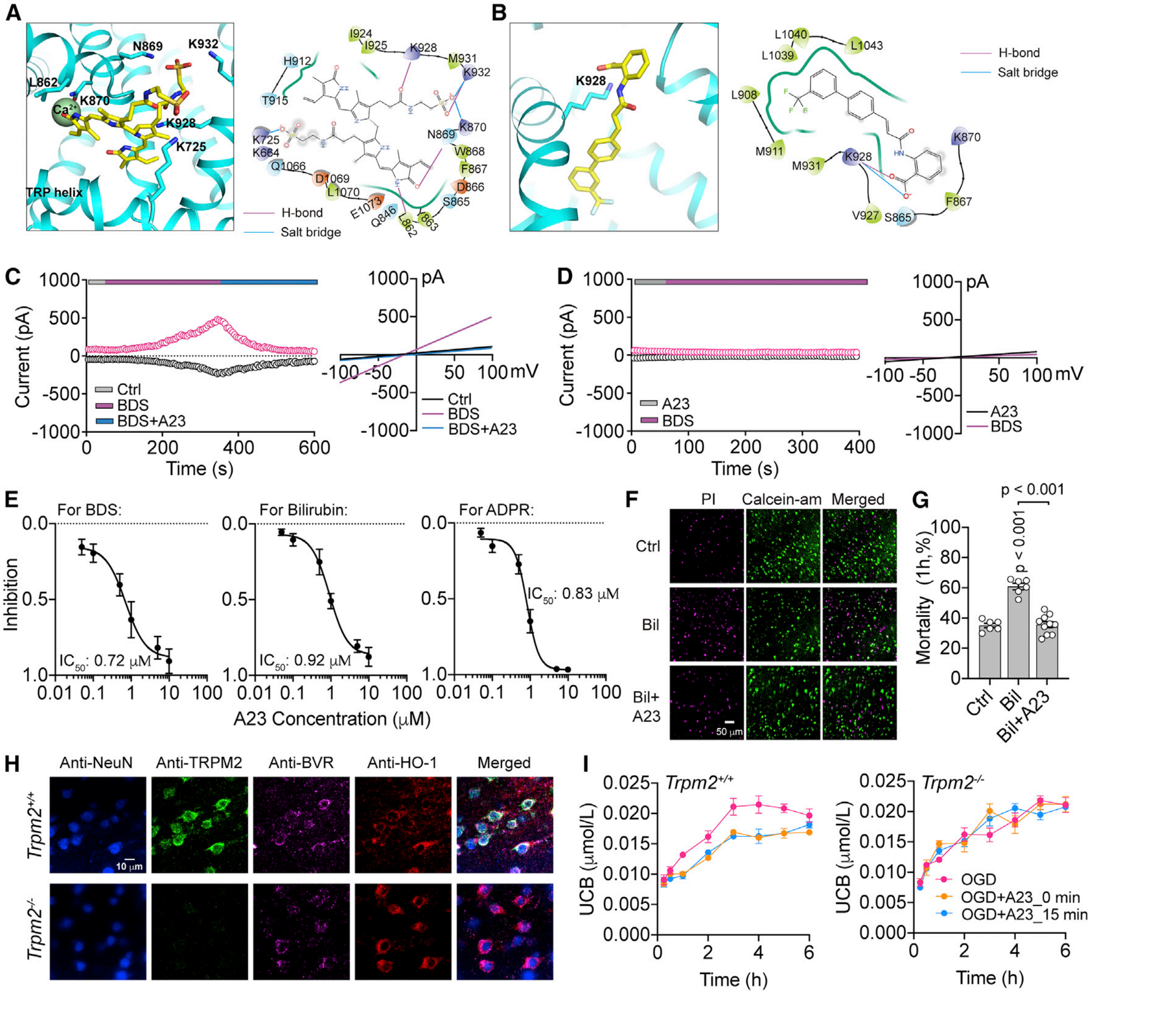

Figure 7. A23 antagonized the damage of bilirubin by specifically blocking the TRPM2 channel

(A and B) Molecular docking showing the specific recognition of BDS (A) and A23 (B)-bound hTRPM2. 3D diagrams showing that BDS and A23 docking to the cavity where bilirubin binds the TRPM2 channel. 2D diagrams showing BDS and A23 interacts with TRPM2 through H-bonds and salt bridges.

(C) Representative time course of TRPM2 currents activated by BDS, which was completely antagonized by A23. The I-V plot is shown on the right.

(D) When HEK293 cells incubated with A23 for 20 min in advance, BDS failed to activate the TRPM2 channel.

(E) Dose-response curves for A23 to block TRPM2 currents evoked by BDS, bilirubin, or ADPR, respectively. The concentration for A23 to produce 50% blockade (IC_{50}) were estimated to be 0.72, 0.92, and 0.83 μM from fits with the Hill equation.

(F) Representative immunofluorescence staining results of bilirubin-induced death of cortical neurons in adult *Trpm2*$^{+/+}$ brain slices. Prominent calcein-am staining (green) of nuclei marked the population of live neurons and PI staining (purple) of nuclei marked the population of dead neurons.

(G) Summary data showing mortality ratio of cortical neurons in (G) (n = 6–11).

(H) Immunofluorescence co-localization imaging of neuronal (anti-NeuN), TRPM2 channels (anti-TRPM2), biliverdin reductase (anti-BVR), and heme oxygenase 1 (anti-HO-1).

(I) Summary data showing the time course of OGD induced changes in the UCB concentration in brain slices from *Trpm2*$^{+/+}$ and *Trpm2*$^{-/-}$ mice under different conditions (n = 3).

Error bars represent means ± SEM; one-way ANOVA with the post hoc LSD test.

Trpm2$^{+/+}$ mice than from *Trpm2*$^{-/-}$ mice over the period of 6 h. This increase in *Trpm2*$^{+/+}$ was blocked by application of A23 either at the onset of OGD or 15 min after. In contrast, A23 had no effect in *Trpm2*$^{-/-}$ mice (Figure 7I). These results indicated that ischemic insults can directly induce the release of endogenous bilirubin from the brain to exacerbate neurotoxicity in stroke.

Molecular perturbation of the bilirubin-binding cavity on TRPM2 abolishes bilirubin-induced upregulation of excitability and neurotoxicity

Our experimental and simulation results converge to the conclusion that bilirubin and its structurally similar derivatives can activate TRPM2 channels by binding to a cavity near K928/D1069 where A23 exerts a competitive antagonism to block the binding

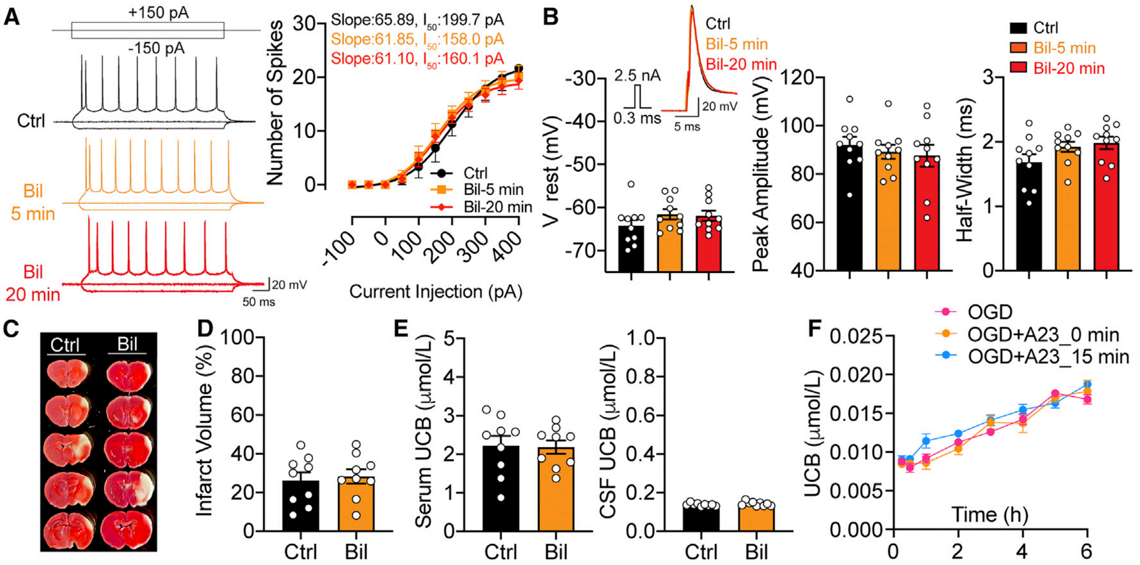

Figure 8. *D1066A* knockin (KI) mice phenocopy TRPM2 knockout mice in attenuating bilirubin-dependent effects on excitability and neurotoxicity *in vitro* and *in vivo*

(A) Example traces of action potentials (APs) evoked by current injections before and after bilirubin perfusion for 5 and 20 min of cortical neurons from *D1066A* KI mice. The number of spikes induced by various depolarization steps in 50 pA increments to cortical neurons were counted, averaged, and fitted with the Boltzmann function to show an ablation of bilirubin-dependent upregulation of the intrinsic excitability.

(B) Representative recordings of action potential evoked by 2.5 nA current injection in 0.3 ms were compared before and after bilirubin perfusion in *D1066A* KI cortical neurons. Summary plots of the resting membrane potential (Vrest), peak-amplitude, and half-width showing no significant differences between each group (n = 10).

(C) Representative images of brain sections by TTC staining of *D1066A* KI mice adult with intraperitoneal injection of saline (Ctrl) and bilirubin (Bil).

(D) Summary data showing little difference in normalized infarct volumes of the Ctrl and Bil group in *D1066A* KI mice (n = 9).

(E) Summary data showing comparable serum and CSF UCB concentration of the Ctrl and Bil group in *D1066A* KI mice 24 h after tMCAO surgery (n = 9).

(F) Summary data showing the time course of OGD induced changes in UCB concentration from *D1066A* KI brain slice under different conditions (n = 3).

Error bars represent means ± SEM; unpaired Student's t test, one-way ANOVA with post hoc LSD test.

of these agonists. To acquire unequivocal evidence that this cavity in the TRPM2 enables the binding of bilirubin to mediate its neurotoxicity, we made a knockin (KI) mouse line in which aspartic acid at the site 1066 of the TRPM2 (i.e., the analogous D1069 site of the human TRPM2) was replaced by a neutral amino acid residue alanine (D1066A). Our simulations and patch-clamp experiments in our cell line showed that this site is of critical importance for bilirubin, but not Ca^{2+}/ADPR, to function as a potent agonist for the TRPM2 channel. The KI mouse line thus serves as an ideal model to differentiate bilirubin-dependent and -independent actions of TRPM2. The *D1066A* mutant mice were viable and showed no obvious anatomical or behavioral abnormalities. Electrophysiological experiments revealed that in *D1066A* mice, bilirubin did not induce the hyperexcitability in cortical pyramidal neurons seen in WT even over a prolonged perfusion time (Figure 8A), nor did it cause any significant changes in the spike waveform (Figure 8B). Strikingly, exacerbated ischemic brain injury by bilirubin in the tMCAO model was completely ablated in *D1066A* KI mice, as was the increase in the UCB concentration before and after tMCAO surgery (Figures 8C–8E). *In vitro* OGD experiments on *D1066A* brain slices showed that A23 no longer had any effect on the release of endogenous bilirubin (Figure 8F). Because the TRPM2 channel with D1066A mutation remains functionally intact (with other intracellular agonists), *D1066A* KI mice mirror TRPM2 KO mice in attenuating bilirubin-induced upregulation of excitability and infarct volume, leading us to the conclusion that the binding cavity in the TRPM2 channel is indispensable for bilirubin to exert its actions in stroke.

DISCUSSION

Bilirubin is susceptible to deposition in brain tissue and results in damage to a subset of highly sensitive brain regions.[60] An increasing number of studies have shown that the bilirubin neurotoxicity is related to the disruption of the oxidative stress state of neurons and the increased production of oxidative stress products caused by bilirubin.[9,12,61,62] Gurses et al.[63] showed that HB affects the electrical activity in the cerebral cortex of human neonates. Our previous studies demonstrated that bilirubin damages mouse sensory neurons by affecting the function and/or level of ion channels and excitatory neurotransmission to cause neuronal overexcitation,[10,11,18,64] and by causing Ca^{2+} overload due to Ca^{2+} influx via voltage-gated calcium channels (VGCCs, P/Q-type in particular) and mobilization from internal Ca^{2+} stores. However, it remains ambiguous whether bilirubin exerts its multifaceted actions by binding to a broad spectrum of subcellular substrates or one specific receptor to cause neuronal overexcitation, Ca^{2+} overload and cell death. In this work, we demonstrated that bilirubin binds directly to a specific cavity of TRPM2 channels

CellPress
OPEN ACCESS

to gate channel opening and that knockout or blockade of TRPM2 channels and KI of TRPM2 *D1066A* devoid of the bilirubin-binding site can all antagonize bilirubin-induced hyperexcitation *in vitro* and attenuate neurotoxicity *in vivo*. These results provided compelling evidence that the TRPM2 channel serves as the core receptor on the plasma membrane of central neurons to sense bilirubin or its metabolites in CSF at clinically relevant concentrations. These channels directly mediate Ca^{2+} inflow to initiate divergent signaling cascades underpinning major phenotypical changes in neuronal excitability and injury associated with HB pathology. Our findings also implicated the bilirubin-binding cavity in the TRPM2 channel as a potential target for developing therapeutics to alleviate ischemic injury in stroke and other HB-related neuronal injuries.

The actions of bilirubin are intricate and may engage both direct and indirect signaling pathways. Previous studies have shown that HB can create an oxidative stress microenvironment by promoting the production of ROS[32] and that oxidative or nitrosative stress induces the production of free ADPR from nicotinamide adenine dinucleotide (NAD) catalyzed by NADase in mitochondria and by PARP in the nucleus.[65,66] ADPR generated by these pathways can serve as intracellular agonists to activate the TRPM2 channel. Indeed, we found a significant reduction in the amplitude of bilirubin-induced TRPM2 channel currents by PJ-34, which blocks PARP-dependent ADPR production in the nucleus, suggesting that a considerable portion of bilirubin action was synergistic with intracellular ADPR production. Similarly, after chelation of intracellular Ca^{2+} by BAPTA, we found that the amplitude of TRPM2 channel currents induced by bilirubin was also significantly reduced. Thus, the action of bilirubin on TRPM2 channels is convergent with that of intracellular ADPR and Ca^{2+}. However, we would argue that these were largely indirect consequences following direct activation of Ca^{2+}-permeable TRPM2 channels by bilirubin. Multiple lines of evidence provide strong support of this view: (1) bilirubin directly gates the opening of TRPM2 channels at the single-channel level; (2) bilirubin activates the TRPM2 channels despite mutational disruption of their sensitivity to ADPR and Ca^{2+}; (3) structurally related derivatives of bilirubin activate TRPM2 channels with varying potency, and are competitively antagonized by A23, which possesses much higher affinity for the same binding site; and (4) bilirubin actions on both neuronal excitability and neurotoxicity are ablated in TRPM2 *D1066A* KI mice. Therefore, we concluded that bilirubin is the first messenger molecule directly acting upon the plasma membrane TRPM2 channel to initiate downstream signaling pathways that amplify a vicious cycle of overexcitation, Ca^{2+} overload, and ROS and ADPR production, ultimately driving cell death during ischemia.

Our results from TRPM2 mutants led us to suggest that bilirubin must gate TRPM2 channels through a noncanonical binding site. Huang et al.[55] suggested two distinctive ADPR-binding sites in the hTRPM2 channel, one formed by the N-terminal MHR1/2 domains and the other by the C-terminal NUDT9-H domain, both of which work in concert with Ca^{2+} binding to the sites in the transmembrane domain to gate the channel opening. However, our results showed that introduction of point mutations in the MHR1/2 domain (i.e., R302A/R358A), NUDT9-H domain (i.e., R1433A), or truncations of both domains (i.e., TRPM2-ΔN/ΔC) prevent activation of the TRPM2 channel by ADPR as anticipated, but not by bilirubin. The binding site for bilirubin to activate the TRPM2 channel is, therefore, independent of that for ADPR (Figure 6). Our *in silico* computational simulations complemented by site-directed mutagenesis guided us to demonstrate that introduction of K928A/D1069A mutation eliminates the activation of the TRPM2 channel by bilirubin but not ADPR or Ca^{2+}, delineating the structural basis of bilirubin binding within the cavity surrounded by the S3, S5, and TRP helix. It should be noted that bilirubin might also directly interact with Ca^{2+} binding sites, as inferred by both H-bond and π-π interactions of its pyrrole moiety with D866 and W868 in the vicinity of the Ca^{2+}-binding pocket, reducing the requirement of intracellular Ca^{2+} for channel gating. Interestingly, the location of the bilirubin-binding pocket in the TRPM2 channel is proximal to that for icilin in the TRPM8 channel.[67] The structural similarity between these two channels suggests a conserved cavity for agonist binding of TRP channels. The bilirubin-binding site is located deep in the transmembrane region and access to this binding cavity can take time, as elucidated by our MD simulations of the ligand-TRMP2 complex (Figure S10), explaining the relatively slower action of bilirubin compared with its structural derivative XAME, which has a smaller molecular size and can more readily reach the binding site. Our findings indicated that the TRPM2 channel possesses a novel binding pocket for bilirubin and its derivatives that gate the channel extracellularly via a mechanism distinct from that of intracellular APDR and Ca^{2+}.

When HB occurs in adults, non-histaminergic pruritus caused by abnormal bilirubin levels (>5 mg/dL) is sometimes observed in patients with jaundice.[68] Bilirubin, as a pruritogen, can produce itching by acting on the Mas-related G-protein coupled receptor X4 (MRGPRX4) receptor in humans and MRGPRA1 in mice.[69] MRGPRX4 and MRGPRA1 are mainly distributed in the periphery, specifically in sensory neurons within the dorsal root ganglia (DRG).[70] Considering that MRGPRs serve as G-protein-coupled metabotropic receptors for bilirubin,[4] our current work raises the possibility that bilirubin may also act on TRPM2 channels, which are known to be expressed in DRG neurons, contributing to the phenomenon of pruritus. Given the fact that bilirubin can act as an agonist for both ligand-gated channels (i.e., TRPM2) and metabotropic receptors (i.e., MRGPRA1 and MRGPRX4), we propose that bilirubin largely fulfills the criteria as a volume neurotransmitter involved in physiological and pathological functions in both central and peripheral nervous systems. Although further studies are needed to firmly establish bilirubin as a volume transmitter, our proposal is supported by the findings from this study, complemented by the observations that TRPM2 antagonist A23 specifically competes against the same site with bilirubin, and that specific pathways for endogenous synthesis and metabolism of bilirubin exist in neurons, independent of circulating bilirubin produced by the liver and bile.[71,72] Although the properties of bilirubin storage, degradation, and release modes from neurons remain unknown, we suggest that TRPM2 can function as a ligand-gated non-selective ion channel and/or a metabolic sensing channel for endogenously released bilirubin and its structural derivatives under physiological conditions, potentially playing an important role in regulating intrinsic excitability, synaptic transmission, and plasticity, aside from its pathological roles in ischemic stroke as demonstrated in this study.

Ischemic neuronal death is mainly caused by activation of NMDA receptors and TRPM channels (e.g., TRPM2, 4, and 7) and their reciprocal cross talk via intracellular ROS, ADPR, and/or Ca^{2+}.[73–76] However, antagonists for NMDARs have largely been proven to be clinically ineffective for neuroprotection against stroke, raising the possibility that other mechanisms exist to activate TRPM channels and signaling pathways that cause cell death. Although clinical studies have shown a possible correlation between bilirubin levels and symptomatic severity in stroke patients, it remains elusive how stroke leads to an elevated bilirubin level. Previous studies suggested that excessive heme could be released from cytoplasmic proteins and mitochondrial cytochromes from cells injured or undergoing necrosis in the core of infarct region during ischemia.[77] Moreover, RBC extravasation provides another source of heme that could then be converted to bilirubin in the brain and CSF.[78] As shown by our immunohistochemistry (IHC) co-localization of endogenous enzymes for bilirubin synthesis in neurons, we suggest that in WT mice, ischemia induced elevation in the level of endogenous heme from neurons and/or RBC extravasation from the infarct as well as in the activity of HO in a TRPM2-dependent manner in the infarct area.[79] These converge to boosting levels of UCB (or bilirubin) and/or other more metabolic derivatives in CSF, as modeled by our *in vitro* OGD experiments (Figure 7I). Under stroke circumstances, local UCB concentrations in the infarct area may greatly exceed the global level in CSF: local TB levels sometimes increase to a staggering 25 μmol/L. Such a high concentration will effectively activate TRPM2 channels to exacerbate the severity of cell death. This is evidenced by doubling of infarct volume in stroke patients from MRI scan images or in mouse tMCAO models with a 1-fold increase in DB levels (Figure 1).

Using an *in vitro* cell-death assay with/without TRPM2 antagonist A23, tMCAO models from WT, and TRPM2 knockout mice, we have demonstrated that HB aggravates brain damage primarily through the TRPM2 channel. Using the *D1066A* KI transgenic mice, we showed that bilirubin indeed binds to the transmembrane binding pocket of the TRPM2 channel via a key interaction with the residue D1066 (or amino acid residue D1069 in hTRPM2) and initiates and aggravates excitotoxicity *in vivo*. Although it remains unknown how TRPM2 regulates the release of endogenous bilirubin from neurons and/or other peripheral cells and/or its clearance during stroke, the observation that D1066A mutation abolishes the neurotoxic component of the TRPM2 channel in ischemia-hypoxic brain injury raises the conceptual possibility of differentiating the physiological and pathological signaling embedded in the channel itself (Figure 8). These findings highlight the unexpected roles of bilirubin and its metabolites in activating TRPM2 channels to underlie neurotoxicity independently from canonical signaling pathways.

In this study, we boosted the level of bilirubin via acute injections into mice before tMCAO surgery to closely model the subset of the human cohort with high levels of serum bilirubin after stroke. Administration of exogenous bilirubin limits the extrapolation of our findings from mice to the reciprocal relationship between endogenous bilirubin and brain injury as seen in stroke patients. Given that TRPM2 is widely expressed in both central and peripheral nervous systems and that non-neuronal cells such as microglia also express TRPM2,[80] we envision that deleting TRPM2 in a system-, region-, and cell-specific manner with the Cre-*loxP* system from TRPM2 flox/flox mice is needed in future studies to clearly define the origin and sites of actions of bilirubin and its regulation in healthy and diseased brains.

Conclusions

In conclusion, our study demonstrates that bilirubin directly binds to and activates the TRPM2 channel via a specific binding cavity in the transmembrane domain and that this cavity can be targeted by competitive blockers, such as A23. Being the first messenger for TRPM2 channels, bilirubin triggers and exacerbates neurotoxicity associated with brain ischemia by directly driving Ca^{2+} influx through these channels to elevate neuronal excitability and activate Ca^{2+}-dependent cell-death pathways. Our findings provide mechanistic insights and a proof of principle for developing new strategies targeting the bilirubin-binding pocket in TRPM2 channels to alleviate and prevent brain injury associated with stroke and jaundice in patients.

STAR★METHODS

Detailed methods are provided in the online version of this paper and include the following:

- KEY RESOURCES TABLE
- RESOURCE AVAILABILITY
  - Lead contact
  - Materials availability
  - Data and code availability
- EXPERIMENTAL MODEL AND SUBJECT DETAILS
  - Ethical approval for data analysis of stroke patient blood and MRI
  - Animal and ethical approval
  - Cell culture and transfection of human embryonic kidney 293 cell line
- METHOD DETAILS
  - Enrolled clinical data
  - Preparation of brain slices
  - Transient middle cerebral occlusion (tMCAO) animal model
  - Laser speckle imaging
  - Infarct volume measurement
  - Total bilirubin (TB) and unconjugated bilirubin (UCB) measurement
  - Oxygen-glucose deprivation model in brain slices
  - Immunofluorescence
  - Molecular docking simulations
  - Molecular dynamics (MD) simulation
  - Cell death assess with calcein-am/PI co-staining
  - Electrophysiology
  - Solutions
- QUANTIFICATION AND STATISTICAL ANALYSIS

SUPPLEMENTAL INFORMATION

Supplemental information can be found online at https://doi.org/10.1016/j.neuron.2023.02.022.

CellPress
OPEN ACCESS

ACKNOWLEDGMENTS

This work was supported by the National Natural Science Foundation of China (81720108010 to S.-K.Y., 81870722 and 82020108008 to H.-B.S., 81800903 to M.L., 81900935 to X.-L.Y., and 82000993 to H.-S.S.); the Science and Technology Commission of Shanghai (19YF1437600 to X.-L.Y.); the Wellcome Trust (072275/Z/03/Z) and Alzheimer's Research UK(ART/PPG2009A/2) (to L.-H.J.); and CIHR (PJT-156034 and PJT-156439), NSERC (RGPIN-2017-06665), and Canada Research Chair Program (to L.-Y.W.). We thank Drs. Adam Fekete, Bassam Tawfik, and Alex Wang for critical comments on the earlier versions of this manuscript and English proofing and Dr. Yu-Zheng Zhao for the UnaG protein.

AUTHOR CONTRIBUTIONS

H.-W.L. and L.-N.G. carried out experiments *in vitro* and collected the data. K.L., Z.-Q.L., and M.-X.L. performed computer simulations, the tMCAO animal model, and blood pressure/serum biochemical indicators monitoring and collected data. X.-F.Y., W.Y., and L.-H.J. provided the TRPM2 null mice and TRPM2 plasmids and helped with writing. X.-L.Y., M.L., and H.-S.S. collected clinical data. H.-B.S., S.-K.Y., and L.-Y.W. designed this study and contributed to the manuscript revisions. L.-Y.W. supervised the experiments and wrote the manuscript with H.-W.L.

DECLARATION OF INTERESTS

We have filed a patent application for the bilirubin-binding cavity on the TRPM2 channel for developing therapeutics to treat brain injury in stroke and jaundice.

Received: December 29, 2021
Revised: October 18, 2022
Accepted: February 13, 2023
Published: March 14, 2023

REFERENCES

1. GBD 2015 Neurological Disorders Collaborator Group (2017). Global, regional, and national burden of neurological disorders during 1990–2015: a systematic analysis for the Global Burden of Disease Study 2015. Lancet Neurol. *16*, 877–897. https://doi.org/10.1016/S1474-4422(17)30299-5.
2. Libby, P., Buring, J.E., Badimon, L., Hansson, G.K., Deanfield, J., Bittencourt, M.S., Tokgözoğlu, L., and Lewis, E.F. (2019). Atherosclerosis. Nat. Rev. Dis. Primers *5*, 56. https://doi.org/10.1038/s41572-019-0106-z.
3. Kamel, H., Okin, P.M., Elkind, M.S., and Iadecola, C. (2016). Atrial fibrillation and mechanisms of stroke: time for a new model. Stroke *47*, 895–900. https://doi.org/10.1161/STROKEAHA.115.012004.
4. McDonagh, A.F. (2010). Controversies in bilirubin biochemistry and their clinical relevance. Semin. Fetal Neonatal Med. *15*, 141–147. https://doi.org/10.1016/j.siny.2009.10.005.
5. Donnan, G.A., Fisher, M., Macleod, M., and Davis, S.M. (2008). Stroke. Lancet *371*, 1612–1623. https://doi.org/10.1016/S0140-6736(08)60694-7.
6. Lai, T.W., Zhang, S., and Wang, Y.T. (2014). Excitotoxicity and stroke: identifying novel targets for neuroprotection. Prog. Neurobiol. *115*, 157–188. https://doi.org/10.1016/j.pneurobio.2013.11.006.
7. Chamorro, Á., Dirnagl, U., Urra, X., and Planas, A.M. (2016). Neuroprotection in acute stroke: targeting excitotoxicity, oxidative and nitrosative stress, and inflammation. Lancet Neurol. *15*, 869–881. https://doi.org/10.1016/S1474-4422(16)00114-9.
8. Sullivan, J.I., and Rockey, D.C. (2017). Diagnosis and evaluation of hyperbilirubinemia. Curr. Opin. Gastroenterol. *33*, 164–170. https://doi.org/10.1097/MOG.0000000000000354.
9. Brito, M.A., Rosa, A.I., Falcão, A.S., Fernandes, A., Silva, R.F., Butterfield, D.A., and Brites, D. (2008). Unconjugated bilirubin differentially affects the redox status of neuronal and astroglial cells. Neurobiol. Dis. *29*, 30–40. https://doi.org/10.1016/j.nbd.2007.07.023.
10. Lai, K., Song, X.L., Shi, H.S., Qi, X., Li, C.Y., Fang, J., Wang, F., Maximyuk, O., Krishtal, O., Xu, T.L., et al. (2020). Bilirubin enhances the activity of ASIC channels to exacerbate neurotoxicity in neonatal hyperbilirubinemia in mice. Sci. Transl. Med. *12*, eaax1337. https://doi.org/10.1126/scitranslmed.aax1337.
11. Liang, M., Yin, X.L., Shi, H.B., Li, C.Y., Li, X.Y., Song, N.Y., Shi, H.S., Zhao, Y., Wang, L.Y., and Yin, S.K. (2017). Bilirubin augments Ca(2+) load of developing bushy neurons by targeting specific subtype of voltage-gated calcium channels. Sci. Rep. *7*, 431. https://doi.org/10.1038/s41598-017-00275-9.
12. Pazar, A., Kolgazi, M., Memisoglu, A., Bahadir, E., Sirvanci, S., Yaman, A., Yeğen, B.Ç., and Ozek, E. (2016). The neuroprotective and anti-apoptotic effects of melatonin on hemolytic hyperbilirubinemia-induced oxidative brain damage. J. Pineal Res. *60*, 74–83. https://doi.org/10.1111/jpi.12292.
13. Rauti, R., Qaisiya, M., Tiribelli, C., Ballerini, L., and Bellarosa, C. (2020). Bilirubin disrupts calcium homeostasis in neonatal hippocampal neurons: a new pathway of neurotoxicity. Arch. Toxicol. *94*, 845–855. https://doi.org/10.1007/s00204-020-02659-9.
14. Qaisiya, M., Brischetto, C., Jašprová, J., Vitek, L., Tiribelli, C., and Bellarosa, C. (2017). Bilirubin-induced ER stress contributes to the inflammatory response and apoptosis in neuronal cells. Arch. Toxicol. *91*, 1847–1858. https://doi.org/10.1007/s00204-016-1835-3.
15. Vodret, S., Bortolussi, G., Iaconcig, A., Martinelli, E., Tiribelli, C., and Muro, A.F. (2018). Attenuation of neuro-inflammation improves survival and neurodegeneration in a mouse model of severe neonatal hyperbilirubinemia. Brain Behav. Immun. *70*, 166–178. https://doi.org/10.1016/j.bbi.2018.02.011.
16. Luo, Y., Li, J., Zhang, J., and Xu, Y. (2013). Elevated bilirubin after acute ischemic stroke linked to the stroke severity. Int. J. Dev. Neurosci. *31*, 634–638. https://doi.org/10.1016/j.ijdevneu.2013.08.002.
17. Chen, X.J., Zhou, H.Q., Ye, H.B., Li, C.Y., and Zhang, W.T. (2016). The effect of bilirubin on the excitability of mitral cells in the olfactory bulb of the rat. Sci. Rep. *6*, 32872. https://doi.org/10.1038/srep32872.
18. Shi, H.S., Lai, K., Yin, X.L., Liang, M., Ye, H.B., Shi, H.B., Wang, L.Y., and Yin, S.K. (2019). Ca(2+)-dependent recruitment of voltage-gated sodium channels underlies bilirubin-induced overexcitation and neurotoxicity. Cell Death Dis. *10*, 774. https://doi.org/10.1038/s41419-019-1979-1.
19. Hara, Y., Wakamori, M., Ishii, M., Maeno, E., Nishida, M., Yoshida, T., Yamada, H., Shimizu, S., Mori, E., Kudoh, J., et al. (2002). LTRPC2 Ca2+-permeable channel activated by changes in redox status confers susceptibility to cell death. Mol. Cell *9*, 163–173. https://doi.org/10.1016/s1097-2765(01)00438-5.
20. Togashi, K., Hara, Y., Tominaga, T., Higashi, T., Konishi, Y., Mori, Y., and Tominaga, M. (2006). TRPM2 activation by cyclic ADP-ribose at body temperature is involved in insulin secretion. EMBO J. *25*, 1804–1815. https://doi.org/10.1038/sj.emboj.7601083.
21. Yamamoto, S., Shimizu, S., Kiyonaka, S., Takahashi, N., Wajima, T., Hara, Y., Negoro, T., Hiroi, T., Kiuchi, Y., Okada, T., et al. (2008). TRPM2-mediated Ca2+influx induces chemokine production in monocytes that aggravates inflammatory neutrophil infiltration. Nat. Med. *14*, 738–747. https://doi.org/10.1038/nm1758.
22. Mittal, M., Nepal, S., Tsukasaki, Y., Hecquet, C.M., Soni, D., Rehman, J., Tiruppathi, C., and Malik, A.B. (2017). Neutrophil activation of endothelial cell-expressed TRPM2 mediates transendothelial neutrophil migration and vascular injury. Circ. Res. *121*, 1081–1091. https://doi.org/10.1161/CIRCRESAHA.117.311747.
23. Gershkovitz, M., Fainsod-Levi, T., Zelter, T., Sionov, R.V., and Granot, Z. (2019). TRPM2 modulates neutrophil attraction to murine tumor cells by regulating CXCL2 expression. Cancer Immunol. Immunother. *68*, 33–43. https://doi.org/10.1007/s00262-018-2249-2.
24. Beceiro, S., Radin, J.N., Chatuvedi, R., Piazuelo, M.B., Horvarth, D.J., Cortado, H., Gu, Y., Dixon, B., Gu, C., Lange, I., et al. (2017). TRPM2 ion channels regulate macrophage polarization and gastric inflammation during Helicobacter pylori infection. Mucosal Immunol. *10*, 493–507. https://doi.org/10.1038/mi.2016.60.

25. Wehrhahn, J., Kraft, R., Harteneck, C., and Hauschildt, S. (2010). Transient receptor potential melastatin 2 is required for lipopolysaccharide-induced cytokine production in human monocytes. J. Immunol. *184*, 2386–2393. https://doi.org/10.4049/jimmunol.0902474.

26. Olsen, N.J., Spurlock, C.F., 3rd, and Aune, T.M. (2014). Methotrexate induces production of IL-1 and IL-6 in the monocytic cell line U937. Arthritis Res. Ther. *16*, R17. https://doi.org/10.1186/ar4444.

27. Beck, A., Kolisek, M., Bagley, L.A., Fleig, A., and Penner, R. (2006). Nicotinic acid adenine dinucleotide phosphate and cyclic ADP-ribose regulate TRPM2 channels in T lymphocytes. FASEB J. *20*, 962–964. https://doi.org/10.1096/fj.05-5538fje.

28. Buelow, B., Song, Y., and Scharenberg, A.M. (2008). The poly(ADP-ribose) polymerase PARP-1 is required for oxidative stress-induced TRPM2 activation in lymphocytes. J. Biol. Chem. *283*, 24571–24583. https://doi.org/10.1074/jbc.M802673200.

29. Fonfria, E., Murdock, P.R., Cusdin, F.S., Benham, C.D., Kelsell, R.E., and McNulty, S. (2006). Tissue distribution profiles of the human TRPM cation channel family. J. Recept. Signal Transduct. Res. *26*, 159–178. https://doi.org/10.1080/10799890600637506.

30. Tan, C.H., and McNaughton, P.A. (2016). The TRPM2 ion channel is required for sensitivity to warmth. Nature *536*, 460–463. https://doi.org/10.1038/nature19074.

31. Fonfria, E., Marshall, I.C., Benham, C.D., Boyfield, I., Brown, J.D., Hill, K., Hughes, J.P., Skaper, S.D., and McNulty, S. (2004). TRPM2 channel opening in response to oxidative stress is dependent on activation of poly(ADP-ribose) polymerase. Br. J. Pharmacol. *143*, 186–192. https://doi.org/10.1038/sj.bjp.0705914.

32. Perraud, A.L., Takanishi, C.L., Shen, B., Kang, S., Smith, M.K., Schmitz, C., Knowles, H.M., Ferraris, D., Li, W., Zhang, J., et al. (2005). Accumulation of free ADP-ribose from mitochondria mediates oxidative stress-induced gating of TRPM2 cation channels. J. Biol. Chem. *280*, 6138–6148. https://doi.org/10.1074/jbc.M411446200.

33. Lee, C.R., Machold, R.P., Witkovsky, P., and Rice, M.E. (2013). TRPM2 channels are required for NMDA-induced burst firing and contribute to H(2)O(2)-dependent modulation in substantia nigra pars reticulata GABAergic neurons. J. Neurosci. *33*, 1157–1168. https://doi.org/10.1523/JNEUROSCI.2832-12.2013.

34. Yan, J., Bengtson, C.P., Buchthal, B., Hagenston, A.M., and Bading, H. (2020). Coupling of NMDA receptors and TRPM4 guides discovery of unconventional neuroprotectants. Science *370*, eaay3302. https://doi.org/10.1126/science.aay3302.

35. Schiavon, E., Smalley, J.L., Newton, S., Greig, N.H., and Forsythe, I.D. (2018). Neuroinflammation and ER-stress are key mechanisms of acute bilirubin toxicity and hearing loss in a mouse model. PLoS One *13*, e0201022. https://doi.org/10.1371/journal.pone.0201022.

36. Vitek, L., Hubacek, J.A., Pajak, A., Doryńska, A., Kozela, M., Eremiasova, L., Danzig, V., Stefler, D., and Bobak, M. (2019). Association between plasma bilirubin and mortality. Ann. Hepatol. *18*, 379–385. https://doi.org/10.1016/j.aohep.2019.02.001.

37. Bulmer, A.C., Bakrania, B., Du Toit, E.F., Boon, A.C., Clark, P.J., Powell, L.W., Wagner, K.H., and Headrick, J.P. (2018). Bilirubin acts as a multipotent guardian of cardiovascular integrity: more than just a radical idea. Am. J. Physiol. Heart Circ. Physiol. *315*, H429–H447. https://doi.org/10.1152/ajpheart.00417.2017.

38. Pineda, S., Bang, O.Y., Saver, J.L., Starkman, S., Yun, S.W., Liebeskind, D.S., Kim, D., Ali, L.K., Shah, S.H., and Ovbiagele, B. (2008). Association of serum bilirubin with ischemic stroke outcomes. J. Stroke Cerebrovasc. Dis. *17*, 147–152. https://doi.org/10.1016/j.jstrokecerebrovasdis.2008.01.009.

39. Luo, Y., Li, J.W., Lu, Z.J., Wang, C., Guan, D.N., and Xu, Y. (2012). Serum bilirubin after acute ischemic stroke is associated with stroke severity. Curr. Neurovasc. Res. *9*, 128–132. https://doi.org/10.2174/156720212800410876.

40. Arundine, M., and Tymianski, M. (2004). Molecular mechanisms of glutamate-dependent neurodegeneration in ischemia and traumatic brain injury. Cell. Mol. Life Sci. *61*, 657–668. https://doi.org/10.1007/s00018-003-3319-x.

41. Alim, I., Teves, L., Li, R., Mori, Y., and Tymianski, M. (2013). Modulation of NMDAR subunit expression by TRPM2 channels regulates neuronal vulnerability to ischemic cell death. J. Neurosci. *33*, 17264–17277. https://doi.org/10.1523/JNEUROSCI.1729-13.2013.

42. Ostrow, J.D., Pascolo, L., Brites, D., and Tiribelli, C. (2004). Molecular basis of bilirubin-induced neurotoxicity. Trends Mol. Med. *10*, 65–70. https://doi.org/10.1016/j.molmed.2003.12.003.

43. Hulzebos, C.V., and Dijk, P.H. (2014). Bilirubin-albumin binding, bilirubin/albumin ratios, and free bilirubin levels: where do we stand? Semin. Perinatol. *38*, 412–421. https://doi.org/10.1053/j.semperi.2014.08.004.

44. Amin, S.B., and Wang, H. (2018). Bilirubin albumin binding and unbound unconjugated hyperbilirubinemia in premature infants. J. Pediatr. *192*, 47–52. https://doi.org/10.1016/j.jpeds.2017.09.039.

45. Roseth, S., Hansen, T.W., Fonnum, F., and Walaas, S.I. (1998). Bilirubin inhibits transport of neurotransmitters in synaptic vesicles. Pediatr. Res. *44*, 312–316. https://doi.org/10.1203/00006450-199809000-00008.

46. Fauzee, N.J., Pan, J., and Wang, Y.L. (2010). PARP and PARG inhibitors–new therapeutic targets in cancer treatment. Pathol. Oncol. Res. *16*, 469–478. https://doi.org/10.1007/s12253-010-9266-6.

47. Starkus, J., Beck, A., Fleig, A., and Penner, R. (2007). Regulation of TRPM2 by extra- and intracellular calcium. J. Gen. Physiol. *130*, 427–440. https://doi.org/10.1085/jgp.200709836.

48. Perraud, A.L., Fleig, A., Dunn, C.A., Bagley, L.A., Launay, P., Schmitz, C., Stokes, A.J., Zhu, Q., Bessman, M.J., Penner, R., et al. (2001). ADP-ribose gating of the calcium-permeable LTRPC2 channel revealed by Nudix motif homology. Nature *411*, 595–599. https://doi.org/10.1038/35079100.

49. Du, J., Xie, J., and Yue, L. (2009). Modulation of TRPM2 by acidic pH and the underlying mechanisms for pH sensitivity. J. Gen. Physiol. *134*, 471–488. https://doi.org/10.1085/jgp.200910254.

50. Kühn, F.J.P., Witschas, K., Kühn, C., and Lückhoff, A. (2010). Contribution of the S5-pore-S6 domain to the gating characteristics of the cation channels TRPM2 and TRPM8. J. Biol. Chem. *285*, 26806–26814. https://doi.org/10.1074/jbc.M110.109975.

51. Zeng, B., Chen, G.L., and Xu, S.Z. (2012). Divalent copper is a potent extracellular blocker for TRPM2 channel. Biochem. Biophys. Res. Commun. *424*, 279–284. https://doi.org/10.1016/j.bbrc.2012.06.107.

52. Salmon, M., and Fenselau, C. (1974). Evaluation of bilirubin derivatives for mass spectral analysis. Biomed. Mass Spectrom. *1*, 219–222. https://doi.org/10.1002/bms.1200010404.

53. Lightner, D.A., Linnane, W.P., 3rd, and Ahlfors, C.E. (1984). Bilirubin photooxidation products in the urine of jaundiced neonates receiving phototherapy. Pediatr. Res. *18*, 696–700. https://doi.org/10.1203/00006450-198408000-00003.

54. Iyanagi, T., Emi, Y., and Ikushiro, S. (1998). Biochemical and molecular aspects of genetic disorders of bilirubin metabolism. Biochim. Biophys. Acta *1407*, 173–184. https://doi.org/10.1016/s0925-4439(98)00044-1.

55. Huang, Y., Roth, B., Lü, W., and Du, J. (2019). Ligand recognition and gating mechanism through three ligand-binding sites of human TRPM2 channel. eLife *8*, e50175. https://doi.org/10.7554/eLife.50175.

56. Du, J., Xie, J., and Yue, L. (2009). Intracellular calcium activates TRPM2 and its alternative spliced isoforms. Proc. Natl. Acad. Sci. USA *106*, 7239–7244. https://doi.org/10.1073/pnas.0811725106.

57. Shimizu, T., Macey, T.A., Quillinan, N., Klawitter, J., Perraud, A.L., Traystman, R.J., and Herson, P.S. (2013). Androgen and PARP-1 regulation of TRPM2 channels after ischemic injury. J. Cereb. Blood Flow Metab. *33*, 1549–1555. https://doi.org/10.1038/jcbfm.2013.105.

58. Shimizu, T., Dietz, R.M., Cruz-Torres, I., Strnad, F., Garske, A.K., Moreno, M., Venna, V.R., Quillinan, N., and Herson, P.S. (2016). Extended therapeutic window of a novel peptide inhibitor of TRPM2 channels following focal cerebral ischemia. Exp. Neurol. *283*, 151–156. https://doi.org/10.1016/j.expneurol.2016.06.015.

CellPress
OPEN ACCESS

59. Zhang, H., Yu, P., Lin, H., Jin, Z., Zhao, S., Zhang, Y., Xu, Q., Jin, H., Liu, Z., Yang, W., and Zhang, L. (2021). The discovery of novel ACA derivatives as specific TRPM2 inhibitors that reduce ischemic injury both in vitro and in vivo. J. Med. Chem. *64*, 3976–3996. https://doi.org/10.1021/acs.jmedchem.0c02129.

60. Vaz, A.R., Silva, S.L., Barateiro, A., Falcão, A.S., Fernandes, A., Brito, M.A., and Brites, D. (2011). Selective vulnerability of rat brain regions to unconjugated bilirubin. Mol. Cell. Neurosci. *48*, 82–93. https://doi.org/10.1016/j.mcn.2011.06.008.

61. Brito, M.A., Lima, S., Fernandes, A., Falcão, A.S., Silva, R.F., Butterfield, D.A., and Brites, D. (2008). Bilirubin injury to neurons: contribution of oxidative stress and rescue by glycoursodeoxycholic acid. Neurotoxicology *29*, 259–269. https://doi.org/10.1016/j.neuro.2007.11.002.

62. Bortolussi, G., Codarin, E., Antoniali, G., Vascotto, C., Vodret, S., Arena, S., Cesaratto, L., Scaloni, A., Tell, G., and Muro, A.F. (2015). Impairment of enzymatic antioxidant defenses is associated with bilirubin-induced neuronal cell death in the cerebellum of Ugt1 KO mice. Cell Death Dis. *6*, e1739. https://doi.org/10.1038/cddis.2015.113.

63. Gürses, D., Kiliç, I., and Sahiner, T. (2002). Effects of hyperbilirubinemia on cerebrocortical electrical activity in newborns. Pediatr. Res. *52*, 125–130. https://doi.org/10.1203/00006450-200207000-00023.

64. Yin, X.L., Jie, H.Q., Liang, M., Gong, L.N., Liu, H.W., Pan, H.L., Xing, Y.Z., Shi, H.B., Li, C.Y., Wang, L.Y., and Yin, S.-K. (2018). Accelerated development of the first-order central auditory neurons with spontaneous activity. Front. Mol. Neurosci. *11*, 183. https://doi.org/10.3389/fnmol.2018.00183.

65. Gou, P., Qi, X., Yuan, R., Li, H., Gao, X., Wang, J., and Zhang, B. (2018). Tet1-mediated DNA demethylation involves in neuron damage induced by bilirubin in vitro. Toxicol. Mech. Methods *28*, 55–61. https://doi.org/10.1080/15376516.2017.1357775.

66. Zhang, W., Hirschler-Laszkiewicz, I., Tong, Q., Conrad, K., Sun, S.C., Penn, L., Barber, D.L., Stahl, R., Carey, D.J., Cheung, J.Y., and Miller, B.A. (2006). TRPM2 is an ion channel that modulates hematopoietic cell death through activation of caspases and PARP cleavage. Am. J. Physiol. Cell Physiol. *290*, C1146–C1159. https://doi.org/10.1152/ajpcell.00205.2005.

67. Yin, Y., Le, S.C., Hsu, A.L., Borgnia, M.J., Yang, H., and Lee, S.Y. (2019). Structural basis of cooling agent and lipid sensing by the cold-activated TRPM8 channel. Science *363*, eaav9334. https://doi.org/10.1126/science.aav9334.

68. Bassari, R., and Koea, J.B. (2015). Jaundice associated pruritis: a review of pathophysiology and treatment. World J. Gastroenterol. *21*, 1404–1413. https://doi.org/10.3748/wjg.v21.i5.1404.

69. Meixiong, J., Vasavda, C., Green, D., Zheng, Q., Qi, L., Kwatra, S.G., Hamilton, J.P., Snyder, S.H., and Dong, X. (2019). Identification of a bilirubin receptor that may mediate a component of cholestatic itch. eLife *8*, e44116. https://doi.org/10.7554/eLife.44116.

70. Dong, X., Han, S., Zylka, M.J., Simon, M.I., and Anderson, D.J. (2001). A diverse family of GPCRs expressed in specific subsets of nociceptive sensory neurons. Cell *106*, 619–632. https://doi.org/10.1016/S0092-8674(01)00483-4.

71. Barañano, D.E., and Snyder, S.H. (2001). Neural roles for heme oxygenase: contrasts to nitric oxide synthase. Proc. Natl. Acad. Sci. USA *98*, 10996–11002. https://doi.org/10.1073/pnas.191351298.

72. Huang, E., Ong, W.Y., Go, M.L., and Garey, L.J. (2005). Heme oxygenase-1 activity after excitotoxic injury: immunohistochemical localization of bilirubin in neurons and astrocytes and deleterious effects of heme oxygenase inhibition on neuronal survival after kainate treatment. J. Neurosci. Res. *80*, 268–278. https://doi.org/10.1002/jnr.20444.

73. MacDonald, J.F., Xiong, Z.G., and Jackson, M.F. (2006). Paradox of Ca2+ signaling, cell death and stroke. Trends Neurosci. *29*, 75–81. https://doi.org/10.1016/j.tins.2005.12.001.

74. Tu, W., Xu, X., Peng, L., Zhong, X., Zhang, W., Soundarapandian, M.M., Balel, C., Wang, M., Jia, N., Zhang, W., et al. (2010). DAPK1 interaction with NMDA receptor NR2B subunits mediates brain damage in stroke. Cell *140*, 222–234. https://doi.org/10.1016/j.cell.2009.12.055.

75. Gelderblom, M., Melzer, N., Schattling, B., Göb, E., Hicking, G., Arunachalam, P., Bittner, S., Ufer, F., Herrmann, A.M., Bernreuther, C., et al. (2014). Transient receptor potential melastatin subfamily member 2 cation channel regulates detrimental immune cell invasion in ischemic stroke. Stroke *45*, 3395–3402. https://doi.org/10.1161/STROKEAHA.114.005836.

76. Gotru, S.K., Chen, W., Kraft, P., Becker, I.C., Wolf, K., Stritt, S., Zierler, S., Hermanns, H.M., Rao, D., Perraud, A.L., et al. (2018). TRPM7 kinase controls calcium responses in arterial thrombosis and stroke in mice. Arterioscler. Thromb. Vasc. Biol. *38*, 344–352. https://doi.org/10.1161/ATVBAHA.117.310391.

77. Wagner, K.R., Sharp, F.R., Ardizzone, T.D., Lu, A., and Clark, J.F. (2003). Heme and iron metabolism: role in cerebral hemorrhage. J. Cereb. Blood Flow Metab. *23*, 629–652. https://doi.org/10.1097/01.WCB.0000073905.87928.6D.

78. Jiang, X., Andjelkovic, A.V., Zhu, L., Yang, T., Bennett, M.V.L., Chen, J., Keep, R.F., and Shi, Y. (2018). Blood-brain barrier dysfunction and recovery after ischemic stroke. Prog. Neurobiol. *163–164*, 144–171. https://doi.org/10.1016/j.pneurobio.2017.10.001.

79. Nimura, T., Weinstein, P.R., Massa, S.M., Panter, S., and Sharp, F.R. (1996). Heme oxygenase-1 (HO-1) protein induction in rat brain following focal ischemia. Brain Res. Mol. Brain Res. *37*, 201–208. https://doi.org/10.1016/0169-328x(95)00315-j.

80. Badimon, A., Strasburger, H.J., Ayata, P., Chen, X., Nair, A., Ikegami, A., Hwang, P., Chan, A.T., Graves, S.M., Uweru, J.O., et al. (2020). Negative feedback control of neuronal activity by microglia. Nature *586*, 417–423. https://doi.org/10.1038/s41586-020-2777-8.

81. Zou, J., Ainscough, J.F., Yang, W., Sedo, A., Yu, S.P., Mei, Z.Z., Sivaprasadarao, A., Beech, D.J., and Jiang, L.H. (2013). A differential role of macrophage TRPM2 channels in Ca^{2+} signaling and cell death in early responses to H_2O_2. Am. J. Physiol. Cell Physiol. *305*, C61–C69. https://doi.org/10.1152/ajpcell.00390.2012.

82. Carriere, C.H., Wang, W.X., Sing, A.D., Fekete, A., Jones, B.E., Yee, Y., Ellegood, J., Maganti, H., Awofala, L., Marocha, J., et al. (2020). The gamma-protocadherins regulate the survival of GABAergic interneurons during developmental cell death. J. Neurosci. *40*, 8652–8668. https://doi.org/10.1523/JNEUROSCI.1636-20.2020.

STAR★METHODS

KEY RESOURCES TABLE

REAGENT or RESOURCE	SOURCE	IDENTIFIER
Antibodies		
Anti-NeuN	Abcam	Cat# ab104224; RRID:AB_10711040
Anti-TRPM2	Gifted by Wei Yang	N/A
Anti-BVR	Enzo life science	Cat# ADI-OSA-400-E; RRID:AB_2038874
Anti-HO-1	Abcam	Cat# ab68477; RRID:AB_11156457
Anti-S100β	Abcam	Cat# ab52642; RRID:AB_882426
Goat anti-mice second antibody	Invitrogen	Cat# A-31553; RRID:AB_221604
Biological samples		
Healthy and tMCAO adult mice brain tissue	Collected in the lab	N/A
Healthy and tMCAO adult mice blood	Collected in the lab	N/A
Healthy and tMCAO adult mice CSF	Collected in the lab	N/A
Chemicals, peptides, and recombinant proteins		
2,3,5-Triphenyl-tetrazolium chloride (TTC)	Sigma, USA	Cat# T8877
A23	Wei Yang	N/A
APV	Sigma, USA	Cat# A5282
Adenosine 5'-diphosphoribose sodium salt	Sigma, USA	Cat# A0752
BAPTA	Sigma, USA	Cat# A4926
Bicuculline	Sigma, USA	Cat# 14340
Bilirubin	Sigma, USA	Cat# B4126
Bilirubin Conjugate, Ditaurate, Disodium Salt	Sigma, USA	Cat# 201102
Biliverdin hydrochloride	Sigma, USA	Cat# 30891
Blasticidin	Invivogen, USA	Cat# ant-bl-5
DMEM	Gibco, USA	Cat# 2263286
DMEM/ F-12	Gibco, USA	Cat# 2186680
EGTA	Sigma, USA	Cat# E4378
Fetal bovine serum	Gibco, USA	Cat# 10099141
Flufenamic acid (FFA)	Sigma, USA	Cat# F9005
Glucose	Sigma, USA	Cat# G8270
HEPES	Sigma, USA	Cat# H3375
Lipofectamine 3000 transfection reagent	Invitrogen, USA	Cat# 2145954
NBQX	Sigma, USA	Cat# N171
PJ-34 hydrochloride hydrate	Sigma, USA	Cat# 528150
Strychnine	Sigma, USA	Cat# 50532
Sucrose	Sangon Biotech	Cat# A502792
Tetracycline	Sigma, USA	Cat# 87128
Trypsin-EDTA (0.25%)	Gibco, USA	Cat# 2042303
UnaG	Yu-Zheng Zhao	N/A
Xanthobilirubic acid methyl ester	Frontier Scientific	Cat# 41053
Zeocin	Invivogen, USA	Cat# ant-zn-5
Critical commercial assays		
Bilirubin Assay Kit	Sigma, USA	Cat# MAK126
Four-color multiple fluorescent immunohistoche-mical staining kit	Absin	Cat# Abs50028
Deposited data		
Human TRPM2 (open) structure	Huang et al.[55]	PDB: 6PUS
Structure of compound bilirubin	NCBI	CID:5280352

(Continued on next page)

CellPress
OPEN ACCESS

Continued

REAGENT or RESOURCE	SOURCE	IDENTIFIER
Experimental models: Cell lines		
HEK293	ATCC	CRL; CVCL_0045
Experimental models: Organisms/strains		
Trpm2$^{-/-}$ mouse	Lin-Hua Jiang	N/A
D1066A knockin mouse	This paper	See Table S4
Oligonucleotides		
TRPM2-ΔN/ΔC	Du et al.[49,56]	N/A
K928A/D1069A	This paper	N/A
Other TRPM2 mutants	Wei Yang	N/A
Recombinant DNA		
pcDNA3.1-human TRPM2	Wei Yang	N/A
Software and algorithms		
Clampfit10.5	Axon Instruments	https://support.moleculardevices.com/
PatchMaster v2x90.5	HEKA Elektronik	https://www.heka.com/
MiniAnalysis	Synaptosoft, NT, USA	http://www.synaptosoft.com/
GraphPad Prism 8	GraphPad Software	http://www.graphpad.com/
Adobe Illustration	Adobe System Inc.	https://www.adobe.com/
OsiriX 12.0	Pixmeo SARL	https://www.osirixviewer.com
SPSS 25.0	SPSS Inc.	https://www.ibm.com/
ZEN Digital Imaging for Light Microscopy	Carl Zeiss Microscopy GmbH, Jena, Germany	http://www.zeiss.com/microscopy
ImageJ	NIH, USA	https://imagej.nih.gov/ij
Schrödinger Maestro	Schrödinger 2020-3	https://www.schrodinger.com/
moorFLPIReviewV50	Moor instruments	https://www.moor.co.uk/
Other		
Micropipette Puller P-1000	Sutter Instruments	https://www.sutter.com
Borsosilicate glass capillaries	World Precision Instruments	https://www.wpiinc.com
Leica VT1000 S Vibrating blade microtome	Leica Biosystems	https://www.leicabiosystems.com/us/
Micromanipulator	Luigs & Neumann	https://www.luigs-neumann.org
Multiclamp 700B	Molecular Devices	https://www.moleculardevices.com
Micromanipulator MP-225	Sutter Instruments	https://www.sutter.com
R500 Small Animal Anesthesia Machine	RWD Life Science	https://www.rwdstco.com
Stereotaxic Frame	RWD Life Science	https://www.rwdstco.com

RESOURCE AVAILABILITY

Lead contact

Further information and requests for resources and reagents should be directed to and will be fulfilled by the lead contact, Lu-Yang Wang (luyang.wang@utoronto.ca).

Materials availability

All plasmids, reagents, and transgenic mice used in this study are available from the lead contact. A completed materials transfer agreement may be needed in some cases.

Data and code availability

- Structure of the TRPM2 channel (Apo and ADPR and Ca^{2+} bound state) and 3D structure of bilirubin that were used for molecular docking simulation were downloaded from Protein Data Bank and PubChem compound respectively (Details in key resources table). Additional data reported in this paper will be shared by the lead contact upon request.
- This paper does not report original code.

- Any additional information required to reanalyze the data reported in this work paper is available from the lead contact up request.

EXPERIMENTAL MODEL AND SUBJECT DETAILS

Ethical approval for data analysis of stroke patient blood and MRI

For hospitalized stroke patients, blood and MRI were used for diagnosis purpose. The levels of blood total bilirubin (TB), direct bilirubin (DB), alanine aminotransferase (ALT), and aspartate aminotransferase (AST) concentration were measured in clinical biochemistry laboratory, The Sixth People's Hospital of Shanghai. All enrolled participants underwent a head MRI (Achieva 3.0 T MRI system, Philips Healthcare, Amsterdam, The Netherlands) scan within 72 hours after inpatient at the Department of Radiology. Clinical data collection and analyses from human subjects were approved by the Institutional Ethic Committee of the Sixth People's Hospital of Shanghai (Approval NO: 2020-232).

Animal and ethical approval

The *Trpm2*$^{-/-}$ C57BL/6 mice were originally generated in the University of Leeds as described previously.[81] The D1069 site of the hTRPM2 channel is conserved, which correspond to D1066 in mice (Table S4). *D1066A* KI mice were constructed by CRISPR/Cas9 technology. The guide RNA targeting exon23 of TRPM2 gene, Cas9 mRNA and donor DNA recombinant plasmids were injected into fertilized eggs by microinjections. The homozygous transgenic mice from candidate lines were screened, acquired after several rounds hybridization and validated by PCR and sequencing. All mice (both male & female, aged 6-8 weeks) with matched genetic background were kept in specific-pathogen-free (SPF) grade environments with appropriate temperatures and normal circadian rhythms (12-hour light-dark cycle) while being provided with adequate food and drinking water. All the experiments were conducted in conformity with the institutional guidelines for the care and use of animals, and experimental protocols were approved by the Ethics Committee of the Sixth People's Hospital of Shanghai and Shanghai Jiao Tong University School of Medicine. Throughout the experiment, all efforts were made to minimize animal suffering.

Cell culture and transfection of human embryonic kidney 293 cell line

Human embryonic kidney 293 (HEK293) cells with tetracycline-inducible expression of human TRPM2 channel (hTRPM2) were cultured in a mixed medium containing DMEM/ F-12 (Gibco, USA) and 10% fetal bovine serum (Gibco, USA) as well as blasticidin (50 μg/ml, Invivogen, USA) and zeocin (0.4 mg/ml, Invivogen, USA). The expression of hTRPM2 was induced by substituting blasticin and zeocin for tetracycline (1 μg/ml, Sigma, USA) 24-48 hours before use. TRPM2 channel mutants were transiently expressed in HEK293T cells by transfection with using Lipofectamine 3000. Briefly, HEK293T cells were transiently transfected with cDNAs encoding the mutant hTRPM2 channel. The cDNA for GFP was co-transfected as a marker for identification of the transfected cells for electrophysiological experiments 16-24 hours after transfection. All the cells were seeded on 96-well coverslips (3 x 3 mm, WHB) and cultured at 37 °C under a humidified atmosphere containing 5% CO_2.

METHOD DETAILS

Enrolled clinical data

Clinical data were collected from the department of neurology, Shanghai Sixth People's Hospital Affiliated to Shanghai Jiao Tong University School of Medicine, which was composed of 944 stroke patients hospitalized from November 2019 to May 2020 (age: 25 to 95, both man and woman). MRI examination and bilirubin concentration were the results of the first detection of stroke patients within 72 hours after hospitalization. The exclusive criteria were stroke patients (1) with hemorrhagic etiology, (2) without brain MRI, (3) with brainstem or cerebellum ischemic/infarction, (4) with cerebrovascular obstructed, but DWI MRI images did not show abnormalities, (5) with abnormal liver function by laboratory biochemical measurements (6) incomplete and missing clinical data (Figure S1). In total, clinical data from 214 subjects were obtained and divided into two groups based on the serum total bilirubin levels, including stroke patients with normal bilirubin concentration (Normal group, n = 167; Male: 106 and Female: 61; Average age 71), stroke patients with abnormal bilirubin concentration (HB group, n = 47; Male: 36 and Female: 11; Average age 68). Patients in the HB group with traceable serum biochemistry in their previous hospitalization history were used to compare changes in bilirubin levels before and after stroke. Infarct volumes were calculated by Osirix 12.0 software in diffusion weighted imaging (DWI) sequence and clinical data were analyzed by two individuals independently.

Preparation of brain slices

Brain slices containing cortical neurons from mice of both sexes were prepared for electrophysiology as previously described.[82] Briefly, all the adult mice aged 6-8 weeks were first anesthetized with sodium pentobarbital (55 mg/kg, i.p.) and then decapitated. The brains of *Trpm2*$^{+/+}$ and *Trpm2*$^{-/-}$ mice were quickly but carefully removed and immersed into ice-cold oxygenated cutting solution (95% O_2 and 5% CO_2), dissected, and sectioned at a thickness of 300 μM using a micro slicer (VT-1000S, Leica Microsystems, Nussloch, Germany). Slices were transferred to the incubation solution to recovery at 37 °C for 40 minutes and then transferred to a recording chamber at room temperature (21-26 °C) before use.

Transient middle cerebral occlusion (tMCAO) animal model

tMCAO was introduced as described previously.[41] In brief, C57BL/6 mice, either *Trpm2*$^{+/+}$, *Trpm2*$^{-/-}$ and *D1066A* (age: 6-8 weeks, weight: 20-25 g, both male and female), were anesthetized using a 2% isoflurane-oxygen mixture for induction and 1.5% for maintenance. Bilirubin was first dissolved in 1 M NaOH solution, and its pH value was adjusted back to 7.4-8.0 by titrations with 1 M HCl. Before the operation, bilirubin (50 μg/g) and saline were injected intraperitoneally. tMCAO model was achieved by inserting a monofilament suture (RWD Life Science) into the right MCA via the internal carotid artery. MCA embolization lasted for 30 min, and body temperature was maintained at 37 °C using a heated blanket. Adequate ischemia was confirmed by continuous laser Doppler flowmetry (moor FLPI-2). Animals that did not have a significant reduction of blood flow less than 30% baseline values during MCAO were excluded. When the surgery was finished, mice were placed on another 37 °C heating blanket till they regained consciousness and then returned to the cage. The blood pressure, body temperature and serum biochemical markers were monitored in mice before and after tMCAO surgery (Figure S2)

Laser speckle imaging

Mice were anaesthetized by 1% isoflurane and their head were restrained in a stereotaxic cylinder frame to minimize breathing motion. The scalp and the skull fascia were gently incised down the midline and peeled to the side. Saline was titrated onto the skull to maintain moist. Laser speckle images were recorded with a CMOS camera before MCAO, 15 min after occlusion and 15 min after reperfusion. For each animal, three sets of raw speckle images were acquired in <15 s (250 frames in each set; image width, 752 pixels; image height, 580 pixels; exposure time, 20 ms). A speckle contrast image was calculated from each raw speckle image using a sliding grid of 2.5 mm × 2.5 mm. A mean speckle contrast image was calculated for each set and used to estimate the relative cerebral blood flow (rCBF). The rCBF in the ipsilateral (ischemic) hemisphere was normalized by the mean rCBF in the contralateral (non-ischemic) hemisphere. Speckle images were obtained and processed by the software mFLPI2MeasV2.0, rCBF data from all pooled hemispheres were obtained by the software moorFLPIReviewV50. All analyses were randomized.

Infarct volume measurement

24 hours or 7 days after tMCAO, animals were anesthetized using a 2% isoflurane-oxygen mixture. Brains were extracted and coronally sectioned into 1 mm slices, which were then stained with 2% 2,3,5-triphenyltetrazolium chloride (TTC) for 20 min at 37 °C. The infarct volume was analyzed using ImageJ and the infarct volumes were calculated according to the following formula: Corrected infarct volume (%) = [contralateral hemisphere volume − (ipsilateral hemisphere volume − infarct volume)] / contralateral hemisphere volume × 100%.

Total bilirubin (TB) and unconjugated bilirubin (UCB) measurement

Trpm2$^{+/+}$, *Trpm2*$^{-/-}$ and *D1066A* C57BL/6 mice were anesthetized by intraperitoneal injection of pentobarbital (55 mg/kg) 24 hours before tMCAO operation, and then fixed in the stereotactic setup (RWD Life Science). The skin above the skull from the base of the neck up to in between the eyes was cut open. A hole directly above the right ventricle was made with a grinding drill to allow insertion of the micro syringe into the lateral ventricle (coordinates: 1.1 mm laterally to the right and 0.5 mm posterior of the bregma, 2.5 mm deep). CSF was collected by using the micro injection pump (speed: 0.2 μl/min), after which the syringe was withdrawn, and the skin was sutured. The mice were removed from the stereotactic setup and 50 μl blood was taken through the orbital venous plexus. Mice were then placed on 37 °C heating blanket till they regained consciousness and returned to the cage. 1 or 24 hours after tMCAO, animals were anesthetized using a 2% isoflurane-oxygen mixture. CSF and blood were collected in the same way before sacrifice. All samples were centrifuged at 3000 r/min. Total bilirubin concentration of supernatants was measured with the Bilirubin Reagent Kit (Sigma). Samples were transferred to a 96-plates and mixed with reaction solution before measurements of their absorbance were made at 530 nm 10 minutes later. UCB concentration was measured with UnaG, a bilirubin-inducible fluorescent protein from Japanese eel muscle. For standard calibration curve of fluorescence intensity, a 100 μl reaction mixture containing 50 μl UnaG solution (1 μM) and 50 μl artificial bilirubin solution with concentration gradient (1.4278 μM, 0.7139 μM, 0.3573 μM, 0.1785 μM, 0.0893 μM and 0 μM) was prepared. After 10 minutes of reaction, the fluorescence intensity was detected by microplate reader (Synergy H1M, Bio Tek) with fluorescence filters for excitation and emission wavelengths of 485 and 528 nm, respectively. Serum and CSF samples were diluted 20-fold with PBS and fluorescence intensity was measured. The UCB levels were extrapolated from the standard curve.

Oxygen-glucose deprivation model in brain slices

Mice were anesthetized with isoflurane, brain slices containing cortical neurons were sectioned at a thickness of 300 μM and incubated (37 °C) for 30 min.[82] The slices were first washed with glucose-free artificial cerebrospinal fluid (ACSF) solution for 10 min prior to OGD to deplete the remaining glucose from extracellular space. Finally, the brain slices were transferred to a 24-well plate containing glucose-free ACSF, and the OGD experiment was performed in a hypoxia incubator (95% N_2, 5% CO_2, 37 °C). During the hypoxia process, 5 μl of glucose-free ACSF was collected at different time points for the measurements of UCB concentration.

Immunofluorescence

All mice were anesthetized with 1% pentobarbital (0.04 ml/10 g) before cutting the sternum to expose heart. A needle was inserted into the left ventricle to perfuse the pre-oxygenated cold ACSF with low molecular weight heparin. The ACSF was perfused until the

tail, limbs and liver of the pops turned into pale (during which the heart was kept beating) before being switched to the lavage solution containing 4% paraformaldehyde, and the brain tissue was fully fixed by perfusion for about 15 min. After fixation, brain was embedded in 2% agarose and sectioned into slices (100 μm) after complete coagulation. Brain slices were immersed in 1% Triton X-100 for 30 min, and then blocked with 10% bovine serum albumin (BSA, Sangon biotech) for 1 hour. Co-localization was accomplished using four-color multiple fluorescent immunohistochemical staining kit (abs50028, Absin). Briefly, the primary antibodies were diluted with buffer (Beyotime), added, and then incubated at 4 °C overnight. The slices were then rinsed with TBST 3 times and incubated with horseradish peroxidase (HRP)-labeled secondary antibody (goat anti-rabbit) for 15 min followed by a 10 min incubation with tyramide signal amplification (TSA) monochromatic fluorescent dye. Finally, the brain slices were immersed in antibody eluate (abs994, Absin) for 30 min, and subsequently wash with PBS for 3 times. These steps with a second/third/fourth primary antibody were repeated to achieve co-staining of multiple markers. The primary antibodies used for immunofluorescence are mouse anti-NeuN (1:200, ab104224, Abcam), rabbit anti-TRPM2, rabbit anti-BVR (1:200, ADI-OSA-400-E, Enzo life science), rabbit anti-heme oxygenase-1 (1:500, ab68477, Abcam) and rabbit anti-S100 beta (ab52642, 1:100, Abcam). 4'6, -Diamidino-2-phenylindole (DAPI) (2 mg/ml, Sigma) was used for nuclear staining.

Molecular docking simulations

Structure of TRPM2 was obtained from Protein Data Bank (PDB ID: 6PUS, 6PUO). 3D structure of bilirubin was obtained from PubChem compound (PubChem CID: 5280352). Molecular docking was generated using the Schrödinger Maestro software suite (Schrödinger, 2020-3). Prior to docking, protein was processed using the Protein Preparation Wizard for adding missing residues, removing waters, optimizing H-bond and energy (OPLS3e force field). Ligand was optimized using OPLS3e force field in LigPrep module. Protein and ligand protonation states at pH 7.4 ± 0.2 were sampled using Epik. Ligand was docked to a picked residue in a grid box with dimensions of 25 x 25 x 25 $Å^3$. Extra-precision docking (Glide XP) was performed with flexible ligand sampling, and post-docking minimization was performed to generate a maximum of 10 poses per ligand within the Glide program, the docking conformation with a highest docking score was analyzed to identify critical molecular interactions between different ligand moieties and amino acid residuals of the binding pocket for guided site-directed mutagenesis.

Molecular dynamics (MD) simulation

For MD simulation, only the transmembrane domain of TRPM2 was selected, the best pose of TRPM2-bilirubin complex was selected from docking results and the ligand-bound protein systems were built with the CHARMM-GUI online membrane builder. The ligand-protein complex was inserted into a lipid bilayer composed of 180 1-palmitoyl-2-oleoylsn-glycero-3-phosphocholine (POPC) molecules solvated with a 22.5 Å-thick layer of 150 mM NaCl aqueous solution (Figure S10). To investigate the stability of the docked ligand-protein poses, 50-ns simulations were performed. After 25000 steps of minimization, the systems were equilibrated in six steps as default from CHARMM-GUI lipid bilayer outputs, followed by 50-ns production run. All simulations were done by using the GROMACS 2020.3, CHARMM36m force fields with WYF parameters for the protein, and CHARMM General Force Field (CGenFF) ligand, CHARMM36 force fields for lipids, and the TIP3P model for water. These simulations were carried out using isothermal-isobaric (NPT) ensembles at a constant temperature of 303.15 K. The simulation trajectories were analyzed for structural stability using root-mean-square deviation (RMSD) and root-mean-square fluctuation (RMSF) calculations. MM-PBSA calculations on MD simulation trajectories were performed with a gmx_mmpbsa bash script using solvent-accessible surface area (SASA) as the model for non-polar solvation energy. The best pose of TRPM2-bilirubin or XAME complex was selected from molecular docking for binding pocket analysis by Fpocket 2.0 software. A dpocket program was performed to produce pocket parameter information using default settings.

Cell death assess with calcein-am/PI co-staining

Adult (6-8 weeks) *Trpm2*$^{+/+}$ brain slices containing cortical neurons were sectioned at a thickness of 300 μM. The brain slices were incubated with bilirubin (Bil) and bilirubin+A23 (Bil+A23) for 1 hour at 37 °C, adding calcein-am (1 μM) and PI (2 μM) for the last 15 minutes, and then washed for three times before being fixed with 4% paraformaldehyde (Solarbio) in a dark container for 40 minutes. Finally, live and died nuclei was detected by microscopic examination at 20 × magnification. The number of staining nuclei was measured by using the Image-J software.

Electrophysiology

Coronal cortical slices were transferred into the recording chamber and continuously perfused at room temperature with ACSF oxygenated with 95% O_2 and 5% CO_2. Cortical pyramidal neurons were visualized by an upright microscope (Carl Zeiss) equipped with a CCD monochrome video camera (IR-1000, DAGE-MTI) and identified by their localization in Layer V. Electrophysiology data were sampled (50 kHz) and lowpass filtered (5kHz four-pole Bessel filter) by a MultiClamp 700B dual channel amplifier (Molecular Devices). The patch electrodes were pulled from borosilicate capillary glass (1.5 mm outer diameter; 0.86 mm inner diameter; World Precision Instruments, USA) with a horizontal multi-step micropipette puller (Model, P-1000, Sutter Instrument). The electrode when filled with intracellular solution had a resistance between 3 and 8 MΩ. After forming >1 GΩ resistance seal in cell-attached configuration, we broke the patch membrane and kept the neurons at -64 mV and then performed whole-cell current-clamp recordings. Cells requiring holding current > -150 pA at -60 mV were discarded. The resting membrane potential (Vrest) was tested in I = 0 configuration. Intrinsic excitability was tested by 500 ms long current steps between -200 and +400 pA with 50-pA increments in the presence of blockers for

AMPA, NMDA, and GABA receptors (APV, 50 μM; NBQX, 10 μM; bicuculine 10 μM, strychnine 1 μM). Only one recording was conducted per brain slice. For whole-cell or single channel recordings from transfected HEK-293T cells, the coverslips were placed in a culture dish (35 × 35 mm, Sorfa) containing HEPES based extracellular solution (ECS) and recorded by patch-clamp technique. Cells were visualized by using the phase-contrast mode of an inverted microscope (TE-2000U; Nikon, Japan) and the patch pipette was positioned on the cells using a motorized micromanipulator (MP-225; Sutter) under the microscope. The transfected cells were voltage-clamped at 0 mV and subjected to voltage ramps from -100 mV to +100 mV (500 ms duration) every 5 s. The basal ramp current (i.e., the trace when the leak current is stable after membrane rupture) before channel activation were used for leak subtraction of all subsequent ramp currents. Each coverslip was recorded only once with different drug exposures. Single-channel recordings were carried out under outside-out configuration, using pipettes with resistance of 6-8 MΩ. The excised patches were voltage-clamped from -80 mV to +80 mV (in 20 mV increasement) to generate the current-voltage (I-V) relationship of single channel currents from which their main conductance was calculated from the slope by linear regression of the mean current amplitude at different potentials. Single-channel recordings of the cortical neurons were performed by adding 4-AP, TEA-Cl, CsCl2 and TTX to block sodium channel, potassium channel and calcium channel.

Solutions

The cutting solution for brain slices contained the following (in mM): 87 NaCl, 75 sucrose, 2.5 KCl, 10 glucose, 1.25 NaH_2PO_4, 2 Na-pyruvate, 3 myo-inositol, 0.5 ascorbic acid, 26 $NaHCO_3$,7 $MgCl_2$, and 0.1 $CaCl_2$ (pH 7.4; 290-320 mOsm). ACSF for incubation and current-clamp recordings contained (in mM): 124 NaCl, 5 KCl, 1.2 $KH2PO_4$, 1.3 $MgCl_2$, 2.4 $CaCl_2$, 24 $NaHCO_3$, and 10 glucose, pH 7.4 (osmolality of 290-320 mOsm) saturated with 95% O_2 and 5% CO_2. For OGD, external glucose was replaced with sucrose to obtain glucose-free ACSF. In some recordings, synaptic blockers (APV, NBQX, bicuculine and strychnine) were added to isolate intrinsic firings of cortical neurons. Intracellular solution for current-clamp recordings contained (in mM): 130 K-gluconate, 5 KCl, 4 $MgCl_2$, 0.6 EGTA, 2 Mg-ATP, 0.5 GTP, 10 HEPES, and 10 creatine phosphate disodium salt with pH 7.4, and the osmolality was adjusted to 290-310 mOsm. Whole cell recordings for transfected HEK-293T cells were conducted in standard ECS containing (in mM): 145 NaCl, 5.6 KCl, 2 $MgCl_2$, 1.2 $CaCl_2$, 10 HEPES, 10 glucose and pH adjusted to 7.4 by NaOH (290-320 mOsm). And pipette solution contains (in mM): 147 NaCl, 1 $MgCl_2$, 1 EGTA (or 30 mM BAPTA) and 10 HEPES with pH 7.4 and osmolality 290-320 mOsm. In the case for 100 μM free Ca^{2+}, 1.1 mM $CaCl_2$ were added to the pipette solution with 1 mM EGTA (calculated by Maxchelator). Single-channel recordings were made with the same intra- and extracellular solution as for whole-cell recordings. Since bilirubin is easily decomposed by light, a 1 mM storage solution was prepared to be added to a light-sheltered reservoir at the required concentration immediately before infusion to prevent degradation and denaturation. All experiments were performed at room temperature (21-26 °C), and all reagents were purchased from Sigma-Aldrich, unless otherwise indicated.

QUANTIFICATION AND STATISTICAL ANALYSIS

Electrophysiological data were analyzed using Clampfit 10.5 software (Axon Instruments), PatchMaster v2x90.5 (HEKA Elektronik) and MiniAnalysis (Synaptosoft, NT, USA). The input-output relationships of cortical neuron were fitted with the Boltzman function: $S = S_{min}+(S_{max}-S_{min})/(1 + exp[(I_{50}-I)/k])$, in which S is the spike number, S_{max} is the maximal spike number, S_{min} is the minimum spike number, I is the magnitude of injected currents, I_{50} is the half current magnitude between S_{min} and S_{max}, and k is the slope factor. All-points analyses of single-channel recording were preceded by Clampfit's Gaussian filter (1/2 kHz) to reduce the noise. GraphPad Prism 8 (GraphPad Software) and Adobe Illustration (Adobe System Inc.) were used for graphic representation. Dose response curve (stimulation) was fitted with Hill equation: $I= I_{max}/(1+10\wedge((LogEC_{50}-X)^{nH}))$, in which I denotes the current amplitude at concentration X; EC_{50} stands for the concentration required to generate 50% of the maximal current (I_{max}) whereas nH represents Hill coefficient. Dose response curve (inhibition) was fitted with Hill equation: $Y=In_{min}+(In_{max}-In_{min})/(1+(IC_{50}/X)\wedge nH)$, in which Y denotes the degree of inhibition at concentration X; IC_{50} stands for the half concentration required to produce 50% antagonistic effect of the maximal inhibition (In_{max}) whereas nH represents Hill coefficient. Statistical analyses were performed using SPSS 25.0 software (SPSS Inc.). All numerical results were expressed as means ± SEM (unless otherwise stated). Independent-samples t-test or paired Student's t-test were used for pairwise comparisons. Multiple groups were compared by one-way analysis of variance (ANOVA) followed by LSD multiple comparison tests for post hoc analysis.

Neuron, Volume *111*

Supplemental information

Bilirubin gates the TRPM2 channel as a direct agonist to exacerbate ischemic brain damage

Han-Wei Liu, Li-Na Gong, Ke Lai, Xia-Fei Yu, Zhen-Qi Liu, Ming-Xian Li, Xin-Lu Yin, Min Liang, Hao-Song Shi, Lin-Hua Jiang, Wei Yang, Hai-Bo Shi, Lu-Yang Wang, and Shan-Kai Yin

Figure S1

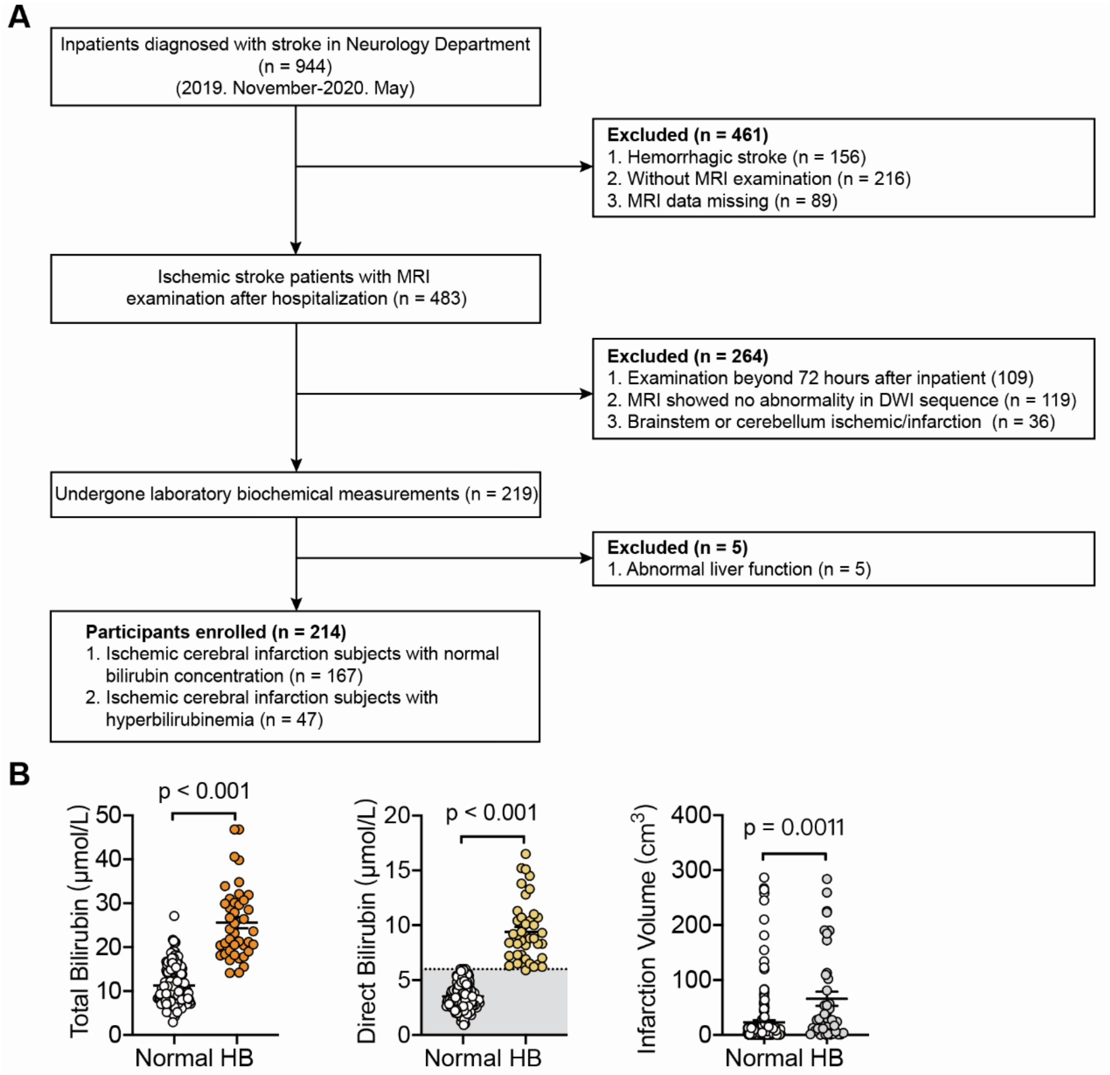

Figure S1. Enrollment flow chart for the study population, Related to Figure 1.

(A) 944 subjects diagnosed with stroke in neurology department from November 2019 to May 2020 were recruited in this study cohort. 214 ischemic stroke inpatients met the inclusion criteria and were enrolled in this study.

(B) Summary plots of TB, DB and infarct volume of enrolled patients which were grouped according to the DB concentration (Normal: DB ≤ 6 μmol/L, n = 173; HB: DB > 6 μmol/L, n = 41).

Figure S2

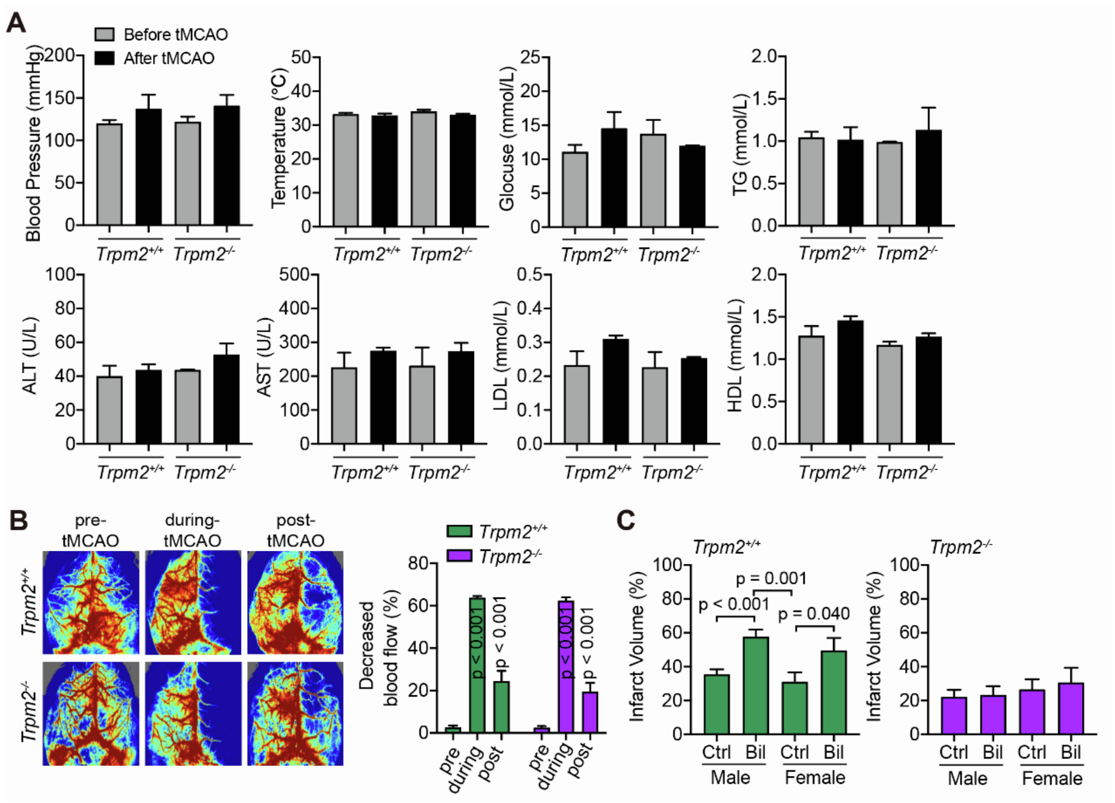

Figure S2. Genotype-dependent differences in the infract volume of *Trpm2*$^{+/+}$ and *Trpm2*$^{-/-}$ mice both sexes by tMCAO surgery without affecting major physiological and biochemical parameters, Related to Figure 2.

(A) Measurement results of blood pressure, body temperature and serum biochemical indexes (triglyceride, TG; alanine aminotransferase, ALT; aspartate aminotransferase, AST; low density lipoprotein, LDL; high density lipoprotein, HDL) before and after tMCAO surgery (n = 3)

(B) Representative blood flow images of the brain in mice before and after tMCAO surgery and flux (%) was quantified with a laser Doppler flowmetry probe (n = 5).

(C) Summary plots of the infarction volume of *Trpm2*$^{+/+}$ and *Trpm2*$^{-/-}$ male and female mice 24h after tMCAO surgery. In *Trpm2*$^{+/+}$ mice, bilirubin significantly increased the infarct volume in both male and female mice (Male: Ctrl: 35.42 ± 3.01%, Bil: 57.61 ± 4.24%, n = 10; Female: Ctrl: 31.08 ± 5.55%, Bil: 49.56 ± 7.40%, n = 4). Female mice showed slightly smaller infract volume which was not statistical different from male mice. Bilirubin showed little effect on aggravating brain injury in *Trpm2*$^{-/-}$ mice of both sexes (Male: Ctrl: 22.11 ± 4.14%, Bil: 23.22 ± 5.25%, n = 10; Female: Ctrl: 26.50 ± 6.02%, Bil: 30.63 ± 8.71%, n = 5).

Error bars represent means ± SEM; one-way ANOVA with post hoc LSD test.

Figure S3

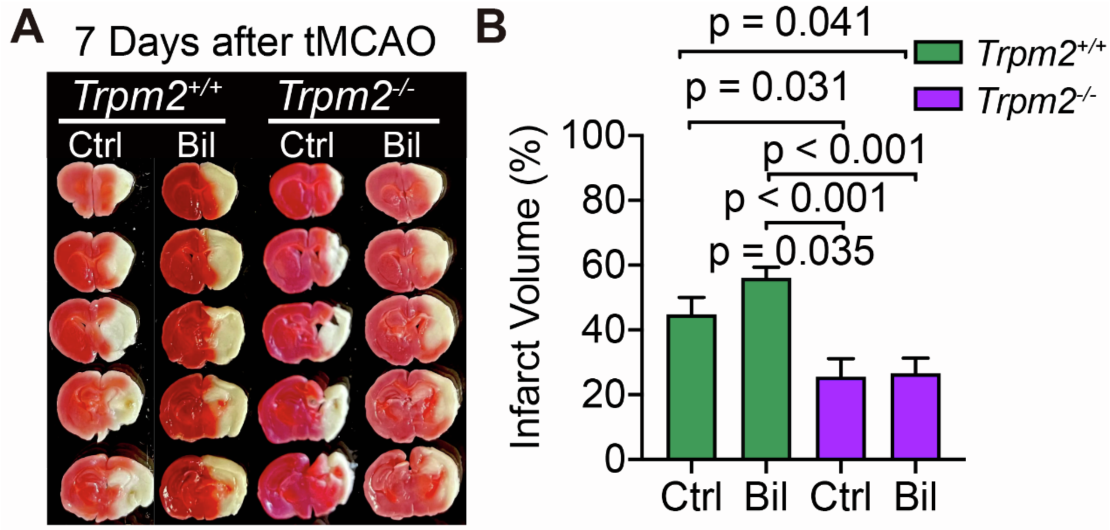

Figure S3. Transient hyperbilirubinemia aggravates long-term ischemic brain injury in genotype-specific manner, Related to Figure 2.

(A) Representative images of brain sections by TTC staining of *Trpm2*$^{+/+}$ and *Trpm2*$^{-/-}$ mice 7 days after the tMCAO surgery.

(B) Summary data showing normalized infarct volumes of Ctrl and Bil group in *Trpm2*$^{+/+}$ and *Trpm2*$^{-/-}$ mice (n=5).

Error bars represent means ± SEM; one-way ANOVA with post hoc LSD test.

Figure S4

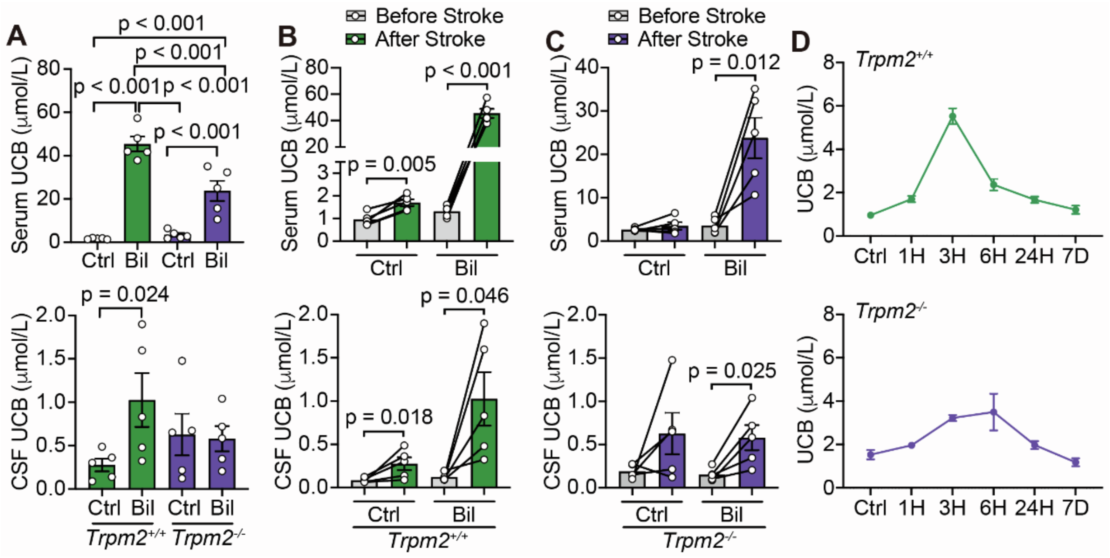

Figure S4. Temporal profiles of UCB concentrations after tMCAO surgery, Related to Figure 2.

(A) Summary data showing serum and CSF UCB concentrations of Ctrl and Bil group in *Trpm2*$^{+/+}$ and *Trpm2*$^{-/-}$ mice 1hour after tMCAO surgery (n= 5).

(B and C) Comparison of UCB concentrations in serum and CSF of in *Trpm2*$^{+/+}$ and *Trpm2*$^{-/-}$ mice in Ctrl and Bil group before and after ischemia-reperfusion injury (n = 5).

(D) Summary data showing the time-course of UCB concentrations in serum before (Ctrl) and after the tMCAO surgery in Trpm2+/+ and Trpm2-/- mice, respectively (n = 3 to 10) (basal bilirubin concentrations were indicated by Ctrl).

Error bars represent means ± SEM; paired Student's t test, one-way ANOVA with post hoc LSD test.

Figure S5

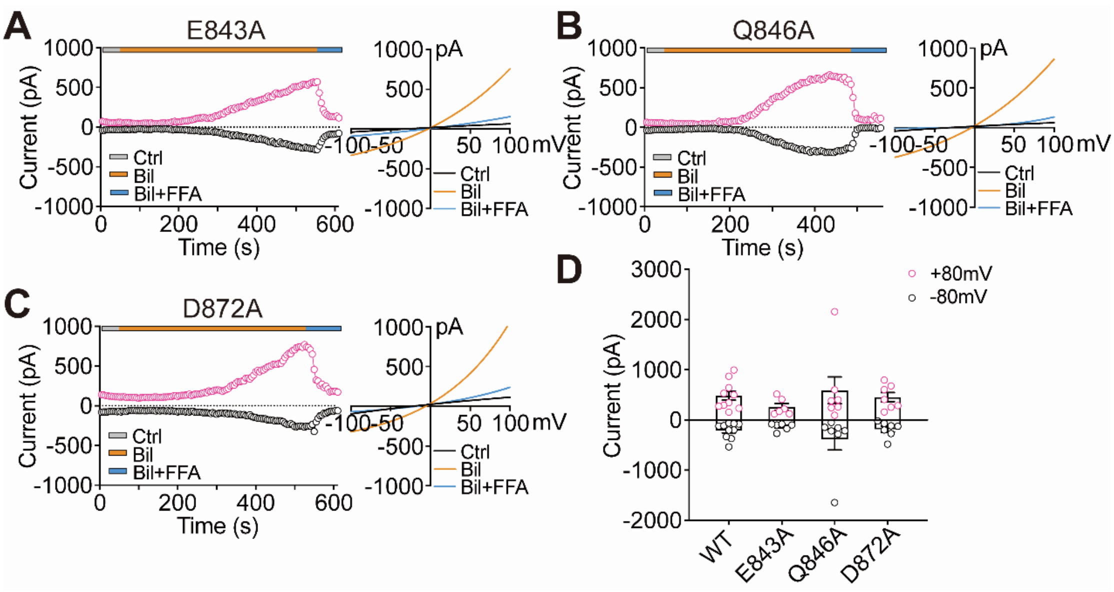

Figure S5. Bilirubin activates TRPM2 currents in Ca^{2+} binding site mutants, Related to Figure 4. (A to C) Representative time-course of currents at +80 mV (strawberry circle) and -80 mV (black circle) and ramp current traces transformed into superimposed I-V curves currents from Ca^{2+} binding site mutants before and after bilirubin application and FFA co-application.

(D) Summary data showing the maximum amplitude of currents (at +80 mV and -80 mV) in mutant TRPM2 channels activated by bilirubin in A to C relative to the that in WT channels (n = 6 to 13). Error bars represent means ± SEM; unpaired Student’s t test.

Figure S6

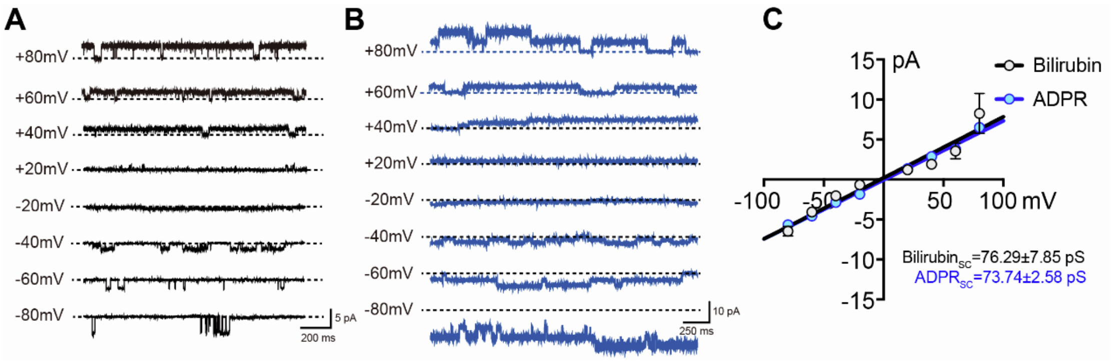

Figure S6. Comparable single channel conductance of the TRPM2 current activated by bilirubin and ADPR, Related to Figure 4.

(A and B) Representative TRPM2 single-channel recordings activated by bilirubin and ADPR from -80 mV to +80 mV.

(C) Mean single-channel current amplitudes activated by bilirubin and ADPR were plotted against membrane holding potentials and fitted with linear regression to calculate single channel conductance as given in the inset (n = 4 to 7).

Figure S7

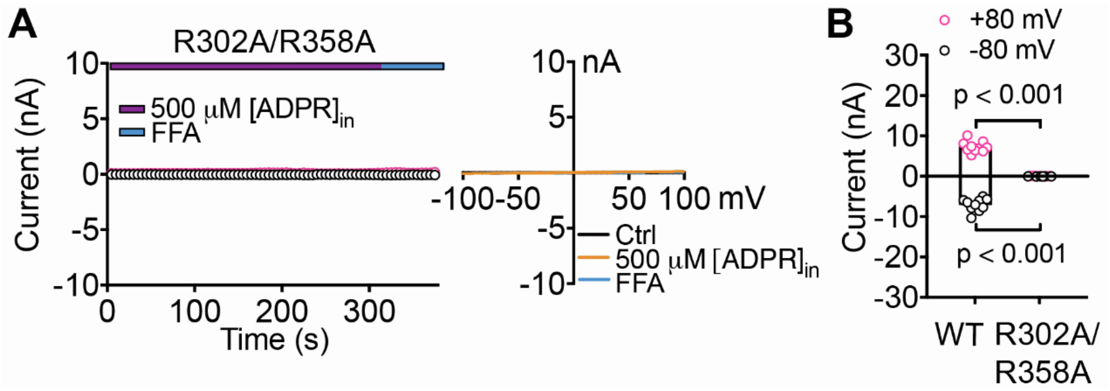

Figure S7. The R302A/R358A mutation abolishes ADPR activation of the TRPM2 channel, Related to Figure 6.

(A) Representative time-course of currents at +80 mV (strawberry circle) and -80 mV (black circle) and ramp current traces transformed into superimposed I-V curves currents R302A/R358A double mutant.

(B) Summary data showing the maximum amplitude of currents (at +80 mV and -80 mV) activated by ADPR in WT and R302A/R358A mutant TRPM2 channels (n = 5 and 10).

Error bars represent means ± SEM; unpaired Student's t test.

Figure S8

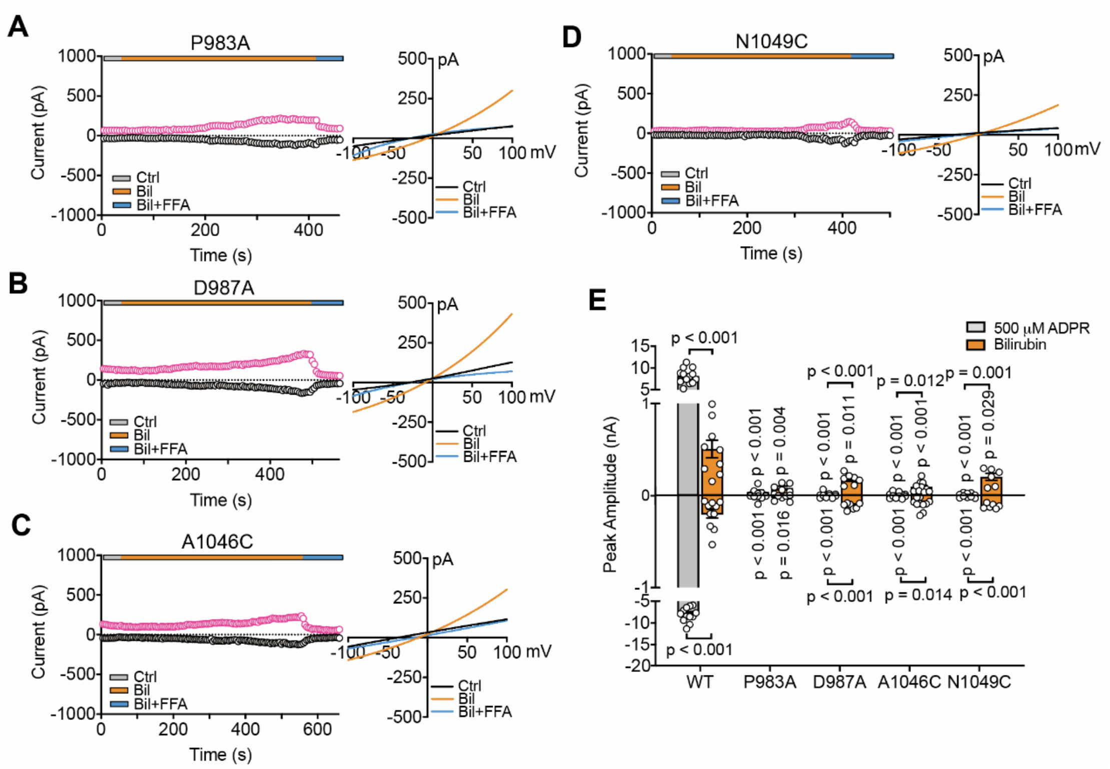

Figure S8. bilirubin activates TRPM2 pore domain mutants, Related to Figure 6.

(A to D) Representative time-course of currents at +80 mV (strawberry circle) and -80 mV (black circle) and superimposed I-V curves from ion-conducting pore mutants (top of the pore: P983A and D987A, bottom of the pore: A1046C and N1049C) before and after bilirubin application and FFA co-application.

(E) Summary data showing the maximum amplitude of currents (at +80 mV and -80 mV) activated by ADPR and bilirubin in WT TRPM2 channel and pore domain mutants (n = 6 to 13).

Error bars represent means ± SEM; unpaired Student's t test.

Figure S9

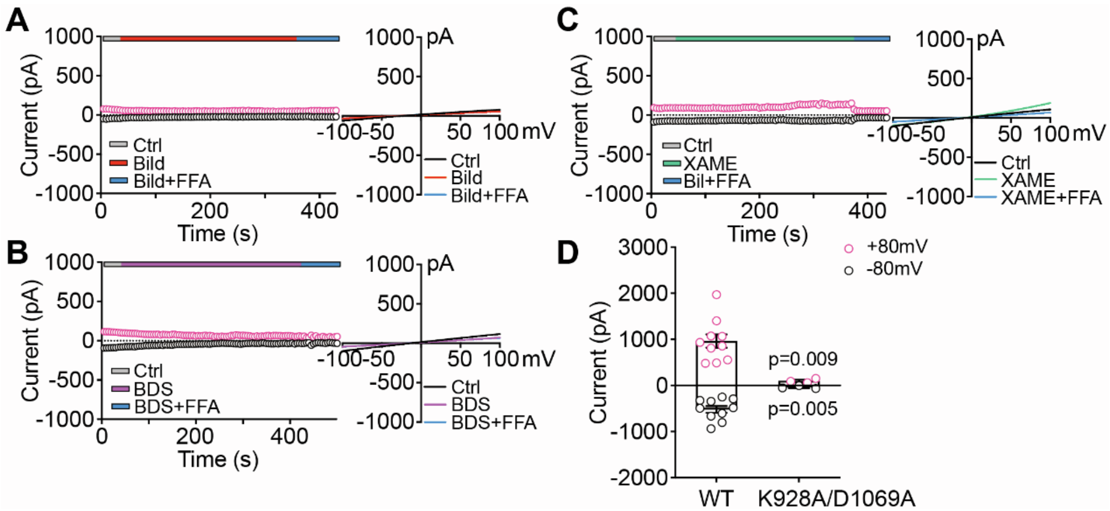

Figure S9. Derivatives of bilirubin fails to open TRPM2 channel in K928A/D1069A double mutant, Related to Figure 6.

(A to C) Representative time-course of currents at +80 mV (strawberry circle) and -80 mV (black circle) before and after bilirubin (9 μM), BDS (9 μM) and XAME (9 μM), followed by FFA co-application. I-V relationships before and after drug applications are overlayed and presented on the right panels.

(D) Summary data showing the maximum amplitude of currents activated by XAME in WT TRPM2 channel and K928A/D1069A mutant (n = 3 and 10).

Error bars represent means ± SEM; unpaired Student's t test.

Figure S10

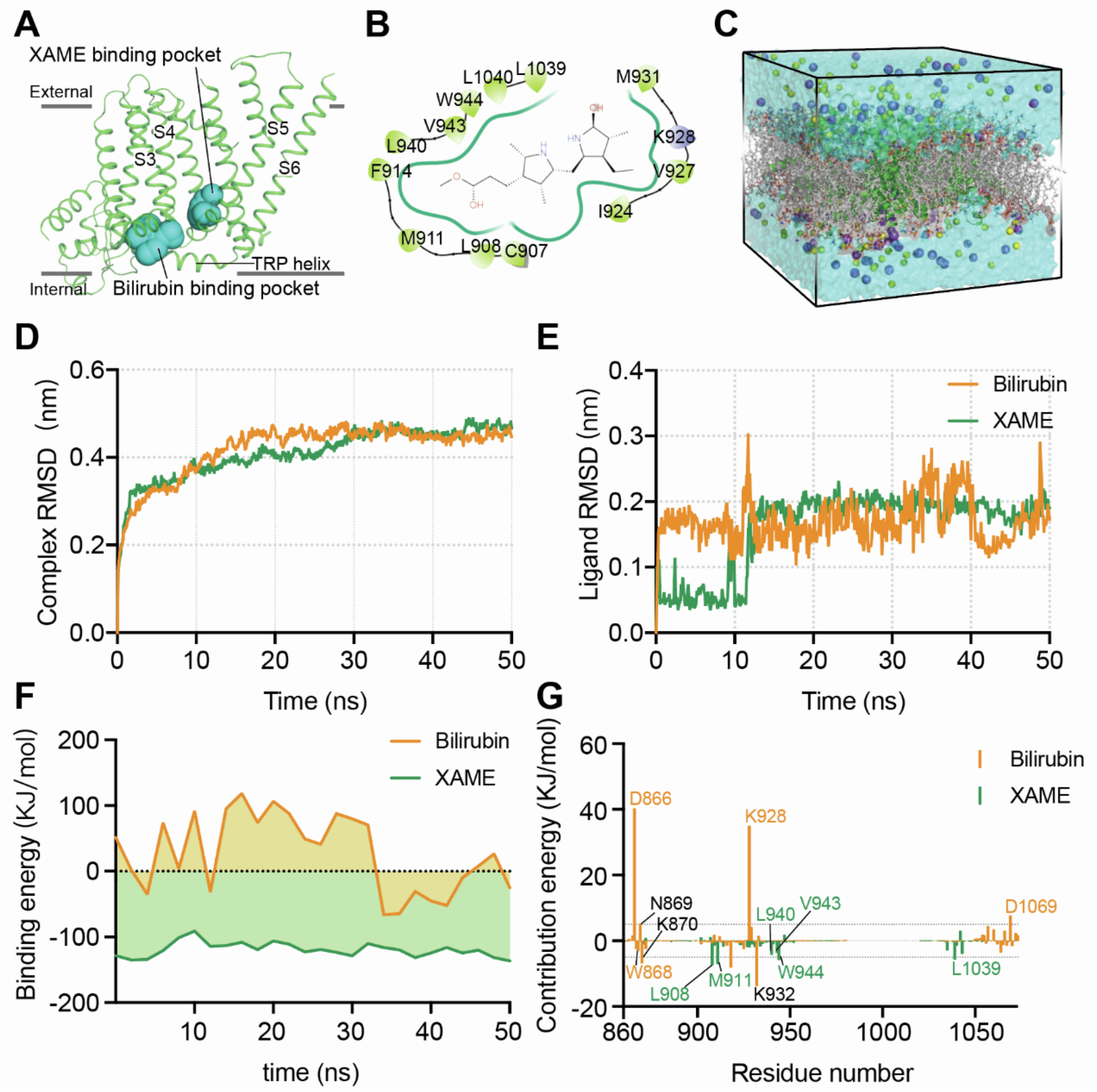

Figure S10. Molecular dynamics (MD) simulation analyses of the binding pockets of bilirubin and XAME and their interactions with the TRPM2 channel, Related to Figure 6.

(A) Relative positions of the binding pockets of bilirubin and XAME to the TRPM2 channel.

(B) 2D Interaction map between XAME and amino acid residues in the binding pocket.

(C) The simulated state diagram of the TRPM2-ligand complexes embedded in the cell membrane phospholipid bilayer.

(D and E) Summary results of structural stability analyses (root mean square deviation, RMSD) of channel-ligand complexes and ligands following bilirubin and XAME binding to TRPM2 channels in 50 ns bouts.

(F) Binding energy calculations showing a higher affinity for XAME than bilirubin to the TRPM2 channel.

(G) Summary analysis of binding energies contributed by the key residues within the binding pocket of TRPM2-bilirubin and TRPM2-XAME complexes (residues interact with bilirubin were shown in orange, and those of XAME were shown in green).

Figure S11

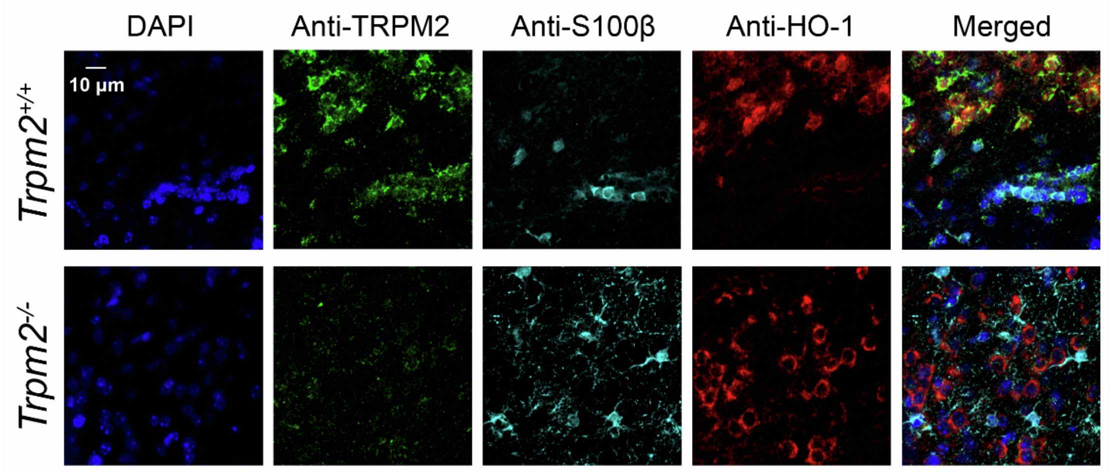

Figure S11. Astrocytes lack the expression of a key enzyme in bilirubin metabolism, Related to Figure 7.

Immunofluorescence co-localization imaging of astrocyte (anti-S100β), TRPM2 channels (anti-TRPM2), and heme oxygenase 1 (anti-HO-1) in cortical slices from $Trpm2^{+/+}$ and $Trpm2^{-/-}$ mice. Despite the presence the TRPM2 channel in neurons and astrocytes, HO-1, the key enzyme for bilirubin metabolism, was not colocalized with astrocytes.

Table S1

	n	Age (year)	Gender (male/n%)	TB (μmol/L)	DB (μmol/L)	Infarct volume (cm^3)
Normal group	167	71 (25-95)	106 (63.47%)	10.74 (2.90-17.90)	3.52 (0.90-9.00)	18.45 (0.06-267.04)
HB group	47	68 (32-88)	36 (76.60%)	25.43 (18.10-46.80)	8.65 (4.30-16.50)	76.42 (0.13-286.65)
RR	/	/	/	0-18.0	0-6.0	/

Table S1. Detail information for enrolled stroke patients, Related to Figure 1.
Normal group was defined as serum TB=0-18.0 μmol/L. HB group was defined as serum TB beyond the reference range (RR).

Table S2

Residue	E_{Vdw} (kcal/mol)	E_{coul} (kcal/mol)	E_{Hbond} (kcal/mol)	Dis (Å)	E_{total} (kcal/mol)
K928	1.166 ± 1.113	93.804 ± 10.215	0	1.182 ± 0.154	94.032 ± 9.067
D1069	7.164 ± 0.543	28.723 ± 2.942	0.505 ± 0.190	2.063 ± 0.160	36.392 ± 2.817
D866	3.219 ± 1.674	32.650 ± 3.531	0.246 ± 0.118	2.164 ± 0.194	36.116 ± 3.687
W868	3.984 ± 1.003	4.688 ± 2.322	0.174 ± 0.188	1.989 ± 0.335	8.845 ± 1.970

Table S2. Interaction scores between bilirubin and key residues of TRPM2 channel in molecular docking, Related to Figure 6.

In silico simulations between bilirubin with its binding pocket of TRPM2 channel. Bilirubin interacts with four residues among S3, S5 and TRP helix. Internal space constrains (Distance or Dis in Angstrom or Å) and molecular energetics of chemical bonds include Van Der Walls (Ev_{dw}) force, Coulombic (E_{coul}) force and hydrogen bond (E_{Hbond}) are showed in the table (distance and energy of the top 8 bilirubin poses interacting with TRPM2 residues are shown as mean ± s.d.). The spatial distance and cumulative interaction energy (E_{total}) between bilirubin and the binding sites were also calculated.

Table S3

	Bilirubin binding pocket	XAME binding pocket
Pocket volume (Å^3)	2039.860	1452.90
Ligand volume (Å^3)	529.150	352.440
Ligand-Pocket overlap percent (%)	100	100
Total score	22.701	25.885
Polarity score	7	3
Flexibility	0.391	0.566

Table S3. Information on the binding pockets of bilirubin and XAME with the TRPM2 channel, Related to Figures 6.

In MD simulations of interactions between bilirubin or XAME and the TRPM2 channel, only the transmembrane domain of TRPM2 was selected. The relevant parameters of the bilirubin and XAME binding pockets were analyzed separately, including pocket volume, ligand volume and ligand-pocket overlap percentage. The total score, pockets polarity and flexibility were also analyzed and given.

Table S4

Species	Gene ID	Peptide ID	Peptide length
Human (*Homo sapiens*)	ENSG00000142185	ENSP00000381023	1503aa
Mouse (*Mus musculus*)	ENSMUSG00000009292	ENSMUSP00000101040	1506aa
Partial Sequence Information (*Homo sapiens*)	3101 TCTTCACCAACATCCTGCTGCTCAACCTCCTCATCGCCATGTTCAACTACACCTTCCAGC 3160<br>1034 L - - F - - T - - N - - I - - L - - L - - L - - N - - L - - I - - A - - M - - F - - N - - Y - - T - - F - - Q - - 1053<br>3161 AGGTGCAGGAGCACACGGACCAGATTTGGAAGTTCCAGCGCCATGACCTGATCGAGGAGT 3220<br>1054 Q - - V - - Q - - E - - H - - T - - D - - Q - - I - - W - - K - - F - - Q - - R - - H - - D - - L - - I - - E - - E - - 1073<br>3221 ACCACGGCCGCCCCGCCGCGCCGCCCCCCTTCATCCTCCTCAGCCACCTGCAGCTCTTCA 3280<br>1075 Y - - H - - G - - R - - P - - A - - A - - P - - P - - P - - F - - I - - L - - L - - S - - H - - L - - Q - - L - - F - - 1093		
Partial Sequence Information (*Mus musculus*)	3122 TCATCGCCATGTTCAACTACACCTTCCAGGAGGTGCAGGAACACACAGACCAGATCTGGA 3181<br>1041 L - - I - - A - - M - - F - - N - - Y - - T - - F - - Q - - E - - V - - Q - - E - - H - - T - - D - - Q - - I - - W - - 1060<br>3182 AATTCCAGCGCCACGACCTGATCGAGGAGTACCATGGCCGTCCCCCGGCACCTCCCCCAC 3241<br>1061 K - - F - - Q - - R - - H - - D - - L - - I - - E - - E - - Y - - H - - G - - R - - P - - P - - A - - P - - P - - P - - 1080<br>3242 TCATCCTCCTCAGCCACCTGCAGCTCCTGATCAAGAGGATTGTCCTGAAGATCCCTGCCA 3301<br>1081 L - - I - - L - - L - - L - - S - - H - - L - - Q - - L - - L - - L - - K - - R - - I - - V - - L - - K - - I - - P - - A - - 1100		
Exon 23	CTACACCTTCCAGGAGGTGCAGGAACACACAGACCAGATCTGGAAATTCCAGCGCCACGACCTGATC<br>GAGGAGTACCATGGCCGTCCCCCGGCACCTCCCCCACTCATCCTCCTCAGCCACCTGCAGCTCCTGAT<br>CAAGAGGATTGTCCTGAAGATCCC TGCCAAGAGGCATAAGCAGCTCA		
Guide RNA	GGTACTCCTCGATCAGGTCG TGG		
Donor DNA	CTACACCTTCCAGGAGGTGCAGGAACACACAGACCAGATCTGGAAATTCCAGCGCCACGCCCTGATC<br>GAGGAGTACCATGGCCGTCCCCCGGCACCTCCCCCACTCATCCTCCTCAGCCACCTGCAGCTCCTGAT<br>CAAGAGGATTGTCCTGAAGATCCCTGCCAAGAG GCATAAGCAGCTCA		

Table S4. Related sequence information of transgenic mice, Related to Figure 8 and STAR Methods.

The human and mouse gene ID, peptide ID, peptide length and partial gene/peptide sequence information are shown, in which the gene sequence is shown in black, the peptide sequence is shown in blue, and the gene sequence and peptide sequence corresponding to D1069/D1066 are shown in green background and yellow background respectively. The sequence information of Guide RNA and Donor DNA designed for mouse Exon 23 are also listed, among which the genetically modified codons are marked with red underlines, and the mutated gene are marked with red background.
